# Cyclooctatetraenide-based single-ion magnets featuring bulky cyclopentadienyl ligand†

**DOI:** 10.1039/d2sc02560d

**Published:** 2022-08-30

**Authors:** Maciej Damian Korzyński, Moritz Bernhardt, Vladyslav Romankov, Jan Dreiser, Guy Matmon, Fabrice Pointillart, Boris Le Guennic, Olivier Cador, Christophe Copéret

**Affiliations:** Department of Chemistry and Applied Biosciences, ETH Zürich Vladimir-Prelog Weg 1-5/10 8093 Zürich Switzerland korzynski@inorg.chem.ethz.ch ccoperet@ethz.ch; Swiss Light Source, Paul Scherrer Institut 5232 Villigen PSI Switzerland; Univ Rennes, CNRS, ISCR (Institut des Sciences Chimiques de Rennes), UMR 6226 35000 Rennes France boris.leguennic@univ-rennes1.fr olivier.cador@univ-rennes1.fr

## Abstract

We report a family of organometallic rare-earth complexes with the general formula (COT)M(Cp^ttt^) (where (COT)^2−^ = cyclooctatetraenide, (Cp^ttt^)^−^ = 1,2,4-tri(*tert*-butyl)cyclopentadienide, M = Y(iii), Nd(iii), Dy(iii) and Er(iii)). Similarly to the prototypical Er(iii) analog featuring pentamethylcyclopentadienyl ligand (Cp*)^−^, (COT)Er(Cp^ttt^) behaves as a single-ion magnet. However, the introduction of the sterically demanding (Cp^ttt^)^−^ imposes geometric constraints that lead to a simplified magnetic relaxation behavior compared to the (Cp*)^−^ containing complexes. Consequently, (COT)Er(Cp^ttt^) can be viewed as a model representative of this organometallic single-ion magnet architecture. In addition, we demonstrate that the increased steric profile associated with the (Cp^ttt^)^−^ ligand permits preparation, structural characterization and interrogation of magnetic properties of the early-lanthanide complex, (COT)Nd(Cp^ttt^). Such a mononuclear derivative could not be obtained when a (Cp*)^−^ ligand was employed, a testament to larger ionic radius of this early lanthanide ion.

## Abstract 2

Application of steric control principles allows for simplification of the magnetic behavior of an iconic single-ion magnet architecture as well as the preparation of its previously inaccessible representative.

## Graphical abstract

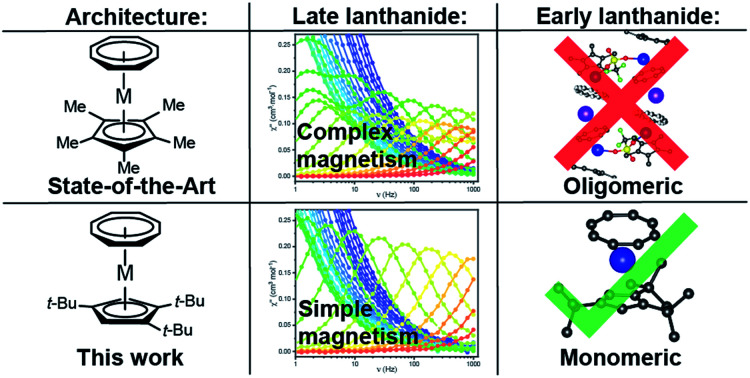

## Introduction

Single-ion magnets (SIMs) are a class of paramagnetic coordination complexes based primarily on lanthanides that exhibit intrinsic magnetic anisotropy.^1–4^ This property allows them to remain magnetized for a considerable amount of time following the removal of the external magnetic field, thus opening up the possibility for their application in long-term information storage devices such as hard-drive disks.^5^ In addition to data storage, the unique physics of SIMs make them suitable candidates for applications in quantum computing and spintronics.^6,7^ Eliciting the desired magnetic properties that warrant such applicability requires careful tailoring of the nature and the spatial distribution of ligands surrounding the lanthanide centers.^8^ Placement of the Ln(iii) ion in an appropriate crystal field ensures uniaxial anisotropy, leads to stabilization of the desired ground state of the metal ion, brings about a high barrier for the thermally activated relaxation of the magnetization, and minimizes the deleterious quantum tunneling of magnetization (QTM) that is prevalent in lanthanide-based SIMs.^9,10^ Conversely, deviation from the optimal coordination environment can easily compromise magnetic properties of the system.

In this context, Rinehart and colleagues introduced the metal–ligand pair anisotropy^11^ (MLPA) concept that identifies Ln(iii)–ligand pairings that form building blocks with a predictable orientation of the primary magnetic axis. Such robust baseline SIM characteristics of MLPA units permit a systematic investigation of the influence of the remainder of the coordination sphere on the magnetic properties. One important example of a MLPA concept is the [(COT)Er]^+^ unit comprised of prolate^12^ Er(iii) ion ligated with the cyclooctatetraenide ligand ((COT)^2−^) in which the anisotropy axis orients almost perpendicularly to the eight-membered aromatic ring. This motif is featured in multiple SIMs, most notably in the iconic [(COT)_2_Er]^−^ ^13^ and (COT)Er(Cp*) species^14^ ((Cp*)^−^ = 1,2,3,4,5-pentamethylcyclopentadienide anion). Despite its prominence, the molecular understanding of the latter complex's magnetic properties remains a challenge due to a non-trivial solid-state structure with effects such as weak intermolecular interactions that affect the relative orientation of (COT)^2−^ and (Cp*)^−^ rings as well as a static disorder of the (COT)^2−^. The latter effect, which leads to a coexistence of two distinct rotamers in the solid-state, is often invoked as an explanation for the two relaxation events that can be observed in measurements of the dynamic magnetic behavior for (COT)Er(Cp*),^15^ yet no synthetic efforts have been directed at evaluating this hypothesis.

With an eye toward the synthesis of a (COT)M(Cp) architecture exemplar that could offer a simplified structural, and consequently magnetic, behavior, we decided to leverage the concept of steric control. To date, only approaches based on non-negligible alteration of ligand electronics have been applied to tune the magnetic properties of this scaffold.^16–19^ In principle, steric control could be exerted by switching to bulkier analogs of either (COT)^2−^ or (Cp*)^−^. However, studies^20,21^ on SIMs containing the common bulkier (COT)^2−^ derivative, 1,4-bis(trimethylsilyl)cyclooctatetraenide, demonstrated that the additional substituents can affect the orientation of the anisotropy axis, and thus change the magnetic properties causing departure from MLPA concept. Thus, we decided to focus instead on increasing the bulk of the cyclopentadienyl fragment.^22,23^ We argued that utilization of a bulkier cyclopentadienyl-type ligand could lead to a more rigid scaffold, allowing us to suppress the structural effects affecting (COT)Er(Cp*). Furthermore, we hypothesized that the increased sterics of the ligand sphere could enable access to early lanthanide derivatives of (COT)M(Cp) architecture. While a diamagnetic lanthanum(iii) derivative featuring the (Cp*)^−^ has been reported, this compound is a polymer in the solid-state.^24^ This stands in a stark contrast with the reported late lanthanide derivatives and highlights the issues associated with the increased ionic radius of the early lanthanide ions. The synthetic challenges notwithstanding, early lanthanide ions can serve as attractive platforms for the assembly of SIMs. In particular, neodymium(iii) is desirable as it is a Kramers ion with a capability of accessing to relatively large *m*_J_ values.^25–27^ Most importantly, its higher natural abundance compared to late lanthanides makes it economically appealing from the industrial application perspective.^28,29^

Herein, we report the synthesis of a family of (COT)M(Cp^ttt^) complexes (M = Y(iii), Nd(iii), Dy(iii), and Er(iii)), where (Cp^ttt^)^−^ denotes bulky 1,2,4-tris(*tert*-butyl)cyclopentadienide anion that has already proved itself beneficial in SIM design.^30,31^ As expected based on MLPA theory, the prolate Er(iii) derivative behaves as SIM in contrast to the oblate Dy(iii) congener. Crucially, in the case of Er(iii) derivative the increased steric pressure associated with the (Cp^ttt^)^−^ allowed us to circumvent the crystal packing and disorder effects that plague the less bulky (Cp*)^−^ derivatives and permitted isolation of (COT)Er(Cp^ttt^) that exhibits monomodal relaxation behavior in the solid-state. Furthermore, the bulkier ligand architecture allowed us to isolate and probe magnetic properties of the early lanthanide congener of this series, (COT)Nd(Cp^ttt^). Notably, such derivative could not be obtained when (Cp*)^−^ was employed, a likely evidence of the larger ionic radius of Nd(iii) compared to late lanthanide ions.

## Results and discussion

### Preparation and characterization of the (COT)M(Cp^ttt^) complexes

The desired compounds were synthesized *via* the route utilized recently by some of us.^32^ Briefly, M(OTf)_3_ (where M = Y(iii), Nd(iii), Dy(iii), Er(iii); OTf^−^ = trifluoromethylsulfonate) salts were treated with K_2_COT in THF to yield a series of (COT)M(OTf)(THF)_*n*_ (*n* = 1 or 2) species. Treatment of these precursors with KCp^ttt^ in THF led to straightforward isolation of the late lanthanide and diamagnetic yttrium(iii) (COT)M(Cp^ttt^) species. The bulk purity of (COT)Y(Cp^ttt^) (Y^ttt^) has been confirmed by combination of elemental analysis (EA), attenuated total reflection Fourier-transform infrared spectroscopy (ATR-FTIR) as well as liquid-state ^1^H, ^13^C and ^89^Y nuclear magnetic resonance (NMR) spectroscopies (Fig. S1–S4†). The bulk purity of the (COT)Dy(Cp^ttt^) (Dy^ttt^) and (COT)Er(Cp^ttt^) (Er^ttt^) was established based on EA analysis as well as *via* the similarity of their ATR-FTIR spectra with that of Y^ttt^ (Fig. S5 and S6†). The isolation of the desired Nd(iii) derivative proved less straightforward as recrystallization from pentane afforded a mixture of visually discernible blue and turquoise crystals, necessitating additional purification *via* high vacuum sublimation under elevated temperature (10^−5^ mbar, 120–140 °C). Based on the EA analysis of the two crystalline materials the following bulk composition could be established: (COT)Nd(Cp^ttt^) (Nd^ttt^, blue) and its THF adduct (COT)Nd(Cp^ttt^)(THF) (Nd^ttt^·THF, turquoise). FTIR analysis bolsters this assignment with the spectrum of Nd^ttt^ being analogous to those obtained for late lanthanide derivatives (Fig. S7†) while the Nd^ttt^·THF spectrum exhibits additional features between 850 and 1100 cm^−1^ that can be ascribed to a metal-bound THF (Fig. S8†), in line with the literature reports on (COT)La(Cp*)(THF).^24^ Crucially, a similar synthetic approach (utilizing KCp* instead of KCp^ttt^) did not lead to the formation of the monomeric (COT)Nd(Cp*). Instead, a complex tetrameric derivative featuring residual triflate moieties and potassium ions was formed (Fig. S15†). While the resulting species is not polymeric as in the case of La(iii), it is clear that the monomeric Nd(iii) congener remains inaccessible with this less bulky ligand set.

### Analysis of the solid-state structure

**Fig. 1:**
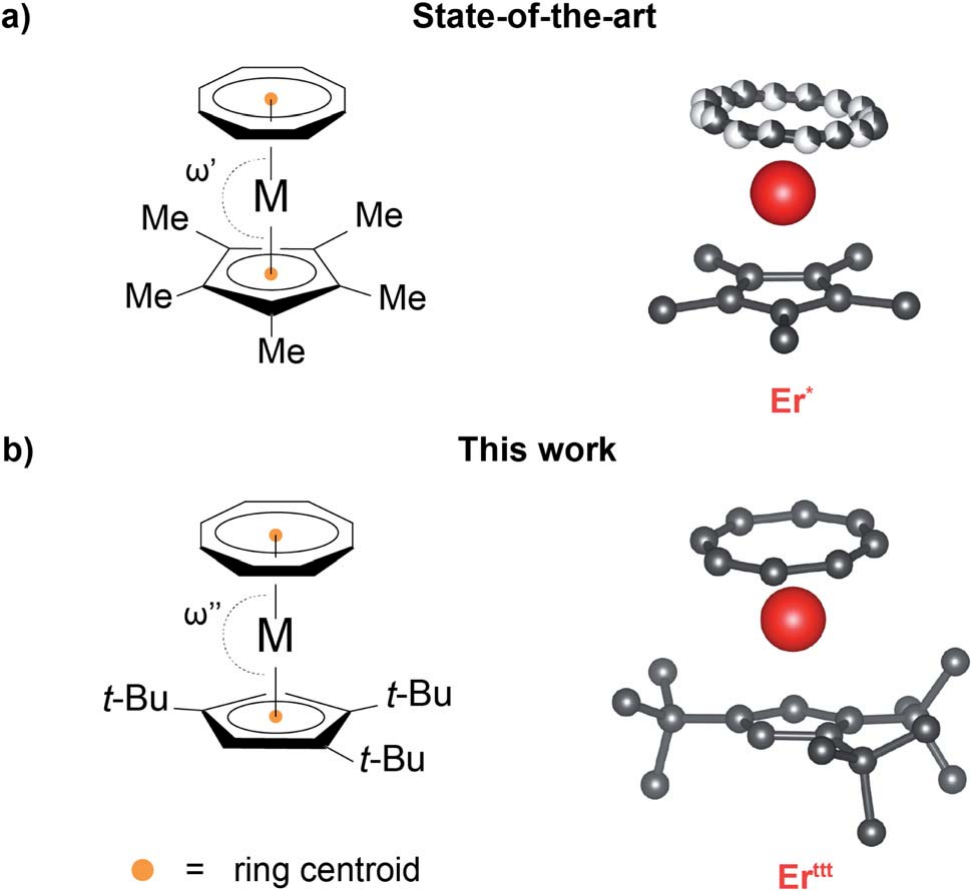
Schematic representations and the representative crystal structures of Er(iii) derivatives of (a) (COT)M(Cp*) and (b) (COT)M(Cp^ttt^) compound families. The disorder of the (COT)^2−^ ligand is visualized; hydrogen atoms are omitted for clarity.

Recrystallization of the obtained compounds afforded crystals suitable for single crystal X-ray diffraction (SCXRD) characterization. Y^ttt^, Dy^ttt^ and Er^ttt^ (Fig. 1b and S9–S11 as well as Table S1†) are all isomorphous and crystallize in monoclinic *P*2_1_/*n* space group with almost identical unit cell metrics. As in the case of the Cp* derivatives, two distinct molecular orientations that are approximately perpendicular to each other (evaluated based on the planes drawn through Cp^ttt^ rings) coexist within the unit cell. While potentially a complicating factor in the context of magnetic properties determination, extensive angular-resolved magnetometry studies^33^ performed on (COT)Er(Cp*) (Er*) demonstrated that both orientations exhibit the same inherent molecular magnetic behavior. One of the most striking differences between the crystal structures of Er^ttt^ and Er* is the lack of static (COT)^2−^ disorder that persists over a wide range of temperatures (10–298 K, see Fig. 1). Inspection of the distances (for a list and associated uncertainties see Table S3†) between Er(iii) and its immediate neighbors in Er^ttt^ revealed that the average Er–C_Cp_ distance equals 2.608 Å, while the distance between the metal center and the calculated centroid of the (Cp^ttt^)^−^ is 2.310 Å. These distances are slightly longer than in the case of Er*. Conversely, the average Er–C (2.519 Å) and Er-calculated ring centroid (1.718 Å) distances for (COT)^2−^ are marginally shorter than in the case of the less bulky Er* derivative. The most prominent difference between the (Cp^ttt^)^−^ and (Cp*)^−^ derivatives in terms of crystal structure metrics are the centroid_COT_–Er–centroid_Cp_ (bending angle, *ω*) angles which equal 175.93° for Er^ttt^ and 171.08° (average of two rotamers) for Er*. In the case of Er* such bent geometry results from intermolecular interactions within the crystal lattice.^15^ While the *ω* angle in Er^ttt^ is much closer to the idealized parallel orientation of both rings (*ω* = 180°), it is still slightly bent. To determine whether this effect is of a molecular origin or driven by crystal packing we assembled a single molecule of Er^ttt^*in silico* and optimized its geometry in vacuum (Fig. S12†). The obtained model is bent despite the lack of contributions from the neighboring molecules with *ω* = 175.10°, a value that closely reproduces the experimental SCXRD data. In contrast, an analogous computational protocol applied to Er* yielded a model in which the (Cp*)^−^ and (COT)^2−^ rings are almost parallel (*ω* = 179.43°). In light of this result, we posit that for Er^ttt^ the slight bending arises from the intramolecular interactions rather than from crystal packing effects. Taken together these data indicate that switching to the bulkier (Cp^ttt^)^−^ ligand allowed us to overcome the majority of solid-state structure complexities that plague the Er*. Similar trends in bond metrics can be observed when Y^ttt^ and Dy^ttt^ are compared with their respective (Cp*)^−^ analogs.

The analysis of the solid-state structures of early lanthanide-based derivatives (Fig. S13 and S14† as well as Table S2†) revealed that Nd^ttt^ crystallizes in monoclinic P2_1_/*n* (paralleling the late lanthanide derivatives) and Nd^ttt^·THF in orthorhombic *Pbca* space groups. The average Nd–C_Cp_ bond length for Nd^ttt^ is equal to 2.726 Å with the distance from Nd to the ring centroid equal to 2.443 Å. The analogous metrics in Nd^ttt^·THF are elongated, yielding an average Nd–C_Cp_ bond length of 2.782 Å and Nd–centroid_Cp_ distance of 2.505 Å. Similar to the other rare-earth metal derivatives, the (COT)^2−^ ring is much closer to the metal center compared to (Cp^ttt^)^−^, with Nd–centroid_COT_ distances of 1.895 Å (average Nd–C_COT_ bond lengths of 2.640 Å) for Nd^ttt^ and 1.965 Å (average Nd–C_COT_ bond lengths of 2.689 Å) for Nd^ttt^·THF. While we were able to obtain the desired Nd^ttt^, the concomitant formation of Nd^ttt^·THF demonstrates that the metal center is still accessible despite usage of the bulkier ligand scaffold. Furthermore, the coordination sphere around Nd(iii) appears to be more flexible with the bending angle *ω* varying from 173.07° for Nd^ttt^ to 147.45° for Nd^ttt^·THF.

### Static magnetic properties

**Fig. 2:**
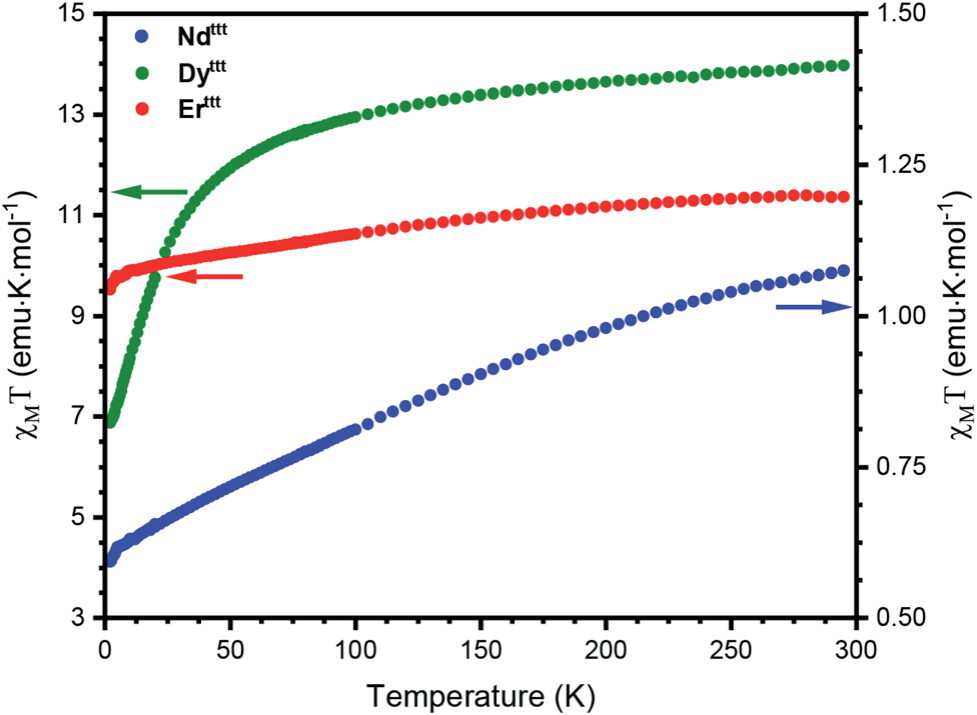
Temperature dependence of *χ*_M_*T* for Er^ttt^, Dy^ttt^ and Nd^ttt^ measured in the 2–300 K range. *χ*_M_*T* values for Er^ttt^ and Dy^ttt^ were scaled by factors of 1.33 and 1.45 to correct for the errors associated with a small sample mass.

With a representative set of species in hand we explored their static magnetic properties. First, we investigated the temperature dependence of the magnetic molar susceptibility (*χ*_M_) (Fig. 2, S17 and S18). The room temperature *χ*_M_*T* values are 11.3, 13.9 and 1.1 emu K mol^−1^ for Er^ttt^, Dy^ttt^ and Nd^ttt^, respectively. Upon lowering of the temperature a downturn can be observed in all curves with much more pronounced decrease in the case of Dy^ttt^. We have also evaluated the dependence of magnetization in response to the applied field (0–50 kOe) at 2 K (Fig. S19–S21†). Under these conditions the magnetizations of Er^ttt^ and Nd^ttt^ saturate at 4.8 and 1.0 Nβ, respectively. Conversely, the magnetization of Dy^ttt^ keeps increasing and does not saturate at 50 kOe with the highest measured value of 5.4 Nβ.

**Fig. 3:**
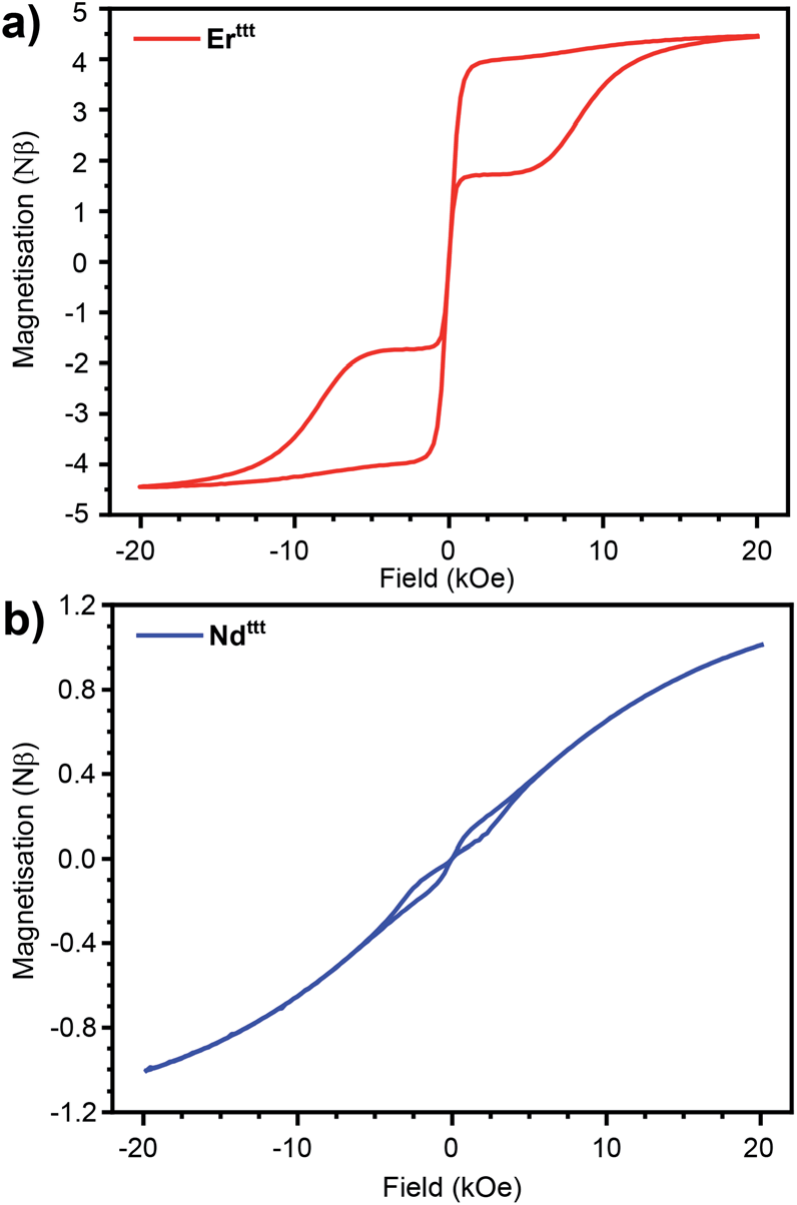
Hysteresis curves recorded at 2 K for (a) Er^ttt^ (rescaled by 1.33) and (b) Nd^ttt^ with 16 Oe s^−1^ field sweep rate.

To gauge if the prepared complexes behave as SIMs we measured the hysteresis loops at 2 K with 16 Oe s^−1^ scan speed in the −20 to +20 kOe range of the applied field (Fig. 3 and S22†). As in the case of the previously described Er*, the hysteresis loop for the novel Er^ttt^ has a butterfly shape (closing at zero-field results from QTM that is prevalent in lanthanide-based complexes). With the readily available isomorphous and diamagnetic Y^ttt^ we attempted co-crystallization of these two species to obtain magnetically dilute Er/Y^ttt^ (1.98 wt% Er). Such approach has been used before to suppress relaxation pathways stemming from the proximity of paramagnetic centers such as QTM and allows to elucidate true single-molecule magnetic properties.^13,34,35^ Accordingly, the hysteresis curve for Er/Y^ttt^ (Fig. S24†) obtained under same conditions remains open, also at zero applied field with the remnant magnetization of 1.5 Nβ, and the coercive field of ≈200 Oe. Similarly to the Er(iii) derivative, non-diluted Nd^ttt^ also exhibits a butterfly-shaped hysteresis loop that indicates SIM properties (Fig. 3b). Here, the magnetic dilution (Nd/Y^ttt^, 1.62 wt% Nd) did not lead to opening of hysteresis near zero field (Fig. S25†). In contrast to Er(iii) and Nd(iii) derivatives the non-diluted Dy^ttt^ does not show magnetic remanence (Fig. S22†). While slight opening of the curve can be achieved by the means of magnetic dilution, the hysteresis loop for Dy/Y^ttt^ (2.14 wt% Dy) remains closed in zero field further demonstrating the poor SIM performance of this oblate ion-based derivative (Fig. S23 and S27–S29†). Thus, further discussion of the magnetic properties will focus on Er^ttt^ and Nd^ttt^.

### Dynamic magnetic properties of Er^ttt^

**Fig. 4:**
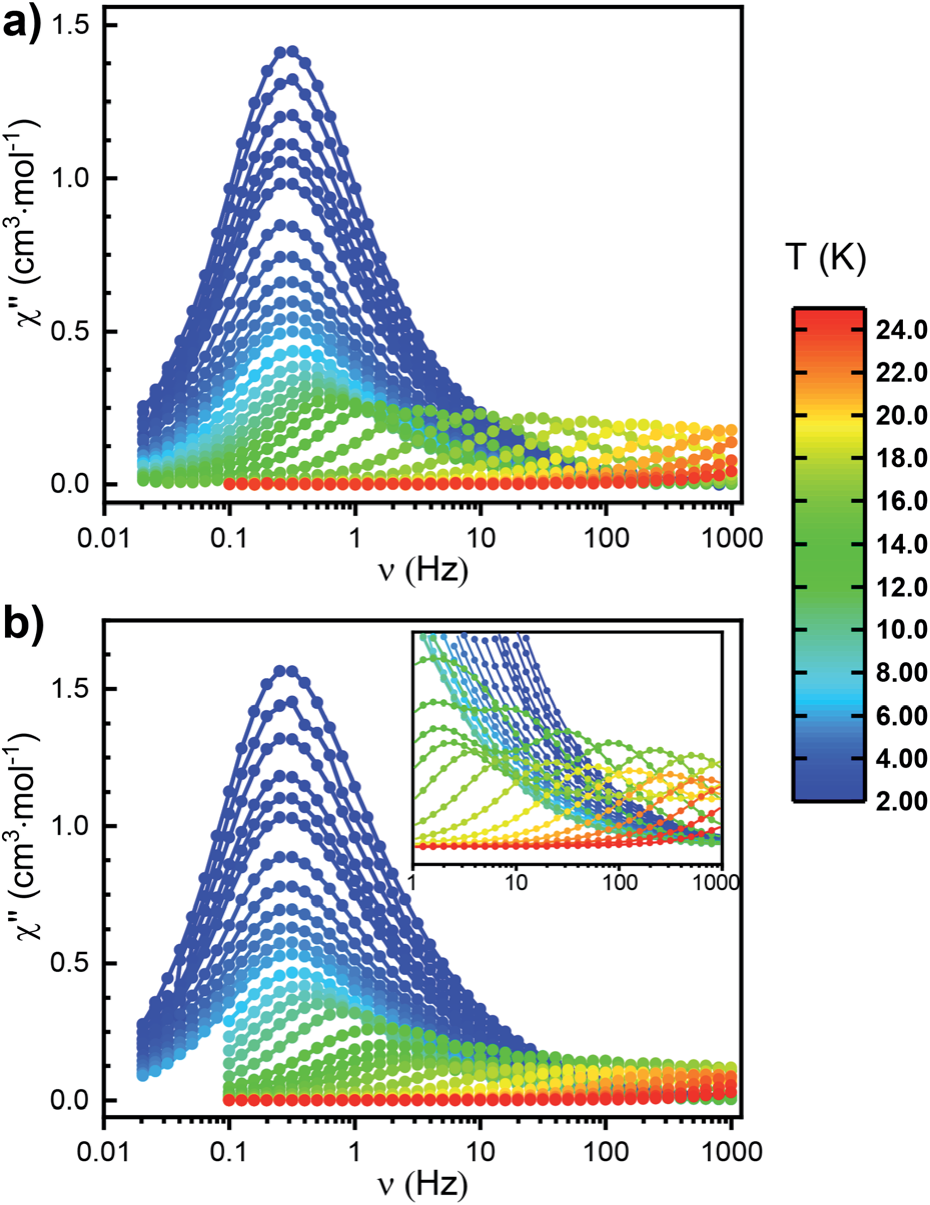
Frequency dependence of the out-of-phase component of the AC susceptibility measured in zero external DC field between 2 and 24 K for (a) Er^ttt^ and (b) Er*. Circles represent experimental data points; the lines do not have any physical meaning.

To gain more insight into the relaxation characteristics of the prepared compounds we turned to alternating current (AC) measurements. The *χ*′′(*ν*) (where *χ*′′ and *ν* denote out-of-phase magnetic susceptibility component and oscillating field frequency, respectively) curves measured for Er^ttt^ in the 2–24 K temperature range with no external DC field exhibit maxima consistent with SIM character (Fig. 4a and S30†). Between 8 and 24 K the *χ*′′(*ν*) plots show strong temperature dependence, while below 8 K the position of the maxima remains fixed and independent from temperature. Such behavior typically indicates that QTM becomes the dominant relaxation mechanism at low temperatures. Measurements in 1000 Oe applied DC field suppress the QTM relaxation (Fig. S32 and S33†). Accordingly, *χ*′′(*ν*) curves under the applied static field show clear temperature dependence in the entire available measurement window. Similar effects could also be observed during AC measurements performed on the magnetically dilute Er/Y^ttt^ sample (Fig. S36, S38 and S39†). The most striking observation (with and without the applied field as well as in the case of magnetic dilution) is the presence of only one maximum in the *χ*′′(*ν*) at each temperature in the thermally activated region. This stands in stark contrast with Er* for which two relaxation events could be identified in AC measurements (Fig. 4b). Previous studies correlated the presence of multiple maxima with the solid-state disorder of the (COT)^2−^ ring,^14^ but little experimental evidence for this hypothesis has been shown to date. With its lack of structural disorder and simultaneous presence of singular relaxation event, Er^ttt^ clearly demonstrates the validity of such claim.

Excited by the prospects of Er^ttt^ to act as a structurally and magnetically less complex (thus more easily understood) representative of the (COT)M(Cp) architecture, we performed a quantitative analysis of its relaxation behavior. In-phase susceptibilities (*χ*′) as well as *χ*′′ obtained in the AC measurements (in the 2–22 K temperature range) were fitted using the extended Debye model to obtain the specific relaxation times (*τ*) at each temperature (Fig. S31 and Table S5†). The obtained *α*_max_ (highest value of the parameter describing the distribution of relaxation times about the *τ*) equal to 0.17 indicates a narrow distribution consistent with the molecular character of the described SIM. With the so-obtained *τ* values, we were able to model the relaxation behavior by taking into account the Orbach (over the *U*_eff_ barrier), Raman and QTM processes (summarized in Tables 1 and S11†). To improve the quality of this procedure, a simultaneous fit was performed on the data obtained both under 0 and 1000 Oe of the applied static field (Fig. S35†). The resulting *U*_eff_ value (see Table S11† for the associated uncertainties) equals to 228 cm^−1^, which is almost exactly the same as the one attributed to the slower-relaxing conformer of Er* (224 cm^−1^). A similar *U*_eff_ value (235 cm^−1^) was also obtained when the fitting was performed on AC data collected for Er/Y^ttt^ (Fig. S41†). To confirm that the analogy in the magnetic behaviors of Er^ttt^ and Er* extends also to the QTM region, we performed the measurements for the latter in the relevant frequency range (0.1–1 Hz) that was not investigated in the original report. Interestingly, these measurements revealed an analogous QTM behavior as the one observed for Er^ttt^ (Fig. 4b). As a final gauge of similarity between the two derivatives we performed CASSCF/RASSI-SO/SINGLE_ANISO computations using the solid-state structure obtained from SCXRD measurements as an input (Table S13 as well as Fig. S50 and S51†). These computations revealed a highly axial ground state with 99.7% contribution of the |±15/2〉 *m*_J_ state that is well separated from the first excited state by 116.4 cm^−1^. This energy landscape correlates well with computations performed on the better performing rotamer of Er*. Most importantly, the computed probabilities for various relaxation processes are very similar for both derivatives.^33^ Consequently, Er^ttt^ can be viewed as an ideal representative of the (COT)M(Cp) architecture with its more accessible magnetic behavior and quantitatively similar relaxation characteristics as the better-performing rotamer of the iconic Er*.

**Table 1:** Parameters for the relaxation processes for Er^ttt^ and Nd/Y^ttt^ obtained from fitting of AC measurement data using 
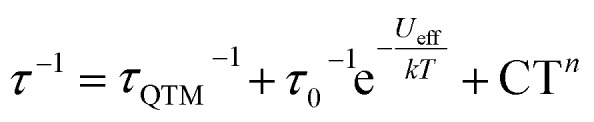
 equation, with the three terms describing quantum tunneling of magnetization, Orbach, and Raman relaxation processes, respectively

	Field [Oe]	*τ*_QTM_ [s]	*τ*_0_ [s]	*U*_eff_ [cm^−1^]	*C* [s^−1^ K^−*n*^]	*n*
Er^ttt^	0	4.31 × 10^−1^	2.74 × 10^−11^	228.26	5.44 × 10^−7^	5.89
	1000	—				
Nd/Y^ttt^	0	6.73 × 10^−2^	1.16 × 10^−6^	24.09	—	—
	1000	—				

### Analysis of Er(iii) intra-4f optical transitions

**Fig. 5:**
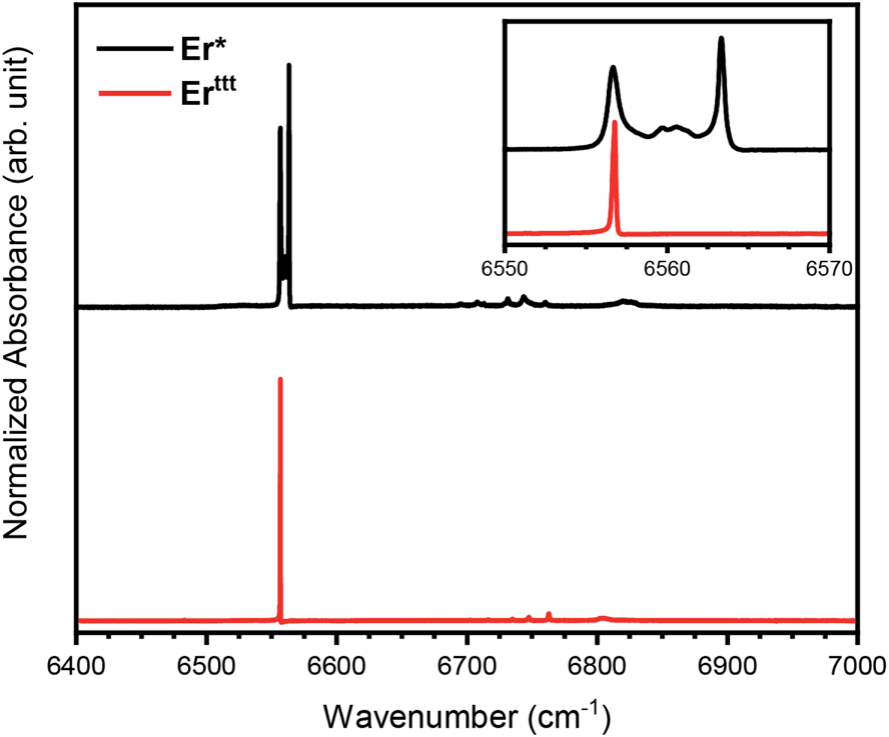
FTIR absorption spectra of Er* and Er^ttt^ SIMs at 3 K, corresponding to the energy range of the intra-4f transitions from the lowest doublet of the ^4^I_15/2_ manifold to the doublets of the excited manifold ^4^I_13/2_. The inset shows a zoom on the sharpest transition lines.

To further understand the differences between the Er* and Er^ttt^ we have performed FTIR spectroscopy measurements in the energy region of 6400–7000 cm^−1^. The absorbance spectrum at 3 K (Fig. 5) reveals only one transition line around 6550 cm^−1^ for Er^ttt^, while in the case of Er* a more complex structure is present, with the absorbance mainly given by two sharp peaks, distant 6.6 cm^−1^ apart. In the case of Er^ttt^ we attribute the large peak visible in the inset of Fig. 5 to one of the transitions from the eight Kramers doublets of the ground state manifold ^4^I_15/2_ to the first excited state manifold ^4^I_13/2_ that consists of seven doublets.^36,37^ However, by keeping the samples at very low temperature (3 K) only the lowest doublet of the ground state manifold, *m*_J_ = 15/2, is populated. As previously mentioned, the rather complex behavior of the Er* compound can be explained in terms of a slightly different ligand field experienced by the Er(iii) ion at low temperature, since several possible rotamers can coexist. Specifically, we ascribe the presence of two main peaks in the spectrum of Er* to the presence of the two rotamers discussed earlier. As reported by the spectra in Fig. 5, the presence of additional transition lines which are split by just a few cm^−1^ suggests the presence of other metastable rotameric states beyond those identified in SCXRD studies given by local energy minima associated to different positions of the (COT)^2−^ ring with respect to the (Cp*)^−^ ring and, eventually, the methyl groups. The ratio between the estimated areas of the two main peaks in the Er* is approximately 59 : 41, which is close to the ratio of the two rotamers suggested in the literature (62 : 38).^14^ Additional small peaks visible in Fig. 5 at around 6750 cm^−1^ are attributed to transitions to other doublets of the ^4^I_13/2_ manifold, which are weakly allowed due to mixing of the *m*_J_ substates. The fact that only one sharp absorption line with a FWHM of only 0.2 cm^−1^ is detected for the Er^ttt^ molecules is perfectly in line with the structural simplicity and dynamic magnetic properties of this compound.

### Dynamic magnetic properties for Nd^ttt^

**Fig. 6:**
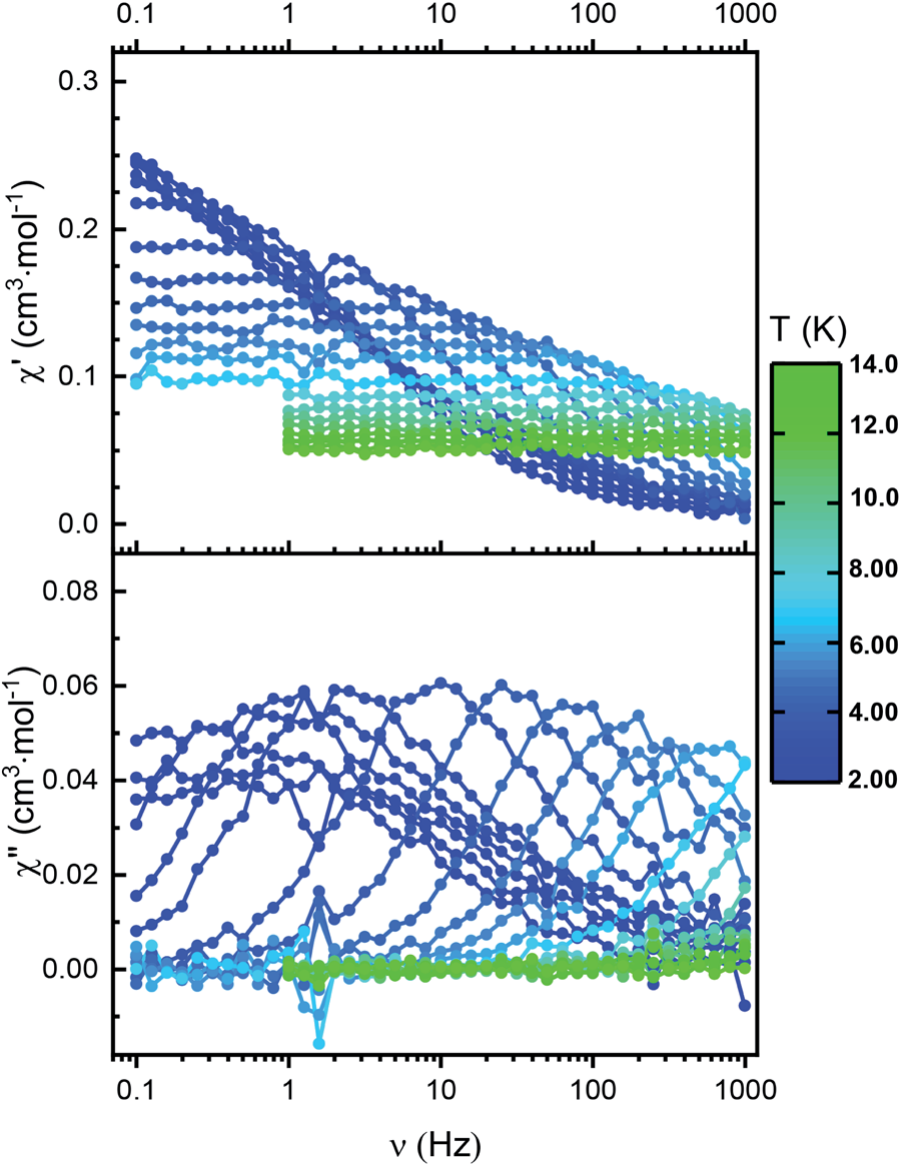
Frequency dependence of both in-phase (top) and out-of-phase (down) components of the AC susceptibility measured in zero external DC field between 2 and 14 K for Nd/Y^ttt^. Circles represent experimental data points; the lines do not have any physical meaning.

The *χ*′′(*ν*) curve at 2 K for Nd^ttt^ (Fig. S42†) increases with the frequency of the oscillating field without reaching a maximum in the available measurement window (0.1–1000 Hz) indicating faster relaxation characteristics in zero field than Er^ttt^. However, upon applying of a static DC field of 1000 Oe, the maxima in *χ*′′(*ν*) curves begin to emerge within the measured frequency window. Relaxation *via* QTM can be an intrinsic phenomenon of the molecule or can be induced by the presence of neighboring magnetic sites.^38^ To minimize such dipolar interactions we performed similar measurements for Nd/Y^ttt^, where the presence of diamagnetic matrix should lead to distancing of the paramagnetic centers. Here, the *χ*′′(*ν*) curves have clearly observable maxima that show temperature dependent behavior over the whole measured range, proving the zero-field SIM character and underlining the importance of dipolar-mediated relaxation processes in the non-diluted sample (Fig. 6). Fitting the in-phase and out-of-phase susceptibility data to a generalized Debye model (Fig. S43 and Table S9†) reveals a wide distribution of relaxation times as evidenced by *α*_max_ equal to 0.43. With an applied field of 1000 Oe, a much narrower distribution of relaxation times can be observed with *α*_max_ equal to 0.06 (Fig. S46 and Table S10†). Such variability of the *α* parameter has been observed before for Nd(iii)-based SIMs.^26,27^ Such relaxation behavior could not be reproduced solely by Orbach process (both with and without applied field). Instead, accounting for both Orbach and QTM relaxation processes is necessary to obtain a reasonable fit for the experimental data (Tables 1 and S11 as well as Fig. S47†).

To gain a deeper understanding of the magneto-structural correlations in the investigated system we performed *ab initio* calculations. Utilization of the well-established CASSCF/RASSI-SO/SINGLE_ANISO approach leads to an overestimation of the energy splitting between the Kramers doublets and the associated *U*_eff_ for early lanthanide complexes, in line with previous reports.^26,27,39^ To mitigate these issues, a second order perturbation theory approach (CASPT2/RASSI-SO/SINGLE_ANISO) was used to include dynamic correlations (Table S14 as well as Fig. S52 and S53†). With this protocol the obtained experimental *U*_eff_ is only three times smaller than the calculated gap between the ground and the first excited state (78.2 cm^−1^) (for more details see the ESI file†). The calculated ground state within the ^4^I_9/2_ term of Nd(iii) is anisotropic and highlights a pronounced axial character (*g*_*x*_ ∼ 0.2, *g*_*y*_ ∼ 0.7, *g*_*z*_ ∼ 4.6). The analysis of the wave function composition reveals the mixed nature of the ground state, which is in line with the experimental low temperature values of the *χ*_M_*T* measurement. Further, the calculated transition moments suggest a fast relaxation *via* the first excited state as indicated by prominent transverse magnetic components between the Kramers doublets. In addition, QTM in the ground state is non-negligible.

## Conclusions

In this work, we introduced a family of complexes representing the (COT)M(Cp) architecture. By moving from the (Cp*)^−^ to the (Cp^ttt^)^−^ ligand we were able to prepare an Er(iii) derivative that lacks the structural complexity of the less bulky congener. This makes (COT)Er(Cp^ttt^) an attractive model compound for further magnetic studies of this architecture, *e.g.*, its surface deposition.^40^ In addition, incorporation of the (Cp^ttt^)^−^ ligand allowed us to prepare the first early-lanthanide monomeric representative of the (COT)M(Cp) architecture. The magnetic studies revealed that it acts as a zero-field SIM with one of the highest *U*_eff_ barriers for relaxation of magnetization reported to date for early lanthanide SIMs.

## Data availability

The datasets supporting this article have been uploaded as part of the supplementary material. Crystallographic data for Y^ttt^ (2170514), Nd^ttt^ (2170515), Er^ttt^ (2170516), K_2_[(COT)Nd(Cp*)]_4_(OTf)_2_ (2170517), Dy^ttt^ (2170518) and Nd^ttt^·THF (2170525) have been deposited at the Cambridge Crystallographic Data Center (CCDC).

## Author contributions

M. D. K. and C. C. conceptualized the project. M. D. K. and M. B. performed the geometry optimizations, designed and carried out the syntheses of the described materials, analyzed the relevant diffraction data and carried out the spectroscopic characterization. M. B. and B. L. G. completed the *ab initio* calculations and processed the results. O. C. and M. B. performed SQUID measurements and analyzed the data. V. R., G. M. and J. D. collected and analyzed the FTIR data pertaining to Er intra-4f optical transitions. F. P. and C. C. acquired funding for the project. All authors were involved in writing of the manuscript and approved its final version.

## Conflicts of interest

There are no conflicts to declare.

## Supplementary Material

SC-013-D2SC02560D-s001

SC-013-D2SC02560D-s002
