# Supplementary material for: Cyclooctatetraenide-based single-ion magnets featuring bulky cyclopentadienyl ligand
Source: Chem Sci. 2022 Aug 30;13(35):10574–80. doi: 10.1039/d2sc02560d (PMC9473503; doi:10.1039/d2sc02560d)
Supplement: SC-013-D2SC02560D-s001 [file SC-013-D2SC02560D-s001.pdf]

Electronic Supplementary Material (ESI) for Chemical Science.
This journal is © The Royal Society of Chemistry 2022

Supplementary Information for: Cyclooctatetraenide-Based Single-Ion Magnets Featuring Bulky Cyclopentadienyl Ligand

Maciej D. Korzyński*[,a], Moritz Bernhardt[a], Vladyslav Romankov[b], Jan Dreiser[b], Guy Matmon[b], Fabrice Pointillart[c], Boris Le Guennic*[,c], Olivier Cador*[,c], and Christophe Copéret*[,a]

[a]Department of Chemistry and Applied Biosciences, ETH Zürich, Vladimir-Prelog Weg 1-5/10, 8093 Zürich, Switzerland

[b]Swiss Light Source, Paul Scherrer Institut, 5232 Villigen PSI, Switzerland

[c]Univ Rennes, CNRS, ISCR (Institut des Sciences Chimiques de Rennes), UMR 6226, 35000 Rennes, France

*korzynski@inorg.chem.ethz.ch, boris.leguennic@univ-rennes1.fr, olivier.cador@univ-rennes1.fr, ccoperet@ethz.ch

General information

Potassium (99.5% trace metals basis), trifluoromethanesulfonic acid (reagent grade, 98%), dicyclopentadiene, 2-bromo-2-methylpropane (for synthesis), sodium hydride (60% dispersion in mineral oil), potassium bis(trimethylsilyl)amide (95%), yttrium(III) chloride hexahydrate (99.99% trace metals basis), diethyl ether (ACS Reag., ≥99.8%) and pentane (HPLC, ≥99.0%) were obtained from Sigma Aldrich. Yttrium(III) oxide (99.99%), dysprosium(III) oxide (99.99%) and 1,3,5,7-cyclooctatetraene (98%) were obtained from ABCR. Neodymium oxide (99.9%) and erbium oxide (99.9.%) were purchased from LTS Research Laboratories, Inc. Eicosane (99%) was purchased from Acros Organics. Tetrahydrofuran (LiChrosolv® for liquid chromatography) was obtained from Merck. Toluene (≥99.8%) was purchased from Fisher Chemical. 18-crown-6 (purum, ≥99.0%) was obtained from Fluka Chemie. Benzene-*d6* (99.5% D) was purchased from Eurisotop. Ultrapure water (18.2 MΩ·cm) was obtained using Synergy® purification system. Toluene, diethyl ether, and pentane were dried using the commercial MBraun SPS-800 solvent purification system, degassed via three freeze-pump-thaw cycles, and stored over 4Å molecular sieves (MS) under argon atmosphere. Tetrahydrofuran and benzene-*d6* were distilled from sodium in presence of benzophenone and stored over 4Å MS under argon atmosphere. 1,3,5,7-Cyclooctatetraene was dried over 4Å MS, vacuum transferred, degassed via three freeze-pump-thaw cycles, and stored under argon atmosphere. 1,3,5-Tri(*tert*-butyl)cyclopentadiene[1] ($Cp^{ttt}H$), its potassium salt[2] (KCp^{ttt}), yttrium, neodymium, dysprosium and erbium triflates[3] ($M(OTf)_3$ where M = Y, Nd, Dy or Er, respectively) and (COT)M(OTf) tetrahydrofuran adducts[4] (where M = Y, Nd, Dy or Er, respectively) were prepared according to the literature procedures. Unless stated otherwise, all manipulations were performed using standard Schlenk techniques or in an MBraun gloveboxes with argon as an inert gas (<0.1 ppm O_2 and H_2O). Elemental analyses were performed by Mikroanalytisches Labor Pascher, Remagen, Germany. All structural models were visualized using VESTA 3.5.7.[5]

All of the liquid state nuclear magnetic resonance (NMR) spectra were recorded on a Bruker 500 MHz (11.7 T) NMR spectrometer. The ^{1}H and ^{13}C chemical shifts were calibrated to the residual signals from the deutered solvent.[6] In acquisition of ^{1}H spectra 1 s relaxation delay was used. ^{89}Y spectra were recorded using a Bruker anti-ringdown (aring) pulse sequence with 5 s interscan relaxation. The liquid-state ^{89}Y NMR spectra were referenced to an aqueous solution of $YCl_3 \cdot 6\ H_2O$ at 0 ppm.

Attenuated total reflectance Fourier-transform infrared spectroscopy (ATR-FTIR) spectra were collected using a Bruker ALPHA spectrometer equipped with MIR source, KBr beamsplitter, RT-DLaTGS detector and Platinum ATR accessory housed in argon-filled glovebox. The data were collected between 4000 and 400 cm^{-1} with 4 cm^{-1} averaging

over 24 scans. The transmission FTIR experiments aimed at probing the f-f optical transitions were performed at the IR beamline of the Swiss Light Source at the Paul Scherrer Institut, Switzerland, which is equipped with a Bruker IFS125 HR spectrometer with an optical path difference of up to 12.6 m. A CaF_2 beam splitter and a liquid nitrogen-cooled InSb detector were used to access the energy range of $\approx$ 6000 - 7000 cm^{-1}. The spectral resolution was set to 0.05 cm^{-1} after verifying that the observed linewidths are not instrument limited by performing test scans with a resolution of 0.005 cm^{-1}. Polycrystalline solid samples were finely ground and pressed into pellets of 0.3 mm thickness with a previously outgassed commercial KBr powder (Sigma Aldrich). The samples were prepared in inert He gas environment and transferred into a continuous-flow He cryostat equipped with NIR grade quartz windows. The heatsink base was kept at low temperature (3 K).

Single crystal X-Ray diffraction (SCXRD) data were collected using either a Bruker D8 Advance diffractometer equipped with APEX II CCD detector or an Oxford Diffraction Excalibur S diffractometer equipped with Sapphire 3 CCD detector, both utilizing a Mo K_α source (λ = 0.71073 Å), and performing φ- and ω-scans. The air-sensitive single crystals were isolated in the glovebox and covered with oil to prevent the decomposition. The crystals were promptly mounted on the goniometer and placed in a stream of cold dinitrogen to cool the sample down (100 K) and to prevent decomposition. The structures were solved using either the direct methods implemented in SHELXS[7] or the intrinsic phasing method implemented in SHELXT[8] and refined by least-squares approach using SHELXL 2018/3,,[9] all interfaced via Olex 2 software.[10] All non-hydrogen atoms were refined anisotropically. For all $\mathbf{M^{ttt}}$ and $\mathbf{Nd^{ttt}{\cdot}THF}$ hydrogen atoms were located in the difference Fourier map and refined isotropically. For each aromatic ring a FLAT instruction was applied and C–C (and in some cases aromatic C–H) distances were restrained with SADI instructions. Rigid body restraints (DELU instruction) were applied to carbon atoms constituting each aromatic ring. For $K_2[(COT)Nd(Cp^*)]_4(OTf)_2$ the hydrogen atoms that could not be located in the difference maps were placed in calculated positions using AFIX 137 and 43 instructions for methyl group and aromatic protons, respectively, and refined using the riding model. The C–C distances in the non-disordered $(COT)^{2-}$ rings were fixed using the DFIX instruction to 1.41 Å, a value based on the other structures featuring this ligand. A combination of DELU, RIGU, ISOR and EADP instructions was applied to refine the disorder present within the crystal structure.

The static susceptibility measurements were performed on solid polycrystalline samples with a Quantum Design MPMS-XL SQUID magnetometer. The following values of magnetic field were used 0.2 kOe, 2 kOe and 10 kOe for the temperature ranges of 2-20 K, 20-80 K and 80-300 K, respectively, in order to prevent any saturation effect in the case of $\mathbf{Er^{ttt}}$ and $\mathbf{Dy^{ttt}}$; in the case of $\mathbf{Nd^{ttt}}$ 2 kOe and 10 kOe were used for the temperature

ranges of 2-20 K and 20-300 K, respectively. The AC magnetic susceptibility measurements were performed on Quantum Design MPMS-XL SQUID magnetometer (0.01-1000 Hz frequency range). Owing to the extreme air sensitivity of the materials, for all of the measurements the samples were sealed in quartz EPR tubes. All materials were mixed with eicosane. Each mixture was placed at the bottom of an EPR tube and sealed under high vacuum. The bottom of the tube was warmed at 40° C to melt eicosane, which upon cooling formed a solid matrix adhering to the walls of the tube. The measurements were all corrected for the intrinsic diamagnetic contribution as calculated with Pascal's constants[11] and also for the diamagnetic contribution of the eicosane.

Synthetic procedures

Preparation of Y^{ttt}. $[(COT)Y(OTf)(THF)]_2$ (423.0 mg, 0.51 mmol) was dissolved in 11 mL of THF and the resulting yellow mixture was cooled down to 0 °C. Solid KCp^{ttt} (278.9 mg, 1.02 mmol) was added in small portions into the vial with vigorous stirring over 10 minutes. The mixture was kept cold for 20 minutes and subsequently warmed up to room temperature. After 24 hours the yellow reaction mixture was filtered and solvent was removed *in vacuo* to afford off-white solids. These solids were extracted with 3 x 5 mL of pentane and the mixture was filtered again. Following the solvent removal the resulting crude product was recrystallized from pentane (–30 °C) to afford the desired product as off-white crystals (136.3 mg, 31.2 %). ^{1}H NMR (C_6D_6, 500.26 MHz): δ 6.40 (s, 8H, $C_{COT}H$), 5.55 (s, 2H, $C_{Cp}H$), 1.26 (s, 18H, 2x$C(CH_3)_3$), 1.15 (s, 9H, $C(CH_3)_3$). ^{13}C{^{1}H} NMR (C_6D_6, 125.80 MHz): δ 135.34, 133.61, 106.17, 93.60, 34.42, 33.18, 32.27, 31.66. ^{89}Y NMR (C_6D_6, 24.51 MHz): δ -149.16. Found: C, 69.25 %; H, 8.65 %. Calc. for $C_{25}H_{37}Y$: C, 70.41 %; H, 8.75 %.

Preparation of Dy^{ttt}. $[(COT)Dy(OTf)(THF)_2]_2$ (350.0 mg, 0.31 mmol) was dissolved in 9.5 mL of THF and the resulting yellow mixture was cooled down to 0 °C. Solid KCp^{ttt} (195.5 mg, 0.72 mmol) was added in small portions into the vial with vigorous stirring over 10 minutes. The mixture was kept cold for 20 minutes and subsequently warmed up to room temperature. After 24 hours the yellow reaction mixture was filtered and solvent was removed *in vacuo* to afford yellow solids. These solids were extracted with 3 x 5 mL of pentane and the mixture was filtered again. Following the solvent removal the resulting crude product was recrystallized from pentane (–30 °C) to afford the desired product as yellow crystals (105.0 mg, 33.9 %). Found: C, 59.93 %; H, 7.48 %. Calc. for $C_{25}H_{37}Dy$: C, 60.05 %; H, 7.46 %.

Preparation of Er^{ttt}. $[(COT)Er(OTf)(THF)_2]_2$ (361.2 mg, 0.32 mmol) was dissolved in 9.5 mL of THF and the resulting pink mixture was cooled down to 0 °C. Solid KCp^{ttt} (199.5 mg, 0.73 mmol) was added in small portions into the vial with vigorous

stirring over 10 minutes. The mixture was kept cold for 20 minutes and subsequently warmed up to room temperature. After 24 hours the pink reaction mixture was filtered and solvent was removed *in vacuo* to afford pink solids. These solids were extracted with 3 x 5 mL of pentane and the mixture was filtered again. Following the solvent removal the resulting crude product was recrystallized from pentane (–30 °C) to afford the desired product as pink crystals (120.0 mg, 37.1 %). Found: C, 59.53 %; H, 7.42 %. Calc. for $C_{25}H_{37}Er$: C, 59.48 %; H, 7.39 %.

Preparation of $\mathbf{Nd^{ttt}}$. $[(COT)Nd(OTf)(THF)_2]_2$ (286.0 mg, 0.26 mmol) was dissolved in 9.5 mL of THF and the resulting green mixture was cooled down to 0 °C. Solid KCp^{ttt} (166.0 mg, 0.61 mmol) was added in small portions into the vial with vigorous stirring over 10 minutes. The mixture was kept cold for 20 minutes and subsequently warmed up to room temperature. After 24 hours the green-blue reaction mixture was filtered and solvent was removed *in vacuo* to afford green-blue solids. These solids were extracted with 3 x 5 mL of pentane and the mixture was filtered again. Following the solvent removal the resulting crude product was sublimed (120-140 °C, 10^{-5} mbar) and isolated as blue solid (60 mg, 23.9 %). Crystals suitable for SCXRD analysis were grown from pentane. Found: C, 62.55 %; H, 7.80 %. Calc. for $C_{25}H_{37}Nd$: C, 62.32 %; H, 7.74 %. EA for the byproduct, $\mathbf{Nd^{ttt}{\cdot}THF}$, found: C, 61.98 %; H, 8.13 %. Calc. for $C_{29}H_{45}ONd$: C, 62.88 %; H, 8.19 %.

Preparation of magnetically diluted samples. The desired lanthanide $\mathbf{M^{ttt}}$ derivative was mixed with $\mathbf{Y^{ttt}}$ in an approximately 1:18 molar ratio. The solid mixtures were dissolved in pentane. Crystallization at –30 °C afforded the respective $\mathbf{M/Y^{ttt}}$ species. For the diluted samples, the following EA results have been obtained:
$\mathbf{Er/Y^{ttt}}$: C, 69.98 %; H, 8.62 %; Er, 1.98 %; Y, 18.9 %
$\mathbf{Dy/Y^{ttt}}$: C, 69.35 %; H, 8.57 %; Dy, 2.14 %; Y, 19.6 %
$\mathbf{Nd/Y^{ttt}}$: C, 67.14 %; H, 8.06 %; Nd, 1.62 %; Y, 19.0 %.

Computational methods

The geometry optimizations for molecular models were performed in ORCA 5.0.2 computational package[12–14] utilizing the PBE0 functional.[15] Zeroth-Order Regular Approximation (ZORA)[16–18] was used to account for the relativistic effects. ZORA-def2-TZVPP and SARC-ZORA-TZVPP basis sets were used for main group and rare-earth elements, respectively, while an additional auxiliary basis set SARC-J was applied.[19–23] A higher precision of the DFT grid was achieved by increasing the radial grid on the rare-earth metal to 6. To speed up the calculations, the 'resolution of the identity' approximation in terms of a Split-RI-J variant in combination with the 'chain-of-spheres exchange' approximation (RIJCOSX)[24] was used to generate the Coulomb and the Hartree-Fock

exchange integrals. Additionally, a faster convergence in the SCF steps was achieved by the applying KDIIS[25] and SOSCF[26] keywords. In all calculations the DFT-D3 diffusion correction with Becke-Johnson damping function (D3BJ) was applied.[27,28]

Calculation of the electronic structure and magnetic properties were performed with State-Averaged Complete Active Space Self-Consistent Field approach with Restricted Active Space State Interaction method (SA-CASSCF/RASSI-SO) included in the Open-Molcas 19.11 version.[29] The performed multi-configurational approach used the Douglas-Kroll Hamiltonian to treat the relativistic effects in two steps. In the basis-set generation scalar terms are included and are further used in the determination of the CASSCF wave-functions and energies.[30] The calculated CASSCF wavefunctions are mixed within the RASSI-SO method to account for spin-orbit coupling.[31,32] The active space was chosen to span the seven 4f orbitals populated with three, nine and eleven 4f electrons for Nd(III), Dy(III) and Er(III), respectively. In described active space, state-averaged CASSCF calculations were performed on all 21 sextets, all 224 quartets and all 490 doublets for dysprosium while for neodymium and erbium all 35 quartets and 112 doublets were computed. These excited states were mixed using the RASSI-SO module which includes all 21 sextets, 128 quartets and 130 doublets for Dy(III) and all 35 quartets and 112 doublets for Nd(III) and Er(III). The atomic natural (ANO_RCC) basis set with the following contractions was employed for the description of the different atoms: [8s7p5d4f2g1h] for Nd, Dy and Er; [4s3p2d1f] for C and [2s1p] for H.[33,34] To speed up the calculation and save disc space the Cholesky decomposition of the bielectronic integrals was employed.[35] In the final step, the magnetic properties and the g-tensors of the ground state multiplet was computed using the SINGLE_ANISO routine.[36] Based on the CASSCF wavefunctions, dynamic correlation energies for **Nd$^{\mathbf{ttt}}$** were included with second-order multiconfigurational perturbation theory (CASPT2)[37,38] to improve the computed properties for the early lanthanide complex. To speed up the calculation 49 orbitals per symmetry were frozen. The zeroth-order Hamiltonian was modified with the IPEAshift[39] and IMAGinary[40] keywords to minimize the problem of intruder states and systematic errors which lead to an overestimation of the correlation energy in open-shell systems. For the chosen active space, three electrons in seven 4f orbitals for Nd(III), 35 quartets and 40 doublets out of the 35 quartets and 112 doublets computed on the CASSCF level were considered for the CASPT2 step. In the final step, spin-orbit coupling was introduced with the RASSI module and the SINGLE_ANISO routine was used to compute the magnetic properties. To give more insights into the orientation of the magnetic axis, the molecular electrostatic potential is calculated from the *ab initio*

LOPROP charge analysis[41] (eq. 1)

$$V(r_i) = \sum_{i}^{N} \frac{q_i}{|r_i - r|} + \frac{p_i \cdot r_i}{|r_i - r|^3} + \frac{r_i \cdot (Q_i \times r_i)}{|r_i - r|^5} \qquad (1)$$

where q_i, p, Q_i and r_i are respectively the charge, dipole, quadrupole moments and displacement vector of the i-th atom. The resulting molecular electrostatic potential was mapped and represented using the home-made CAMMEL code (Calculated Molecular Multipolar Electrostatics). The potential is drawn on a sphere defined by the user around the central lanthanide ion, for a given state (ground state in this case). For a clearer representation of the potential, the intensity can be directly related to both the color (red = high potential and blue = low potential) and the height of the irregularities. This program has already been used in previous works to give some hints on the orientation of magnetization axes.[42,43] The CAMMEL code is available under GNU General Public License v3.0 and can be downloaded at https://github.com/rmarchal1/CAMMEL.

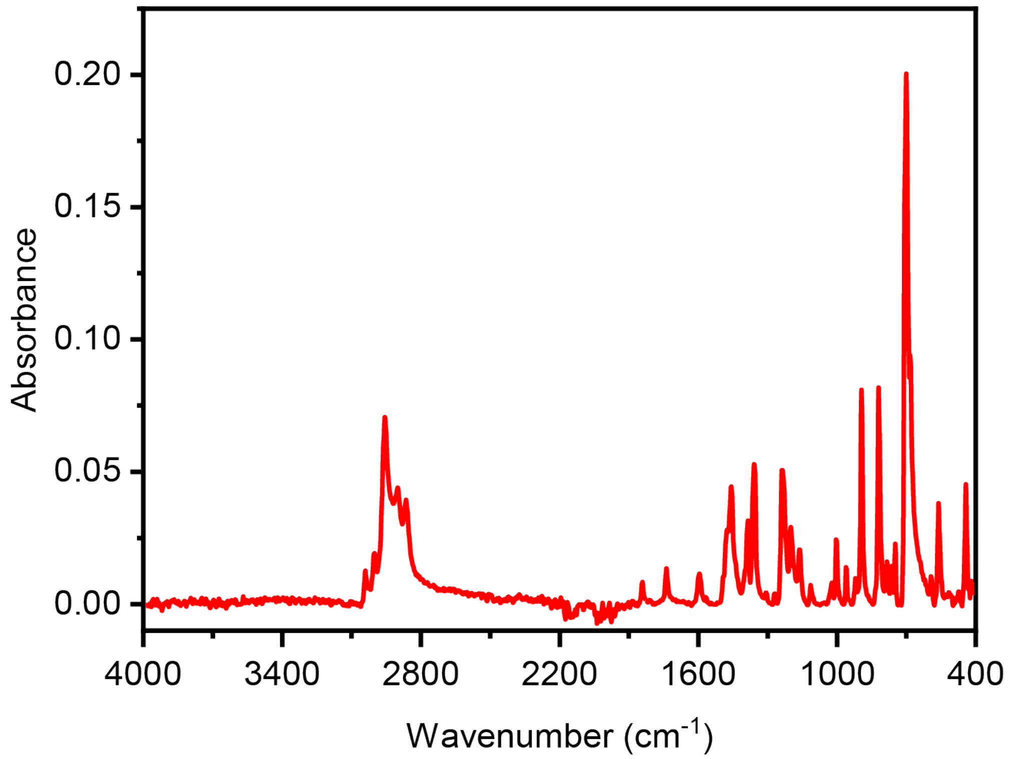

Figure S1: ATR-FTIR spectrum of $\mathbf{Y}^{\mathbf{ttt}}$.

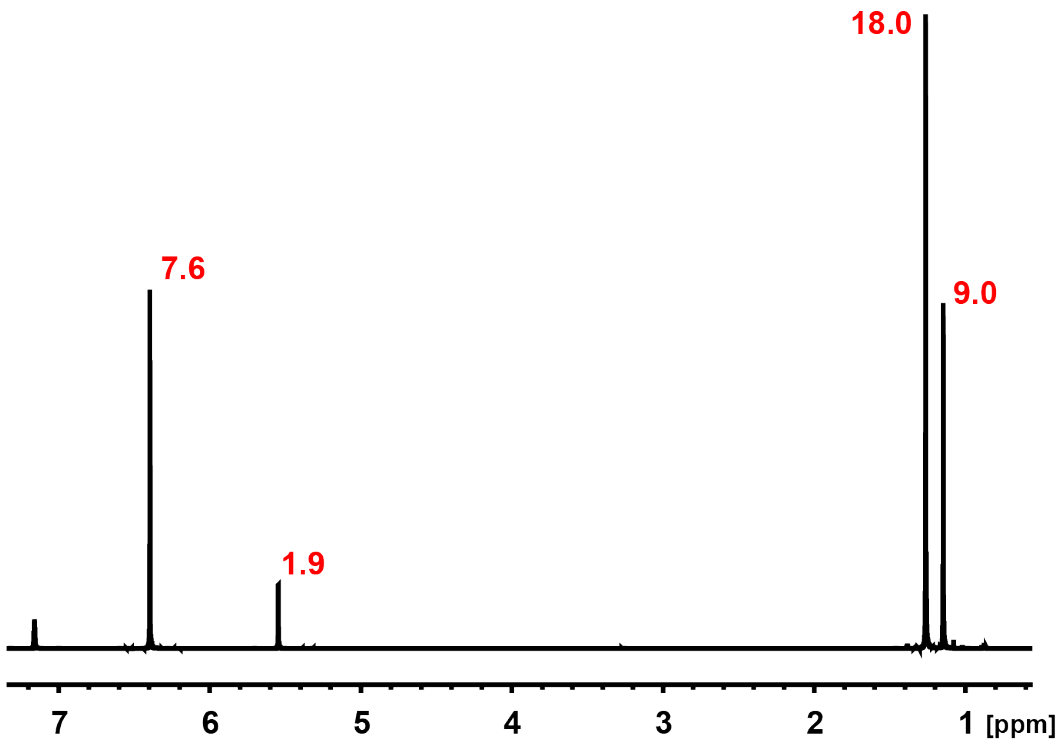

Figure S2: ^{1}H NMR spectrum of $\mathbf{Y}^{\mathbf{ttt}}$.

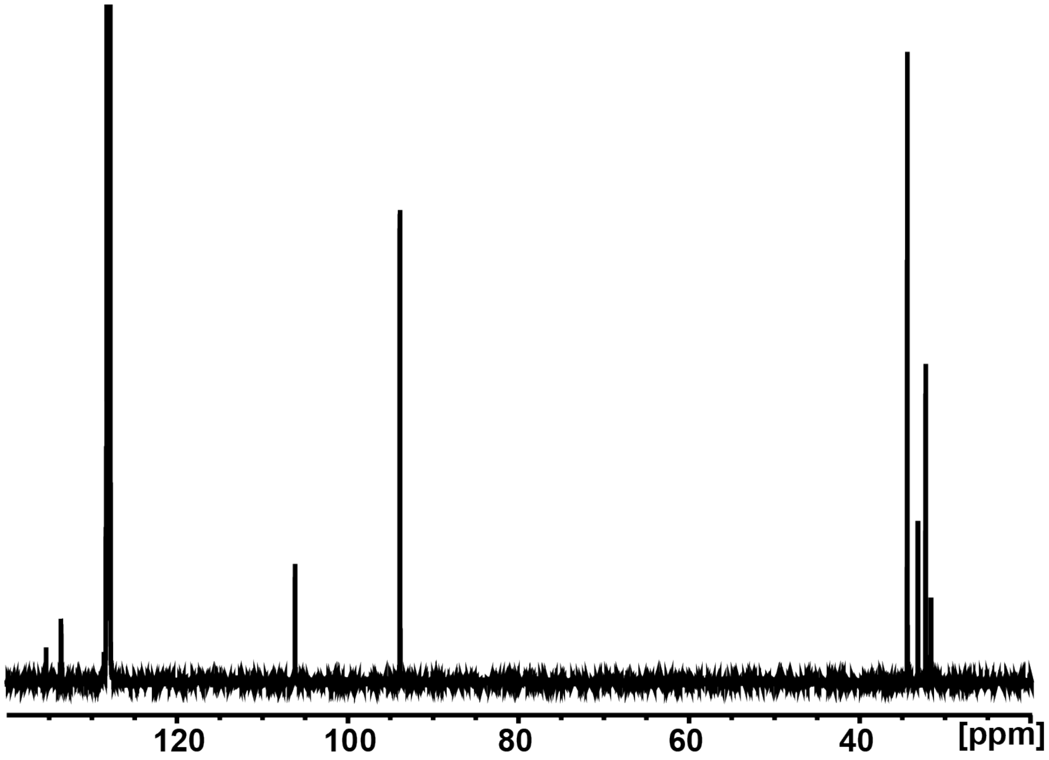

Figure S3: ^{13}C NMR spectrum of $\mathbf{Y^{ttt}}$.

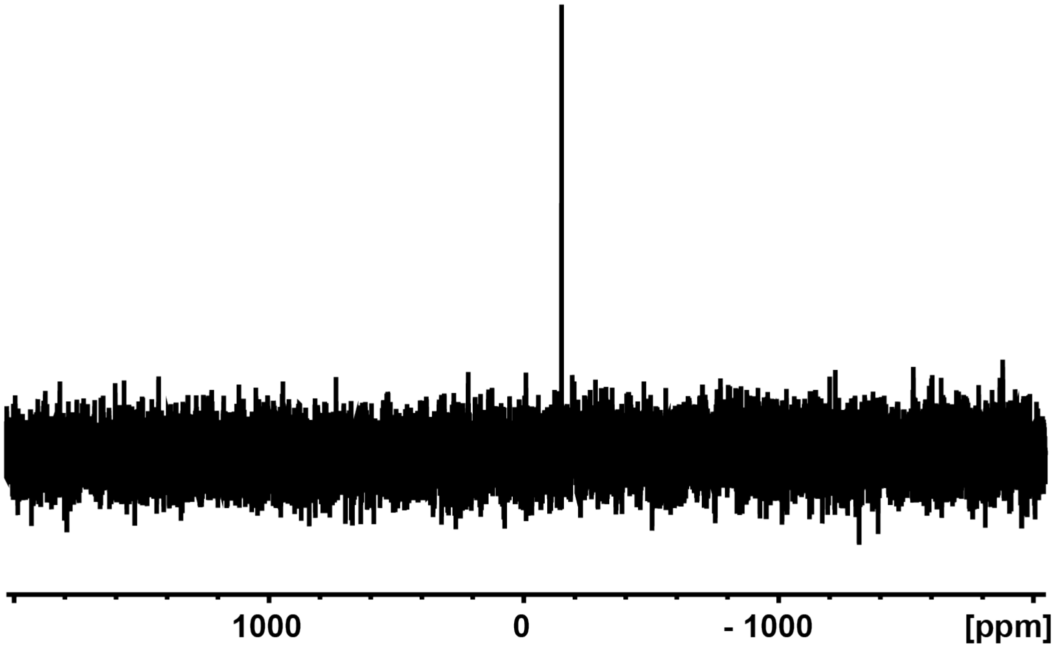

Figure S4: ^{89}Y NMR spectrum of $\mathbf{Y^{ttt}}$.

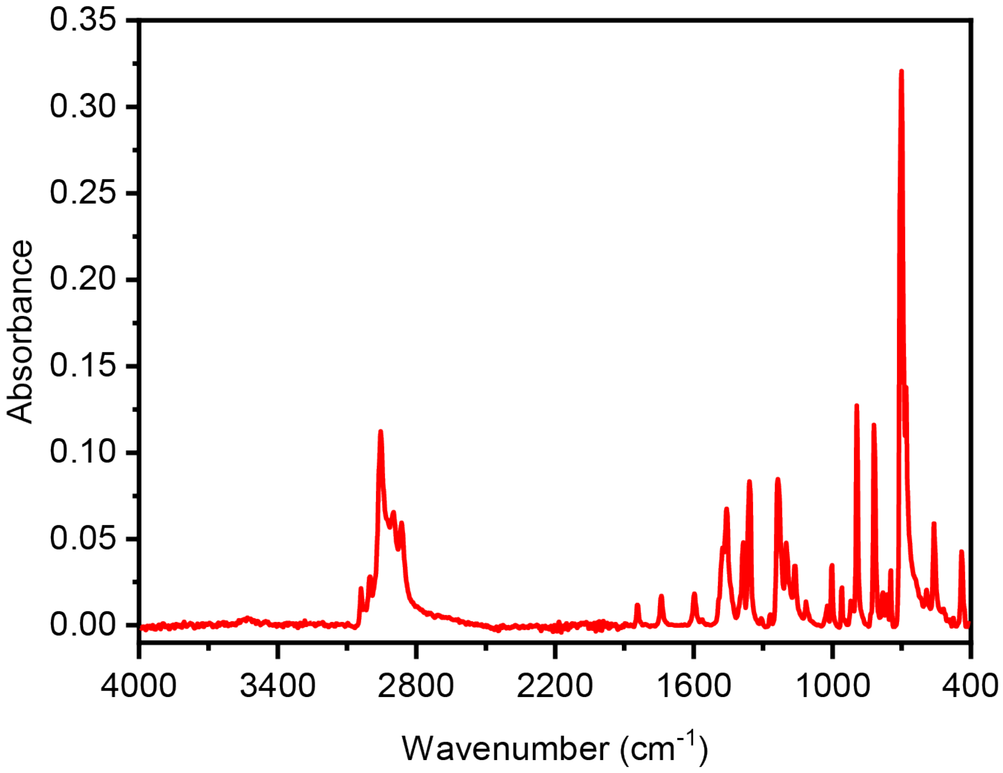

Figure S5: ATR-FTIR spectrum of $\mathbf{Dy^{ttt}}$.

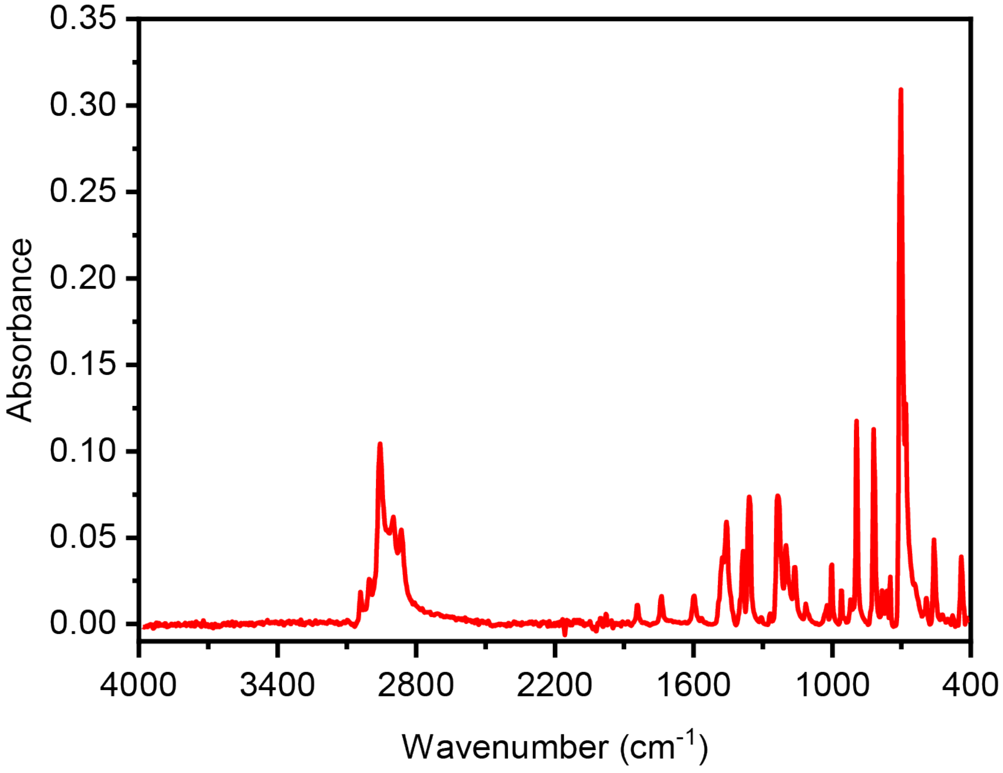

Figure S6: ATR-FTIR spectrum of $\mathbf{Er^{ttt}}$.

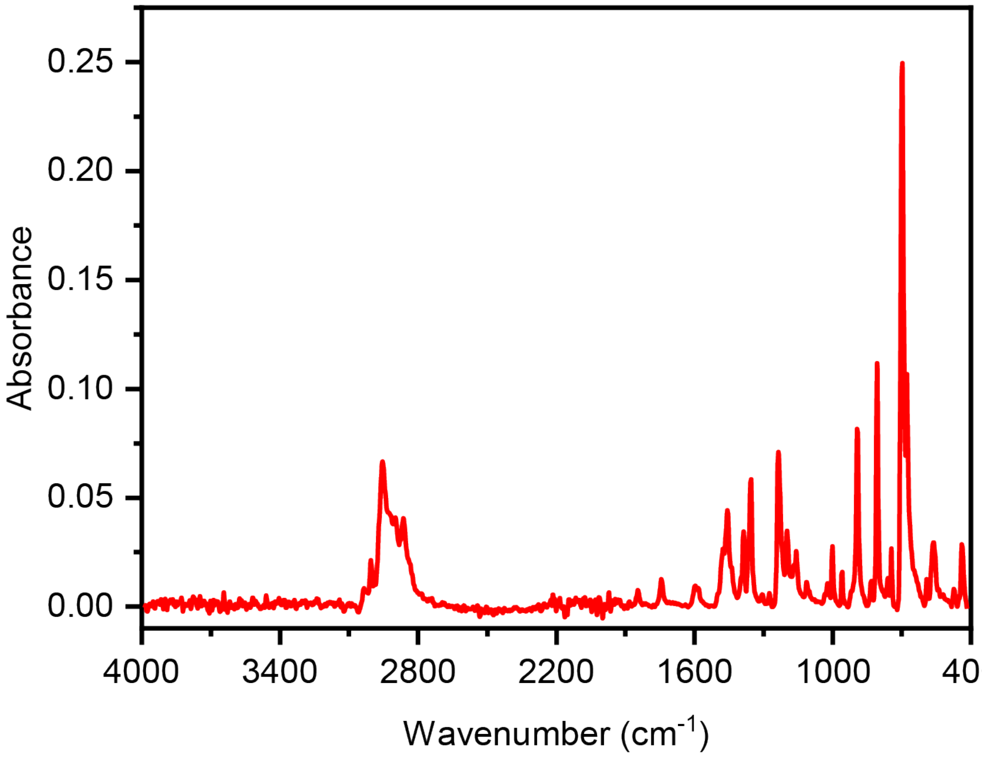

Figure S7: ATR-FTIR spectrum of $\mathbf{Nd^{ttt}}$.

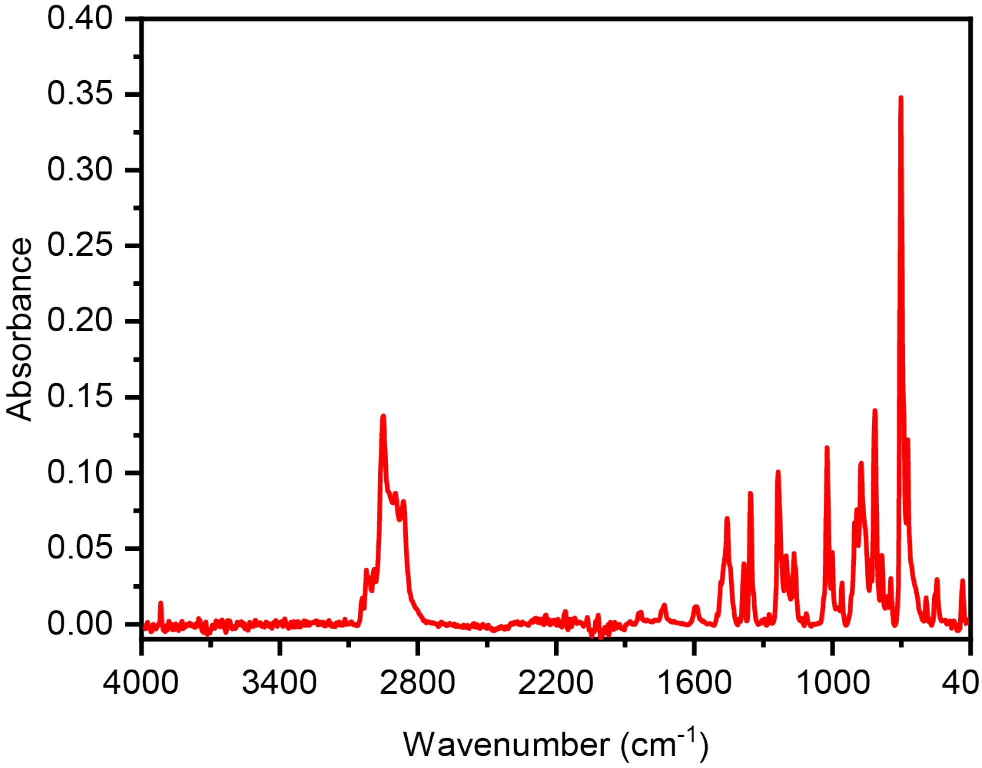

Figure S8: ATR-FTIR spectrum of $\mathbf{Nd^{ttt}}$·**THF**.

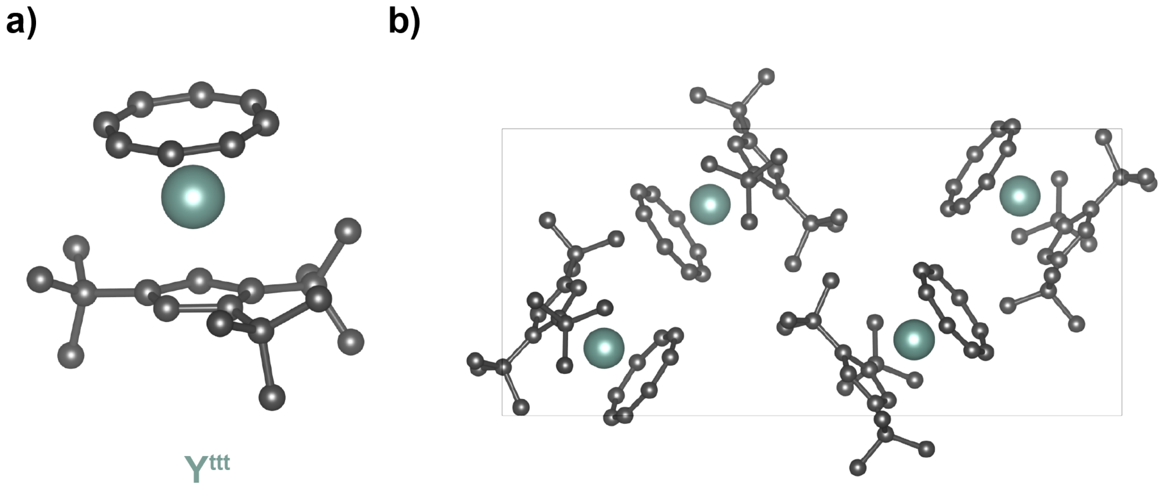

Figure S9: a) Crystal structure and b) crystal packing of **Y^{ttt}**. Hydrogen atoms were omitted for clarity.

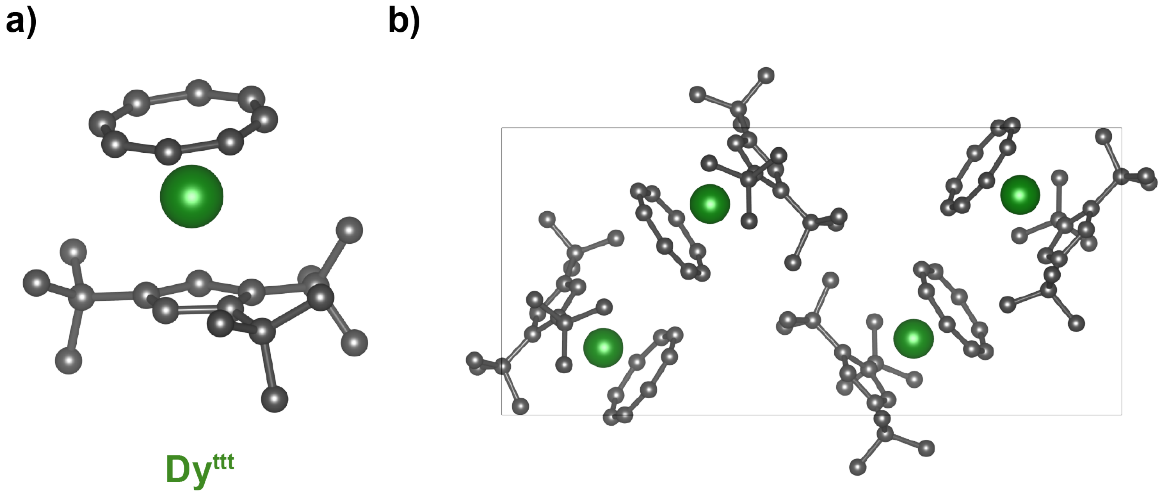

Figure S10: a) Crystal structure and b) crystal packing of **Dy^{ttt}**. Hydrogen atoms were omitted for clarity.

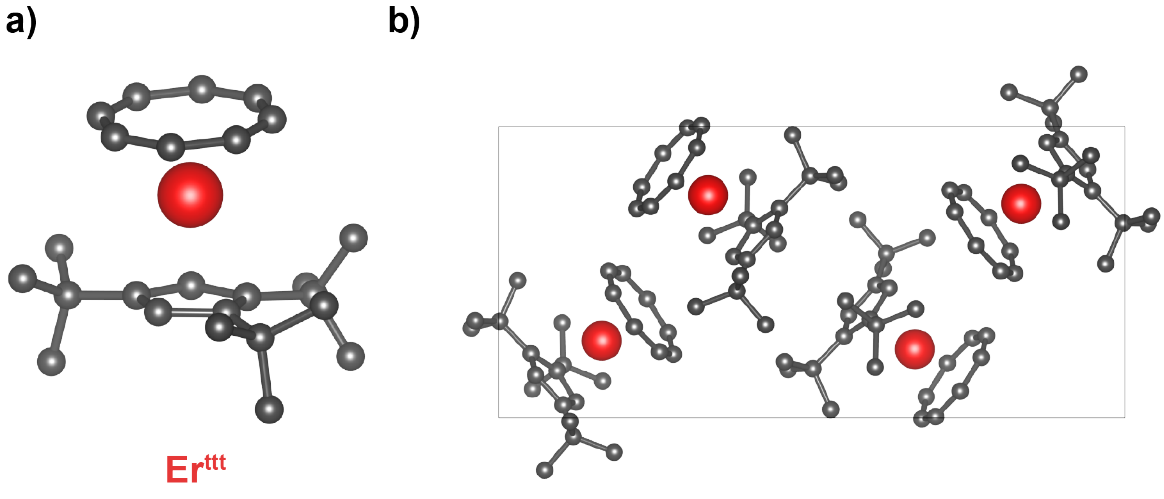

Figure S11: a) Crystal structure and b) crystal packing of **Er**$^{\mathbf{ttt}}$. Hydrogen atoms were omitted for clarity.

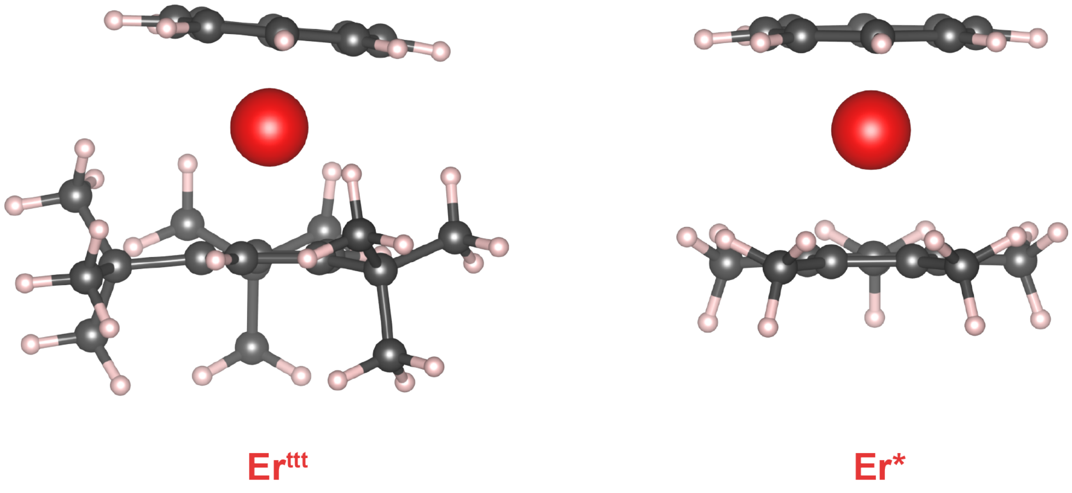

Figure S12: Comparison of optimized geometries for **Er**$^{\mathbf{ttt}}$ and **Er*** highlighting the differences in relative orientation of the five- and eight-membered rings. The geometry optimization of **Er*** leads to a nearly parallel arrangement of two rings, which stands in contrast with the collected crystal structure data. Conversely, for **Er**$^{\mathbf{ttt}}$ the calculated and experimentally determined structures correspond well with the non-parallel ring orientation.

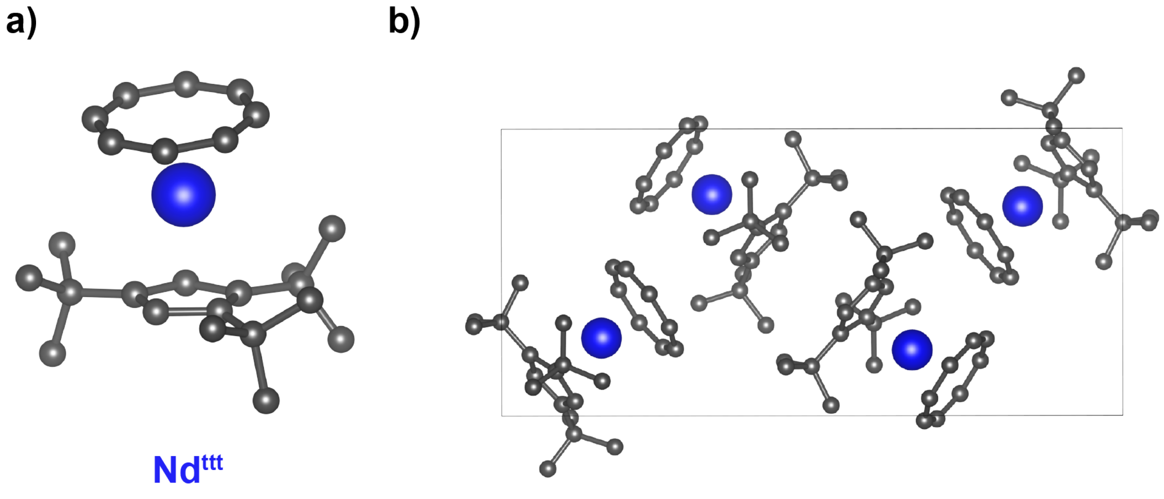

Figure S13: a) Crystal structure and b) crystal packing of **Nd$^{\mathbf{ttt}}$**. Hydrogen atoms were omitted for clarity.

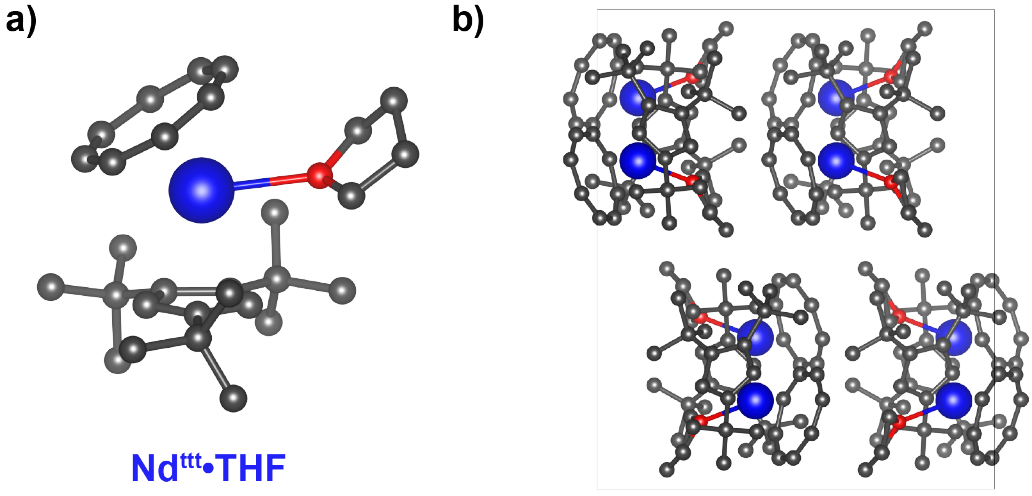

Figure S14: a) Crystal structure and b) crystal packing of **Nd$^{\mathbf{ttt}}$·THF**. Hydrogen atoms were omitted for clarity.

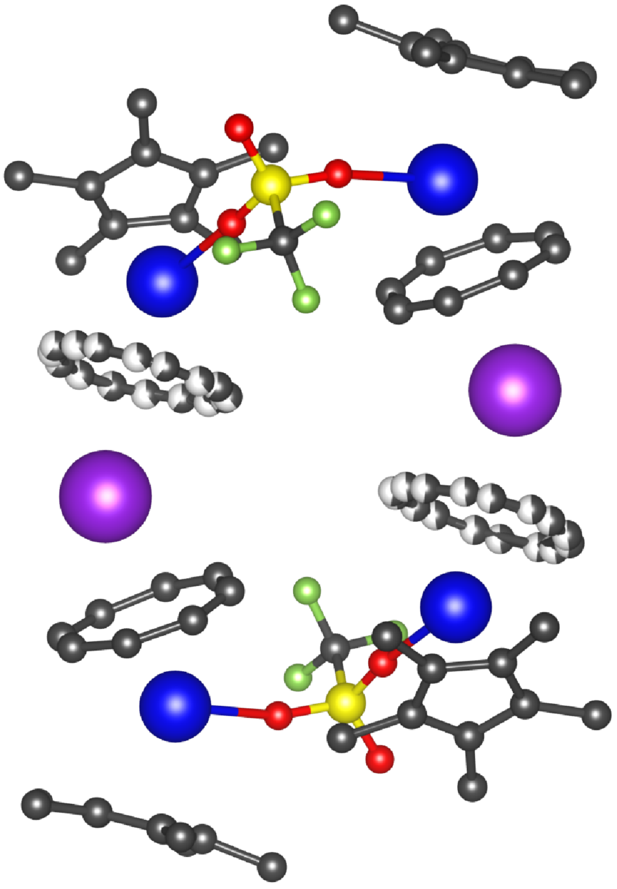

Figure S15: Crystal structure of $K_2[(COT)Nd(Cp^*)]_4(OTf)_2$. Hydrogen atoms and the disordered solvent molecules were omitted for clarity.

Table S1: Details of crystal structure determination for $\mathbf{Y^{ttt}}$, $\mathbf{Dy^{ttt}}$ and $\mathbf{Er^{ttt}}$.

**Compound**	$\mathbf{Y^{ttt}}$	$\mathbf{Dy^{ttt}}$	$\mathbf{Er^{ttt}}$
**CCDC number**	2170514	2170518	2170516
**Chemical formula**	$C_{25}H_{37}Y$	$C_{25}H_{37}Dy$	$C_{25}H_{37}Er$
**Formula weight**	427.48 g·mol^{-1}	500.04 g·mol^{-1}	504.80 g·mol^{-1}
**Temperature**	100.0 K	100.02 K	101.96 K
**Crystal system**	Monoclinic	Monoclinic	Monoclinic
**Space group**	$P2_1/n$	$P2_1/n$	$P2_1/n$
**a**	10.1312(3) Å	10.1186(8) Å	10.1016(9) Å
**b**	21.6836(6) Å	21.6751(17) Å	21.6348(19) Å
**c**	10.1764(3) Å	10.1771(8) Å	10.1842(9) Å
**α**	90°	90°	90°
**β**	99.1673(14)°	99.224(3)°	99.328(4)°
**γ**	90°	90°	90°
**Volume**	2207.01(11) Å^3	2203.2(3) Å^3	2196.3(3) Å^3
**Z**	4	4	4
**F(000)**	893.068	1012	1020
**Reflections measured**	41069	113975	41560
**Independent reflections**	7506 ($R_{int} = 0.0421$)	6739 ($R_{int} = 0.0743$)	5031 ($R_{int} = 0.0389$)
**Data/restraints/parameters**	7506/56/383	6739/100/383	5031/48/346
**Final R indices (I>2σ(I))**	$R_1 = 0.0341$, $wR_2 = 0.0726$	$R_1 = 0.0234$, $wR_2 = 0.0413$	$R_1 = 0.0190$, $wR_2 = 0.0429$
**R indices (all data)**	$R_1 = 0.0636$, $wR_2 = 0.0797$	$R_1 = 0.0355$, $wR_2 = 0.0439$	$R_1 = 0.0223$, $wR_2 = 0.0439$
**Goodness-of-fit**	1.0356	1.024	1.059

Table S2: Details of crystal structure determination for **Nd^{ttt}**, **Nd^{ttt}·THF** and $K_2[(COT)Nd(Cp^*)]_4(OTf)_2$.

**Compound**	**Nd^{ttt}**	**Nd^{ttt}·THF**	**$K_2[(COT)Nd(Cp^*)]_4(OTf)_2$**
**CCDC number**	2170515	2170525	2170517
**Chemical formula**	$C_{25}H_{37}Nd$	$C_{29}H_{45}NdO$	$C_{102}H_{124}F_6K_2Nd_4O_6S_2$
**Formula weight**	481.78 g·mol^{-1}	553.92 g·mol^{-1}	2279.28 g·mol^{-1}
**Temperature**	100.00 K	99.99 K	100.0 K
**Crystal system**	Monoclinic	Orthorhombic	Monoclinic
**Space group**	$P2_1/n$	Pbca	$P2_1/n$
**a**	10.1901(5) Å	18.3040(4) Å	21.4669(14) Å
**b**	21.8865(12) Å	15.4886(3) Å	18.3458(12) Å
**c**	10.2204(5) Å	18.7216(4) Å	25.0418(16) Å
**α**	90°	90°	90°
**β**	98.782(5)°	90°	100.7174(13)°
**γ**	90°	90°	90°
**Volume**	2252.7(2) Å^3	5307.64(19) Å^3	9690.1(11) Å^3
**Z**	4	8	4
**F(000)**	988	2296.870	4592
**Reflections measured**	47977	309546	178690
**Independent reflections**	5342 (R_{int} = 0.0637)	9241 (R_{int} = 0.0877)	22184 (R_{int} = 0.0570)
**Data/restraints/parameters**	5342/81/376	9241/56/460	22184/238/1164
**Final R indices (I>2σ(I))**	R_1 = 0.0346, wR_2 = 0.0586	R_1 = 0.0228, wR_2 = 0.0399	R_1 = 0.0413, wR_2 = 0.0887
**R indices (all data)**	R_1 = 0.0445, wR_2 = 0.0615	R_1 = 0.0404, wR_2 = 0.0501	R_1 = 0.0718, wR_2 = 0.1046
**Goodness-of-fit**	1.156	1.1344	1.121

Table S3: Selected bond and angle crystal metrics for $\mathbf{Y^{ttt}}$, $\mathbf{Dy^{ttt}}$, $\mathbf{Er^{ttt}}$, $\mathbf{Nd^{ttt}}$ and $\mathbf{Nd^{ttt}{\cdot}THF}$. C1-C5 denote carbon atoms belonging to the $(Cp^{ttt})^{-}$ ring and C6-C13 carbon atoms belonging to the $(COT)^{2-}$ ring. Cent. stands for centroid.

	$\mathbf{Y^{ttt}}$	$\mathbf{Dy^{ttt}}$	$\mathbf{Er^{ttt}}$	$\mathbf{Nd^{ttt}}$	$\mathbf{Nd^{ttt}{\cdot}THF}$
**M–C1**	2.6095(14) Å	2.6160(18) Å	2.590(2) Å	2.711(3) Å	2.7895(17) Å
**M–C2**	2.6144(15) Å	2.6152(18) Å	2.593(2) Å	2.708(3) Å	2.7955(16) Å
**M–C3**	2.6788(15) Å	2.67781(18) Å	2.649(2) Å	2.759(3) Å	2.8260(17) Å
**M–C4**	2.6495(14) Å	2.6546(18) Å	2.629(2) Å	2.745(3) Å	2.7738(17) Å
**M–C5**	2.5954(14) Å	2.6046(19) Å	2.580(2) Å	2.709(3) Å	2.7258(17) Å
**M–C6**	2.5529(17) Å	2.558(2) Å	2.509(2) Å	2.655(3) Å	2.6737(19) Å
**M–C7**	2.5491(16) Å	2.553(2) Å	2.519(2) Å	2.652(3) Å	2.646(2) Å
**M–C8**	2.5480(17) Å	2.547(2) Å	2.529(2) Å	2.645(3) Å	2.674(2) Å
**M–C9**	2.5459(17) Å	2.544(2) Å	2.525(2) Å	2.644(3) Å	2.654(2) Å
**M–C10**	2.5323(16) Å	2.535(2) Å	2.524(2) Å	2.633(3) Å	2.659(2) Å
**M–C11**	2.5252(17) Å	2.530(2) Å	2.523(2) Å	2.628(3) Å	2.7174(19) Å
**M–C12**	2.5281(17) Å	2.528(2) Å	2.512(2) Å	2.623(3) Å	2.759(2) Å
**M–C13**	2.5440(18) Å	2.549(2) Å	2.507(2) Å	2.640(3) Å	2.731(2) Å
$\mathbf{M–cent._{Cp}}$	2.334 Å	2.339 Å	2.310 Å	2.443 Å	2.505 Å
$\mathbf{M–cent._{COT}}$	1.751 Å	1.756 Å	1.718 Å	1.895 Å	1.965 Å
$\boldsymbol{\omega}$	175.93°	175.25°	175.93°	173.07°	147.45°

Calculations of percent buried volume in the investigated complexes

To gain an insight into the steric differences between the newly synthesized $(Cp^{ttt})^-$ and the published $(Cp^*)^-$ derivatives we performed calculations of the 'percent buried volume' using the SambVca 2.1 software package.[44] This parameter quantifies the volume fraction of the imaginary sphere of a pre-defined radius that is centered around the metal ion that is occupied by the ligand. Consequently, it can be used as a gauge for the steric properties of the ligand environment.[45] We used the experimentally determined SCXRD structures as a starting point for the buried volume calculations. The sphere radius was selected based on the distance to the furthest methyl carbon belonging to the *tert*-butyl moieties located on the same side of the cyclopentadienyl ring as the metal center. This was kept constant across derivatives containing the same metal ions (e.g. $\mathbf{Er^{ttt}}$ and **Er***) to ensure comparability of the results. As anticipated, the presence of the $(Cp^{ttt})^-$ ligand leads to an increased buried volume compared with the respective $(Cp^*)^-$ containing species. These results substantiate our conclusions regarding the impact of the ligand sterics on the solid-state structure of the compounds discussed in this report.

Table S4: Calculations of buried volume for the representative late and early lanthanides derivatives. # Hypothetical Nd* fragment extracted from $K_2[(COT)Nd(Cp^*)]_4(OTf)_2$.

	$\mathbf{Er^{ttt}}$	$\mathbf{Nd^{ttt}}$	**Er***	Nd*#
Buried volume [%]	87.8	87.0	83.4	80.0
Free volume [%]	12.2	13.0	16.6	20.0
Radius (Å)	4.13254	4.12584	4.13254	4.12584

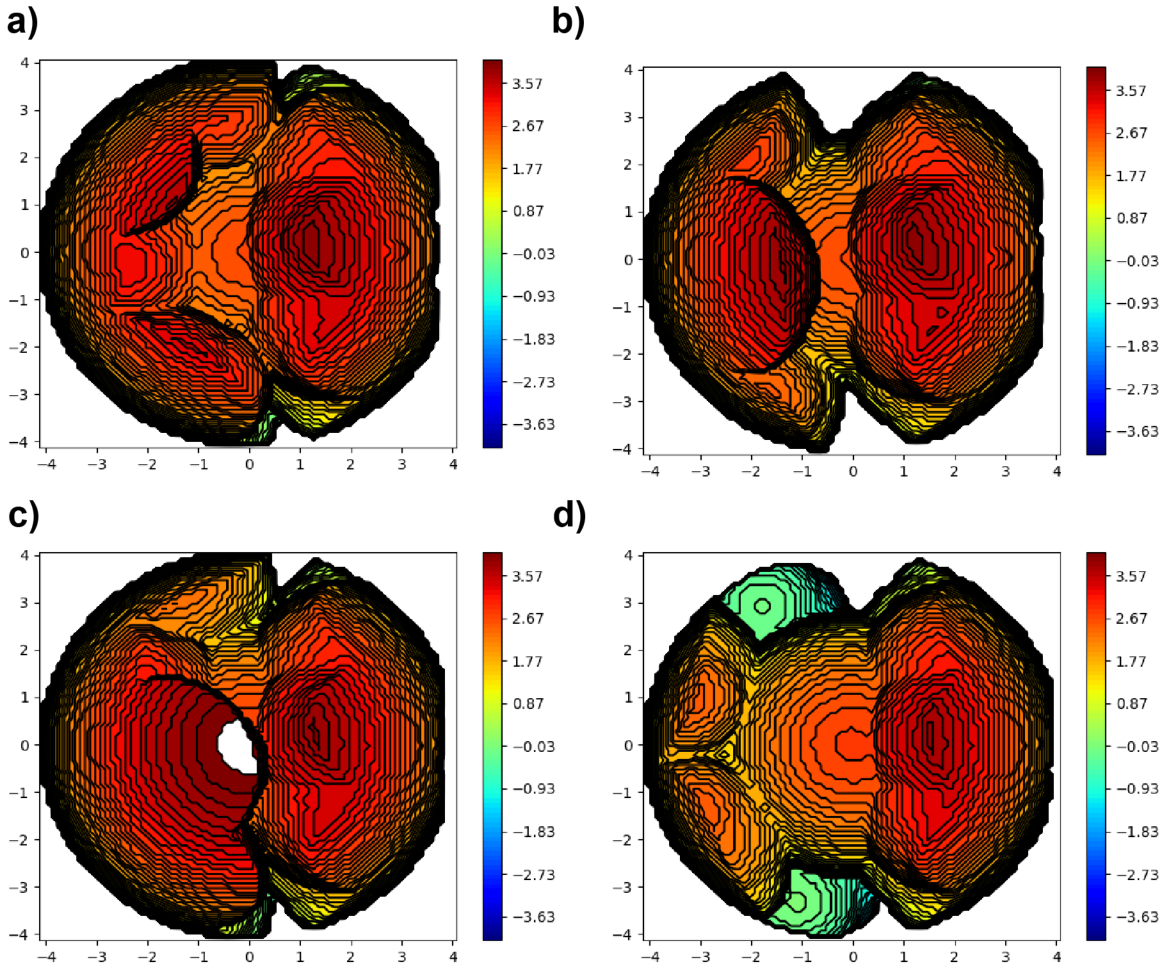

Figure S16: Steric maps obtained in the course of buried volume calculations using SambVca 2.1 software package for: a) **Er^{ttt}**, b) **Er***, c) **Nd^{ttt}** and d) hypothetical Nd* fragment extracted from the $K_2[(COT)Nd(Cp^*)]_4(OTf)_2$ structure.

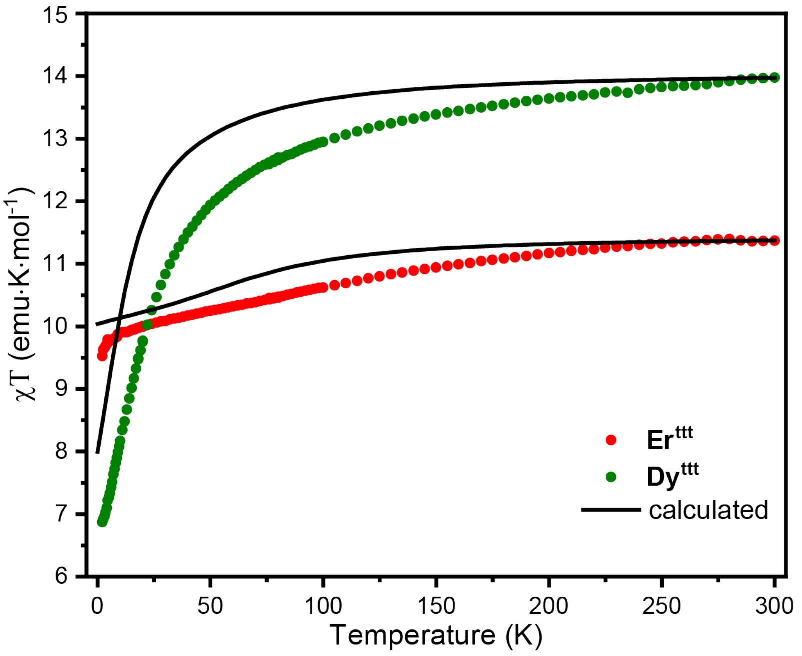

Figure S17: Temperature dependence of χ_MT for **Er^{ttt}** and **Dy^{ttt}** measured between 2 and 300 K. The experimental data points were rescaled by 1.33 and 1.45 for Er(III) and Dy(III) derivatives, respectively. Calculated curves are depicted for comparison.

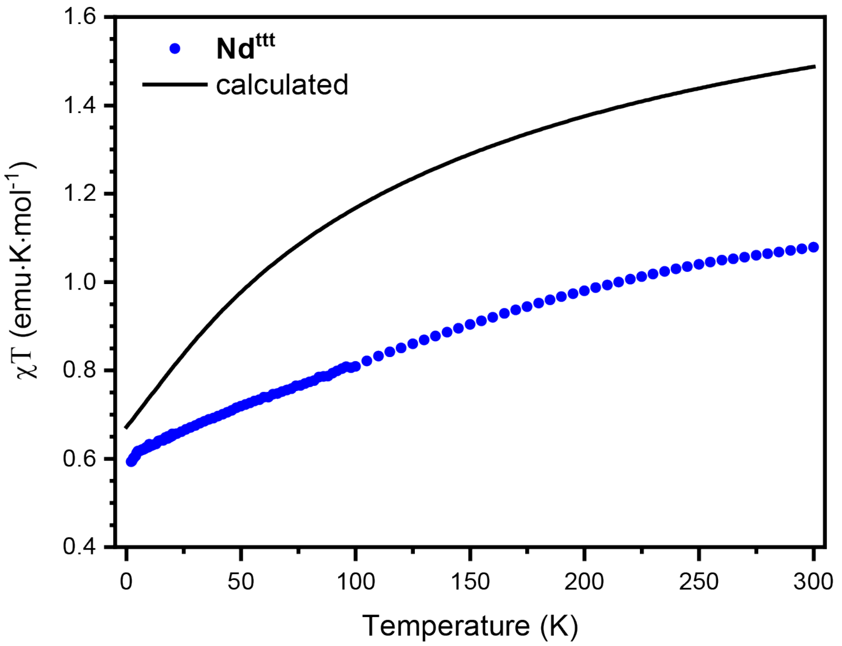

Figure S18: Temperature dependence of χ_MT for **Nd^{ttt}** measured between 2 and 300 K. Calculated curves (CASPT2) are depicted for comparison.

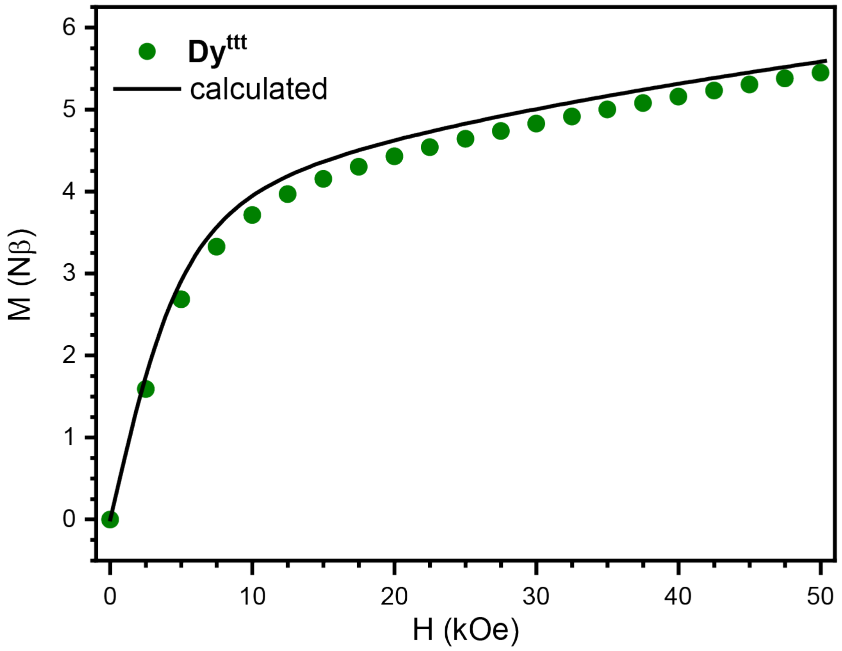

Figure S19: Magnetization of **Dy^{ttt}** as a function of the applied field collected at 2 K (rescaled by 1.45). Calculated curves are depicted for comparison.

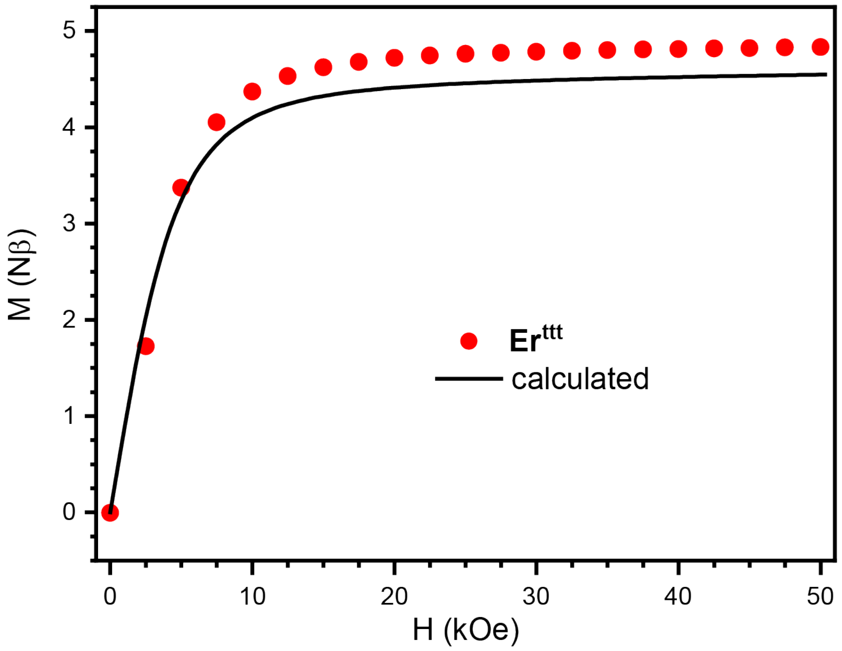

Figure S20: Magnetization of **Er^{ttt}** as a function of the applied field collected at 2 K (rescaled by 1.33). Calculated curves are depicted for comparison.

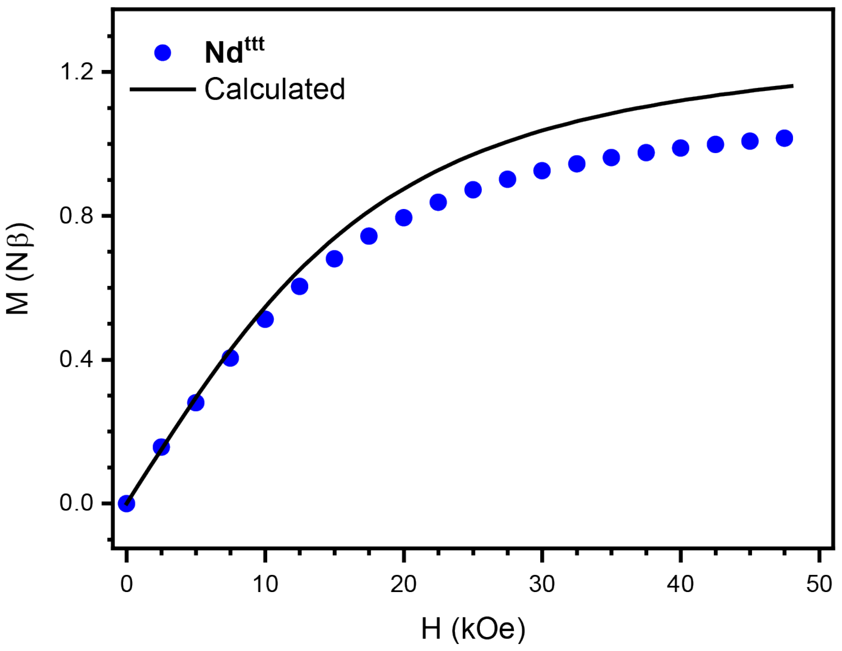

Figure S21: Magnetization of **Nd^{ttt}** as a function of the applied field collected at 2 K. Calculated curves (CASPT2) are depicted for comparison.

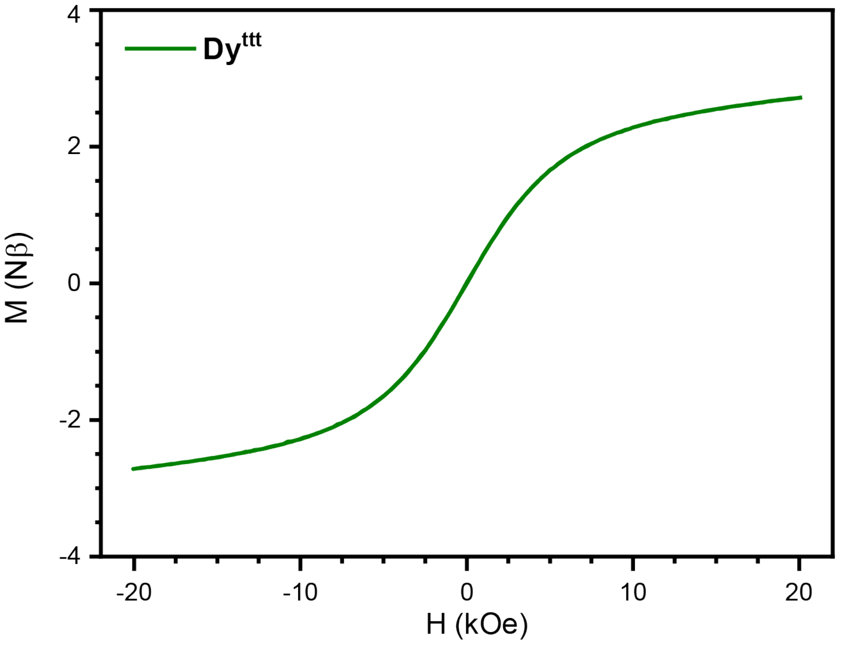

Figure S22: Hysteresis loop measured at 2 K and at 16 Oe·s^{-1} for **Dy^{ttt}**.

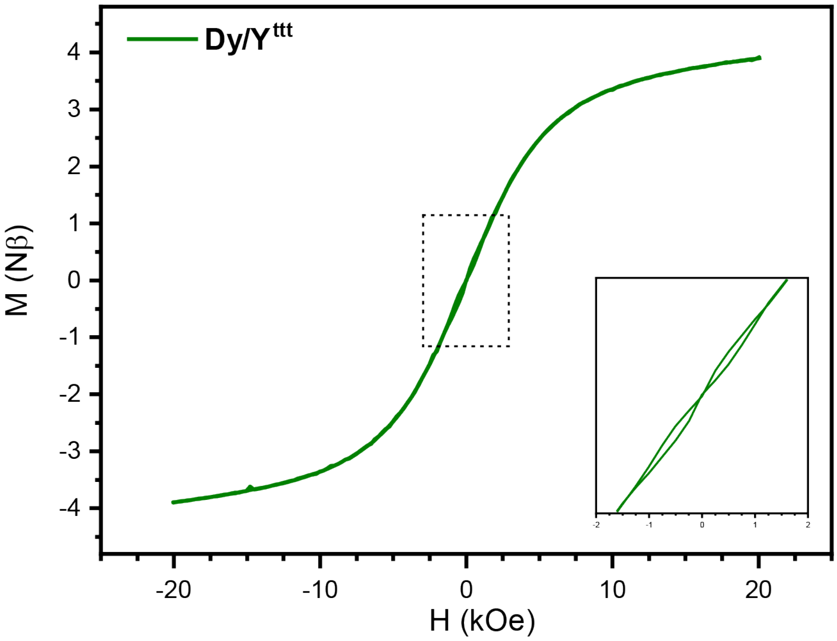

Figure S23: Hysteresis loop measured at 2 K and at 16 Oe·s^{-1} for **Dy/Y^{ttt}**.

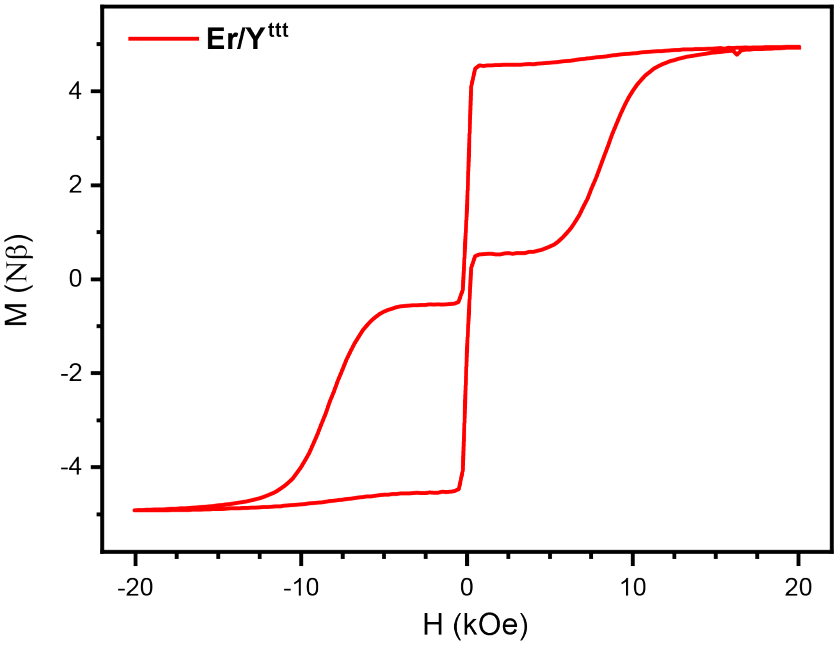

Figure S24: Hysteresis loop measured at 2 K and at 16 Oe·s^{-1} for **Er/Y^{ttt}**.

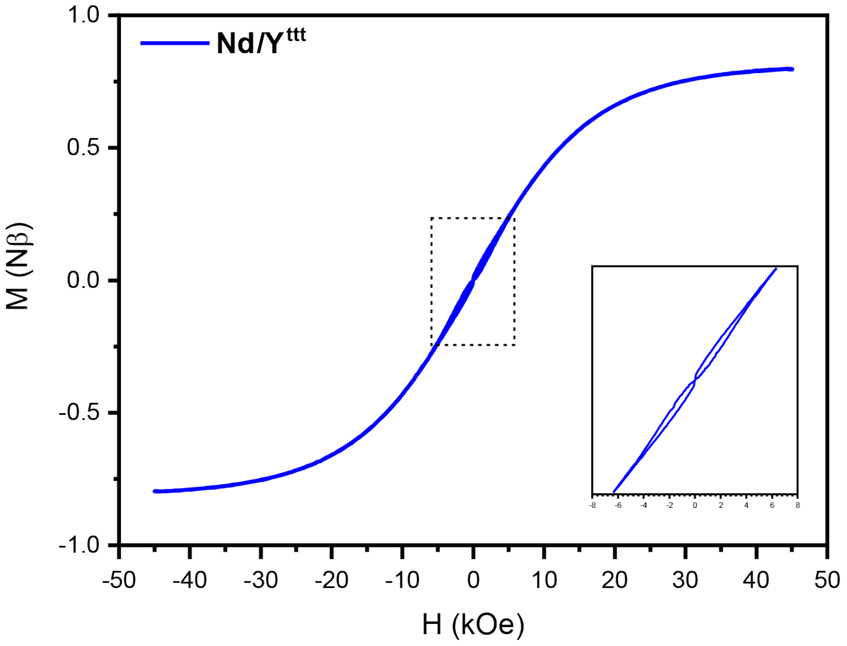

Figure S25: Hysteresis loop measured at 2 K and at 16 Oe·s^{-1} for **Nd/Y^{ttt}**.

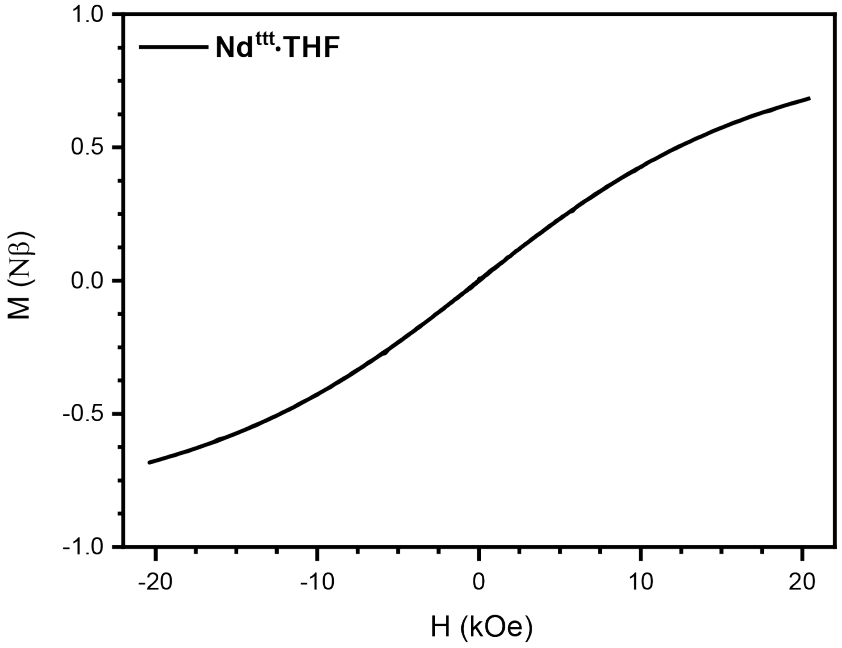

Figure S26: Hysteresis loop measured at 2 K and at 16 Oe·s^{-1} for **Nd^{ttt}·THF**.

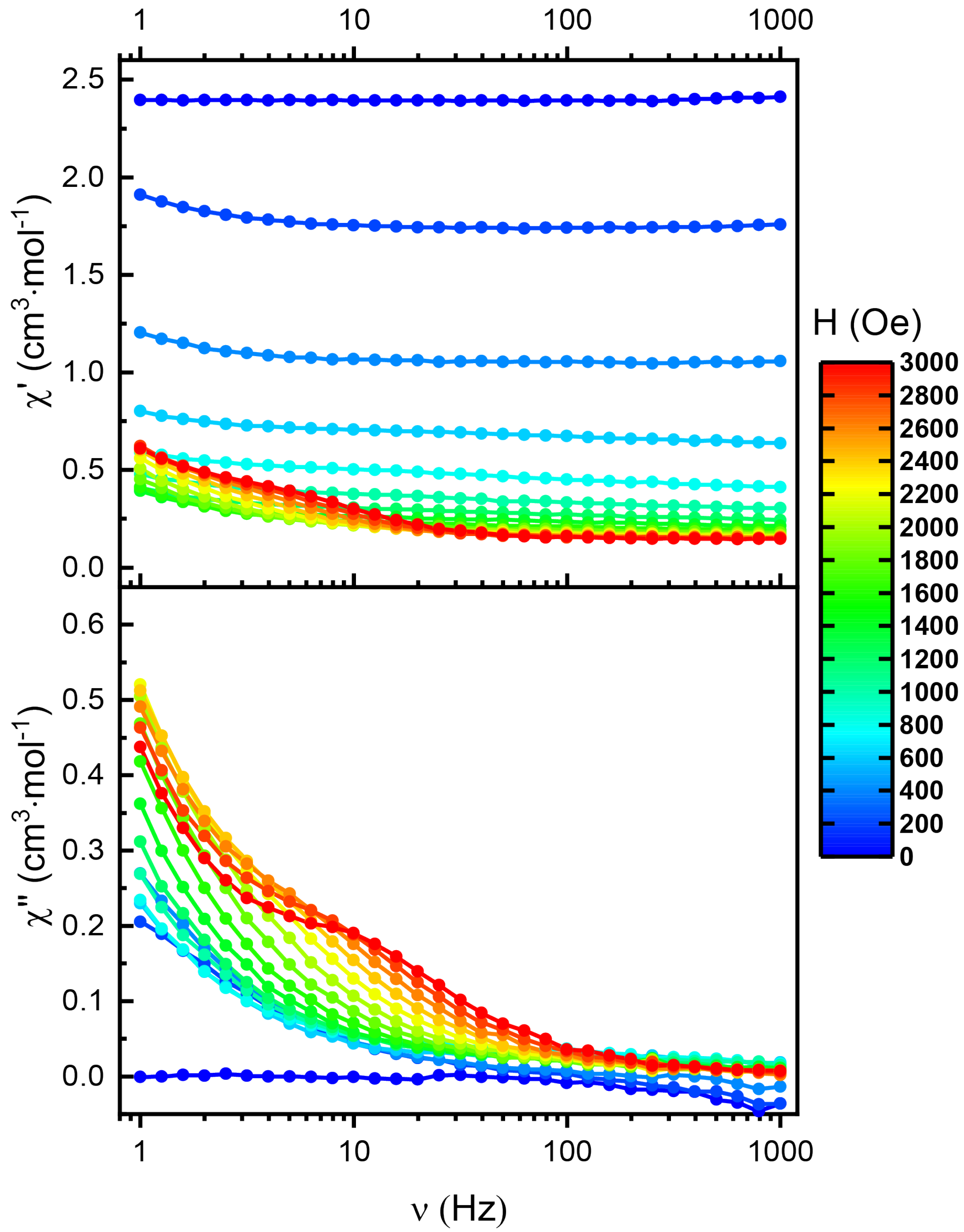

Figure S27: Frequency dependence of both in-phase (top) and out-of-phase (down) components of the AC susceptibility measured at 2 K with DC field varying between 0 and 3000 Oe for **Dy$^{\mathbf{ttt}}$**. Circles represent experimental data points; the lines do not have any physical meaning.

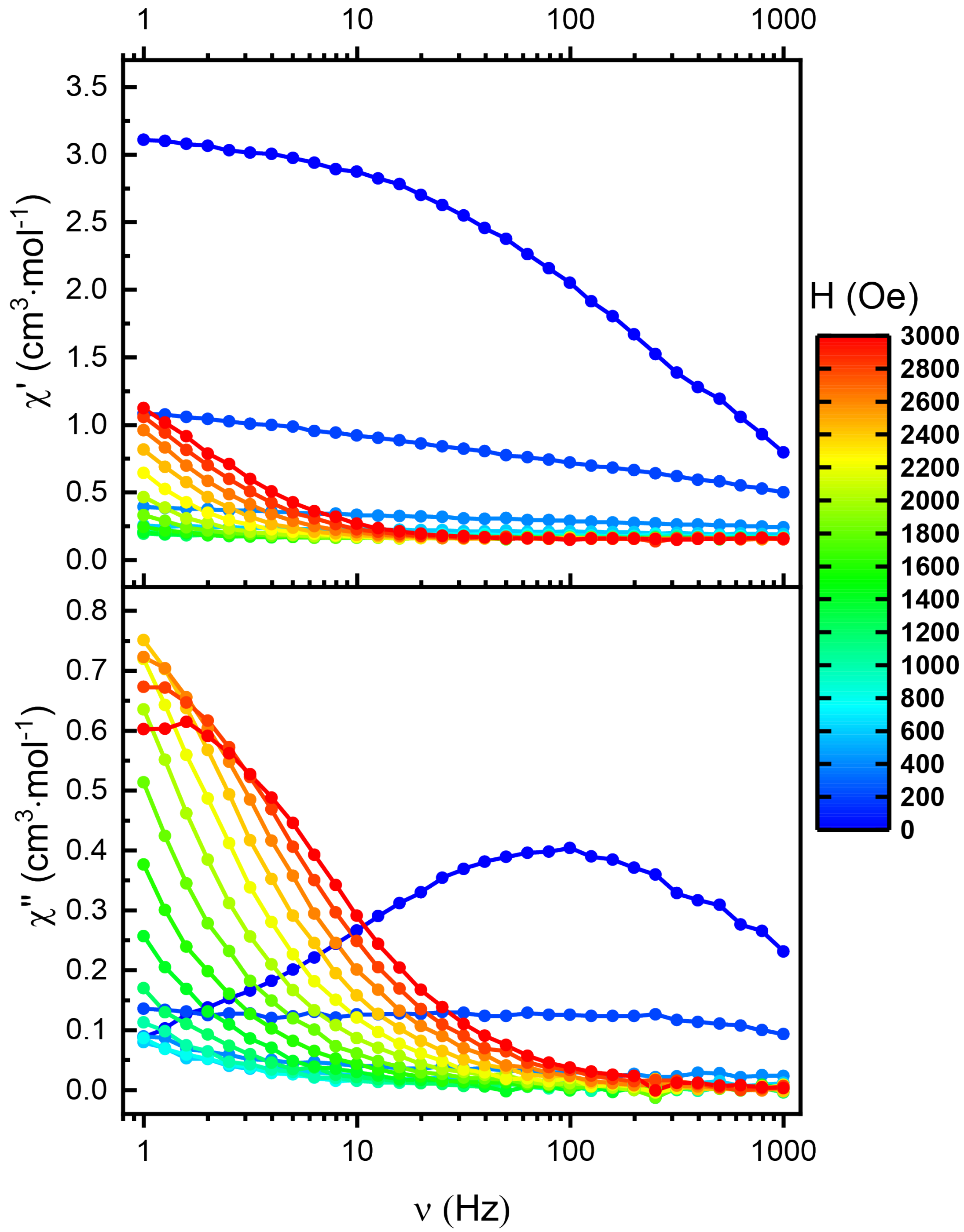

Figure S28: Frequency dependence of both in-phase (top) and out-of-phase (down) components of the AC susceptibility measured at 2 K with DC field varying between 0 and 3000 Oe for **Dy/Y$^{\mathbf{ttt}}$**. Circles represent experimental data points; the lines do not have any physical meaning.

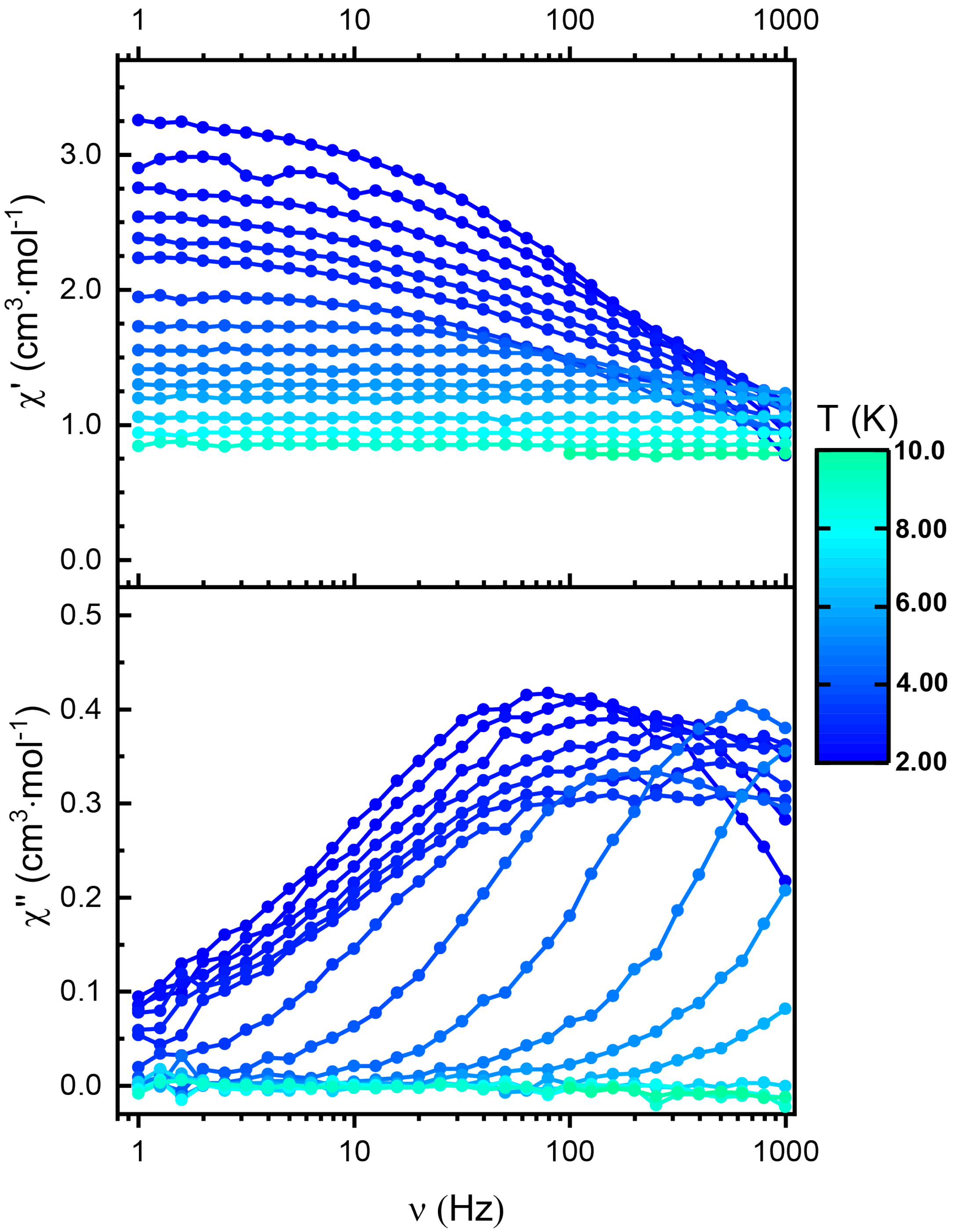

Figure S29: Frequency dependence of both in-phase (top) and out-of-phase (down) components of the AC susceptibility measured in zero external DC field between 2 and 10 K for **Dy/Y^{ttt}**. Circles represent experimental data points; the lines do not have any physical meaning.

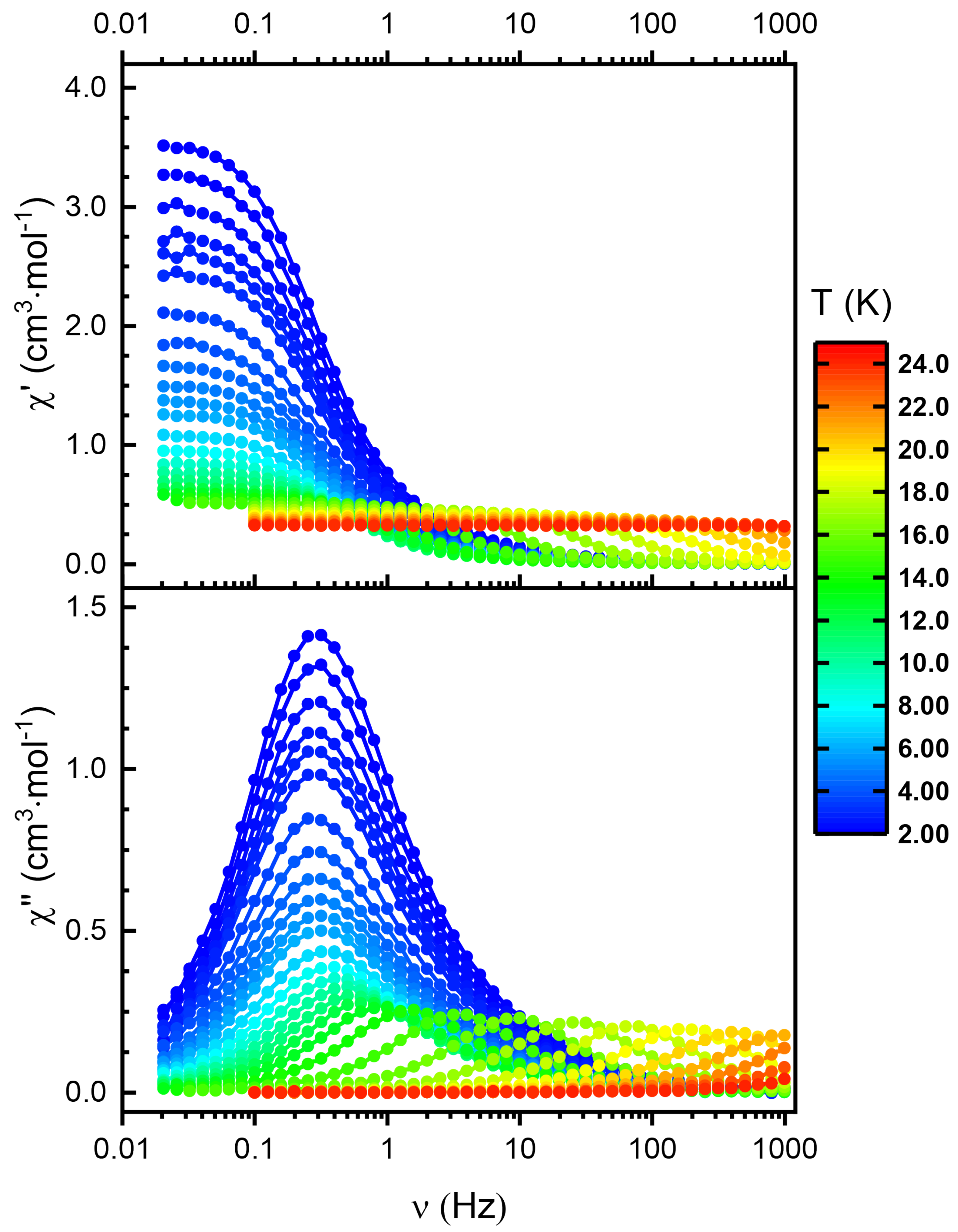

Figure S30: Frequency dependence of both in-phase (top) and out-of-phase (down) components of the AC susceptibility measured in zero external DC field between 2 and 24 K for $\mathbf{Er^{ttt}}$. Circles represent experimental data points; the lines do not have any physical meaning.

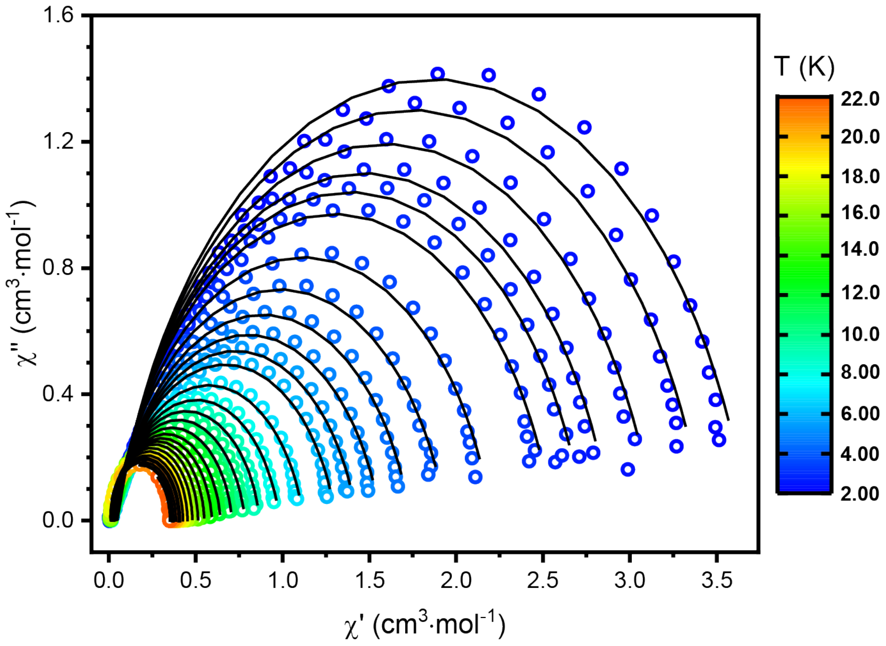

Figure S31: Variations of χ” as function of χ’ (circles) for **Er$^{\mathbf{ttt}}$** in zero external DC field. Circles represent experimental data points and lines represent the best fitted curves with extended Debye model.

Table S5: Best fitted parameters with the extended Debye model for $\mathbf{Er^{ttt}}$ at zero external DC field.

**T (K)**	χ_T **(cm³·mol⁻¹)**	χ_S **(cm³·mol⁻¹)**	α	τ**(s)**	R^2
2	3.68405	0.04879	0.16516	0.47193	0.99848
2.2	3.43154	0.04645	0.16573	0.47677	0.99843
2.4	3.14241	0.04342	0.16452	0.47966	0.99831
2.6	2.89428	0.04039	0.16387	0.48096	0.9982
2.8	2.73906	0.03822	0.16434	0.48328	0.9982
3	2.56165	0.03594	0.16549	0.48615	0.99835
3.5	2.209	0.02981	0.16818	0.48798	0.99845
4	1.94553	0.02688	0.17006	0.48397	0.99848
4.5	1.73803	0.03555	0.16734	0.46835	0.99841
5	1.56534	0.03318	0.1658	0.45512	0.99836
5.5	1.42929	0.03109	0.16544	0.44382	0.99844
6	1.309	0.02901	0.16238	0.42951	0.99828
7	1.12347	0.02649	0.1551	0.40336	0.99835
8	0.98395	0.02403	0.14376	0.37279	0.99851
9	0.86958	0.02238	0.12629	0.33372	0.99846
10	0.78356	0.02075	0.11327	0.29581	0.9987
11	0.70989	0.01901	0.10066	0.25539	0.99885
12	0.64351	0.01746	0.08373	0.2115	0.99906
13	0.59411	0.01603	0.06538	0.16419	0.99927
14	0.55007	0.01375	0.04268	0.10221	0.99956
15	0.51386	0.01247	0.02405	0.04601	0.99983
16	0.48112	0.00974	0.01499	0.01702	0.99993
17	0.45257	0.00863	0.0084	0.00612	0.99992
18	0.42735	0.00933	0.00676	2.27E-03	0.99991
19	0.40604	0.00803	0.00918	8.86E-04	0.99992
20	0.38619	0.00907	0.00953	3.71E-04	0.99993
21	0.36797	0.01184	0.00397	1.68E-04	0.99992
22	0.35183	0.0137	1.16E-17	8.03E-5	0.99992

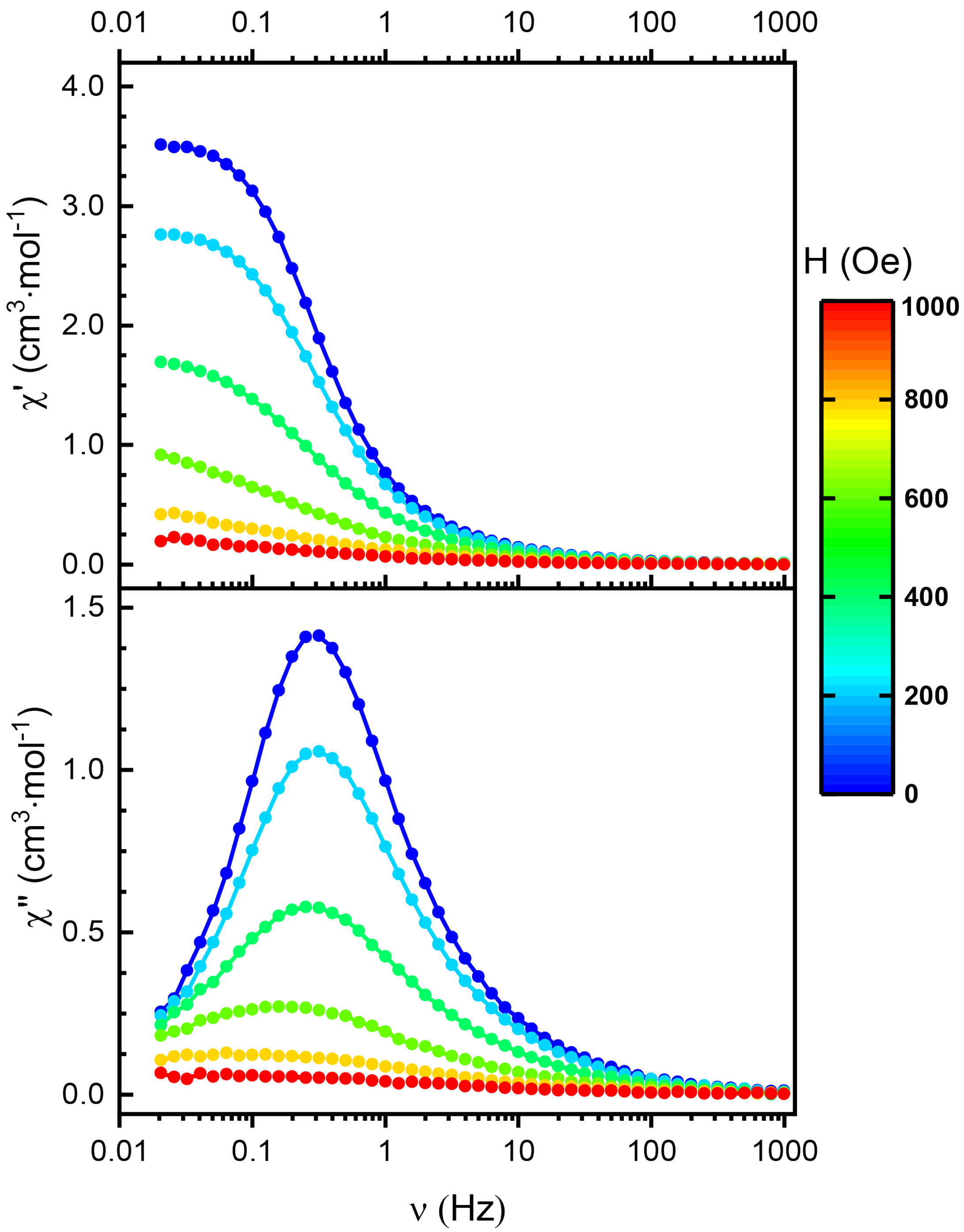

Figure S32: Frequency dependence of both in-phase (top) and out-of-phase (down) components of the AC susceptibility measured at 2 K with DC field varying between 0 and 1000 Oe for **Er**$^{\mathbf{ttt}}$. Circles represent experimental data points; the lines do not have any physical meaning.

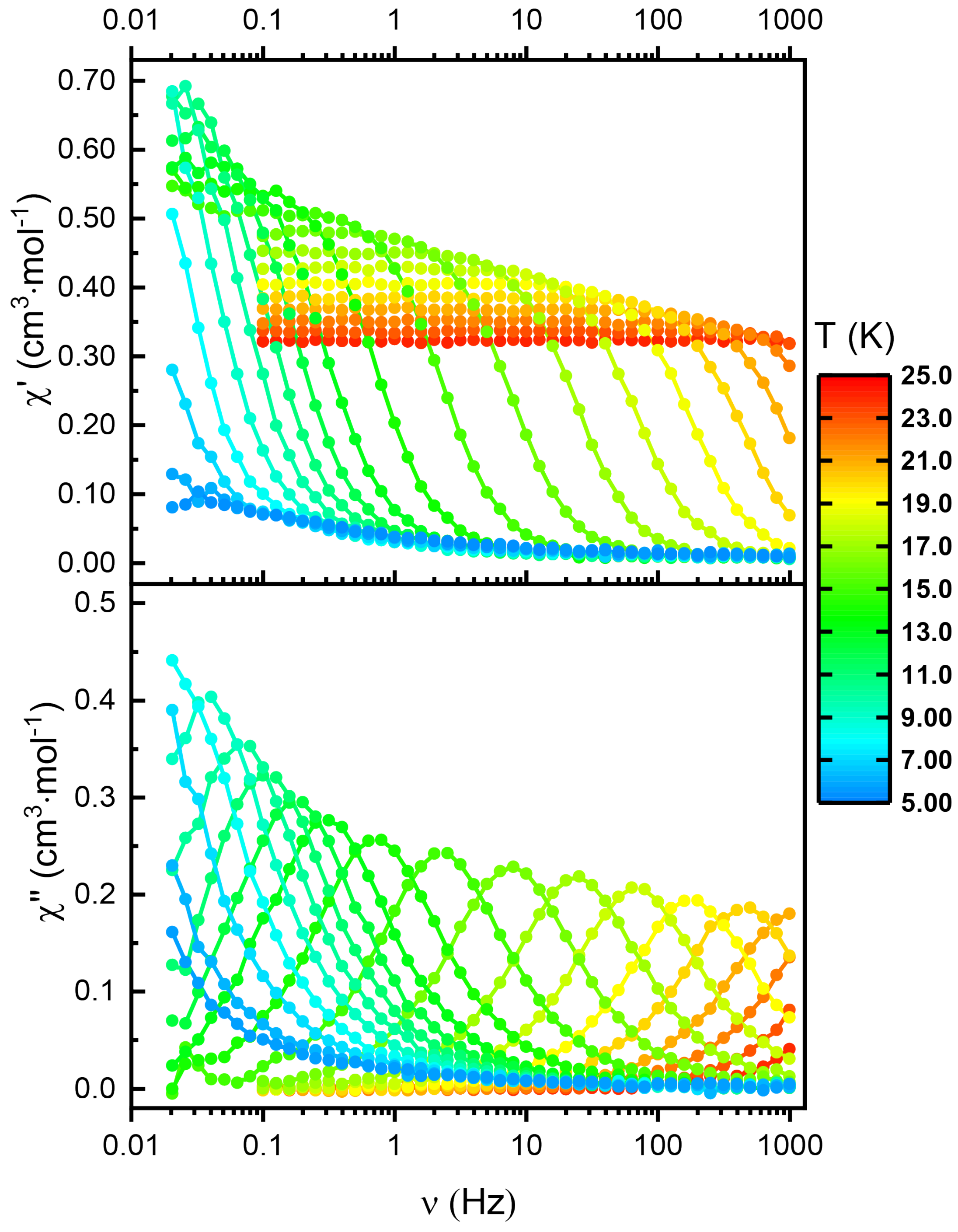

Figure S33: Frequency dependence of both in-phase (top) and out-of-phase (down) components of the AC susceptibility measured in 1000 Oe external DC field between 5 and 25 K for **Er**$^{\mathbf{ttt}}$. Circles represent experimental data points; the lines do not have any physical meaning.

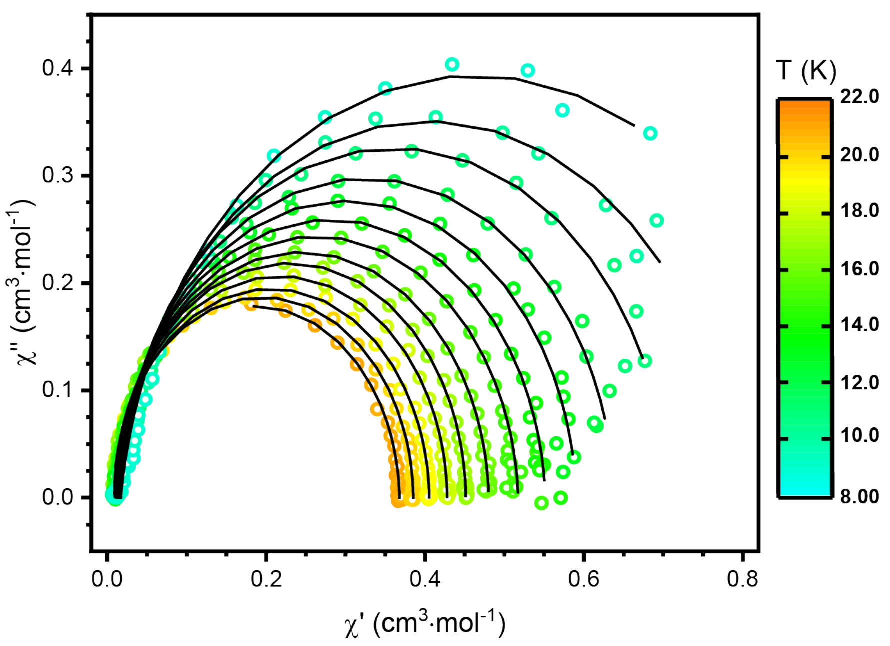

Figure S34: Variations of χ” as function of χ’ (circles) for **Er^ttt** in 1000 Oe external DC field. Circles represent experimental data points and lines represent the best fitted curves with extended Debye model.

Table S6: Best fitted parameters with the extended Debye model for **Er^ttt** at 1000 Oe external DC field.

T (K)	χ_T ($cm^3 \cdot mol^{-1}$)	χ_S ($cm^3 \cdot mol^{-1}$)	α	τ(s)	R^2
9	0.91025	0.01688	0.08056	4.29958	0.99811
10	0.79193	0.0152	0.06466	2.41686	0.99811
11	0.70792	0.0155	0.03866	1.45727	0.99805
12	0.63892	0.01367	0.03142	0.88087	0.99894
13	0.5903	0.013	0.02692	0.51049	0.99918
14	0.55127	0.01061	0.02687	0.2165	0.99948
15	0.51715	0.00937	0.02648	0.0697	0.9988
16	0.48017	0.00864	0.01877	0.02074	0.99991
17	0.45125	0.00855	0.00795	0.00658	0.99989
18	0.42794	0.00773	0.01119	0.00233	0.99988
19	0.40535	0.00736	0.01338	8.79E-04	0.99991
20	0.3852	0.00563	0.0101	3.64E-04	0.99989
21	0.36768	0.00444	0.01147	1.61E-04	0.99992

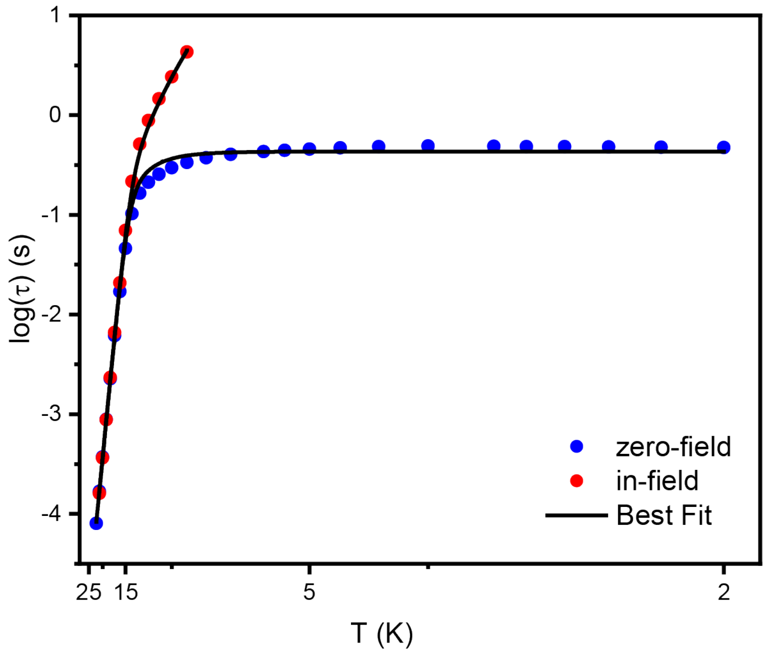

Figure S35: Temperature variation of the relaxation time (log scale) with the best fitted curve (black line) for **Er^{ttt}**. The fitting has been performed simultaneously for data collected at zero as well as at 1000 Oe external DC field.

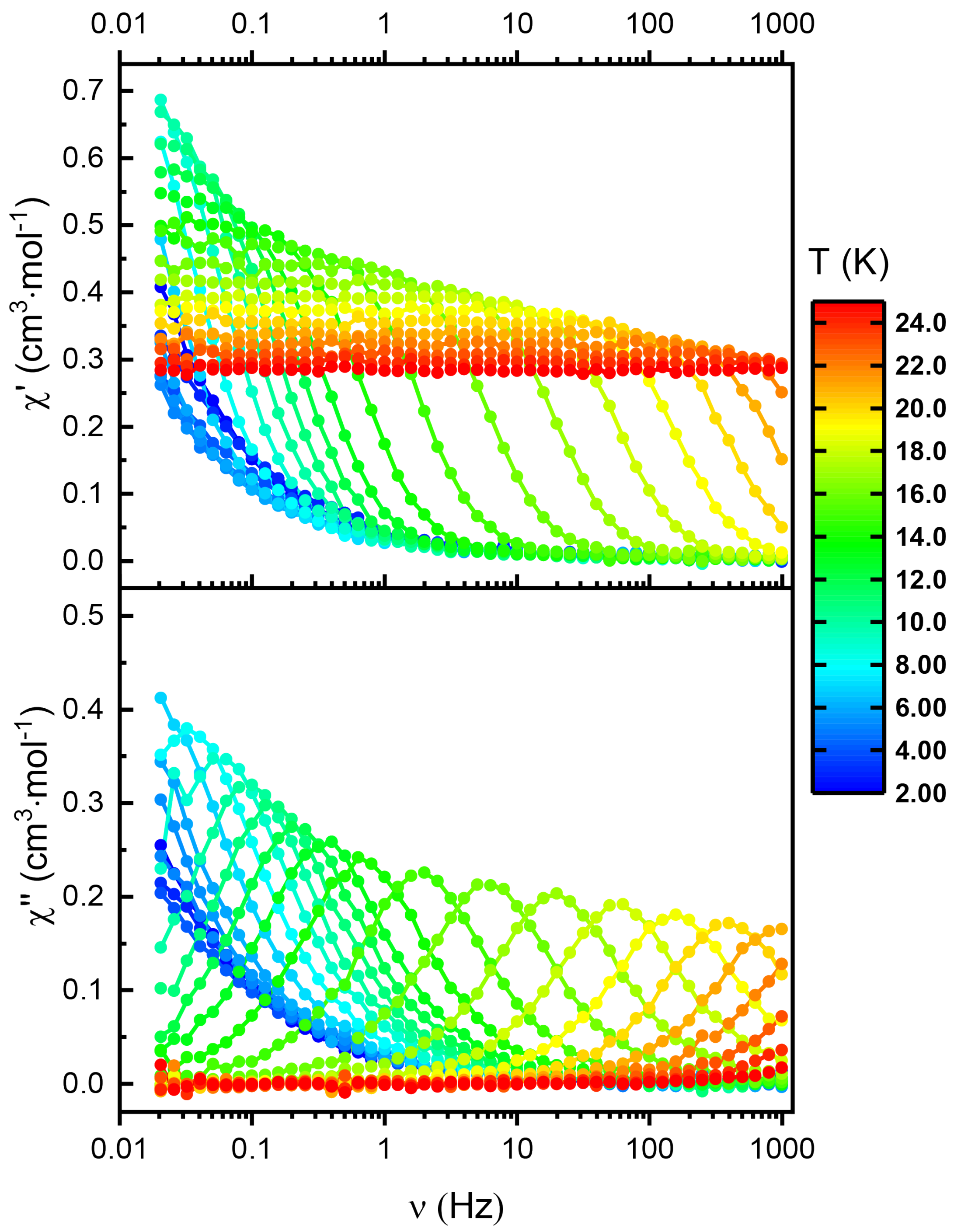

Figure S36: Frequency dependence of both in-phase (top) and out-of-phase (down) components of the AC susceptibility measured in zero external DC field between 2 and 24 K for **Er/Y$^{\mathbf{ttt}}$**. Circles represent experimental data points; the lines do not have any physical meaning.

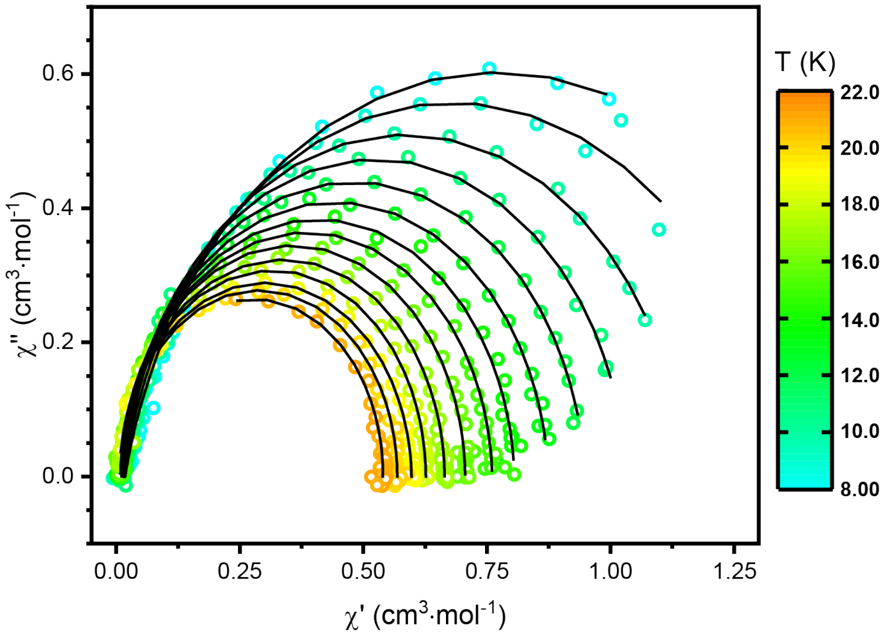

Figure S37: Variations of χ” as function of χ’ (circles) for **Er/Y^{ttt}** in zero external DC field. Circles represent experimental data points and lines represent the best fitted curves with extended Debye model.

Table S7: Best fitted parameters with the extended Debye model for **Er/Y^{ttt}** at zero external DC field.

**T (K)**	**χ_T (cm^3·mol^{-1})**	**χ_S (cm^3·mol^{-1})**	**α**	**τ(s)**	**R^2**
8	1.52188	0.0179	0.14003	5.04586	0.99894
9	1.346	0.01559	0.1124	2.85588	0.99816
10	1.15264	0.01681	0.06906	1.64837	0.99916
11	1.03357	0.01565	0.0465	1.07996	0.99934
12	0.94836	0.01367	0.03956	0.71729	0.99956
13	0.87457	0.01097	0.03452	0.46206	0.9997
14	0.80536	0.01119	0.02211	0.22878	0.99924
15	0.76052	0.01074	0.01914	0.08546	0.99913
16	0.70579	0.00864	0.0078	0.02625	0.99948
17	0.66416	0.01017	0.00693	0.00818	0.99975
18	0.62626	0.00687	0.00517	0.00272	0.99977
19	0.59738	0.00497	0.01629	0.00101	0.99969
20	0.5685	0.01053	0.00315	4.07E-4	0.99955
21	0.53956	0.00925	2.03E-17	1.77E-4	0.9996

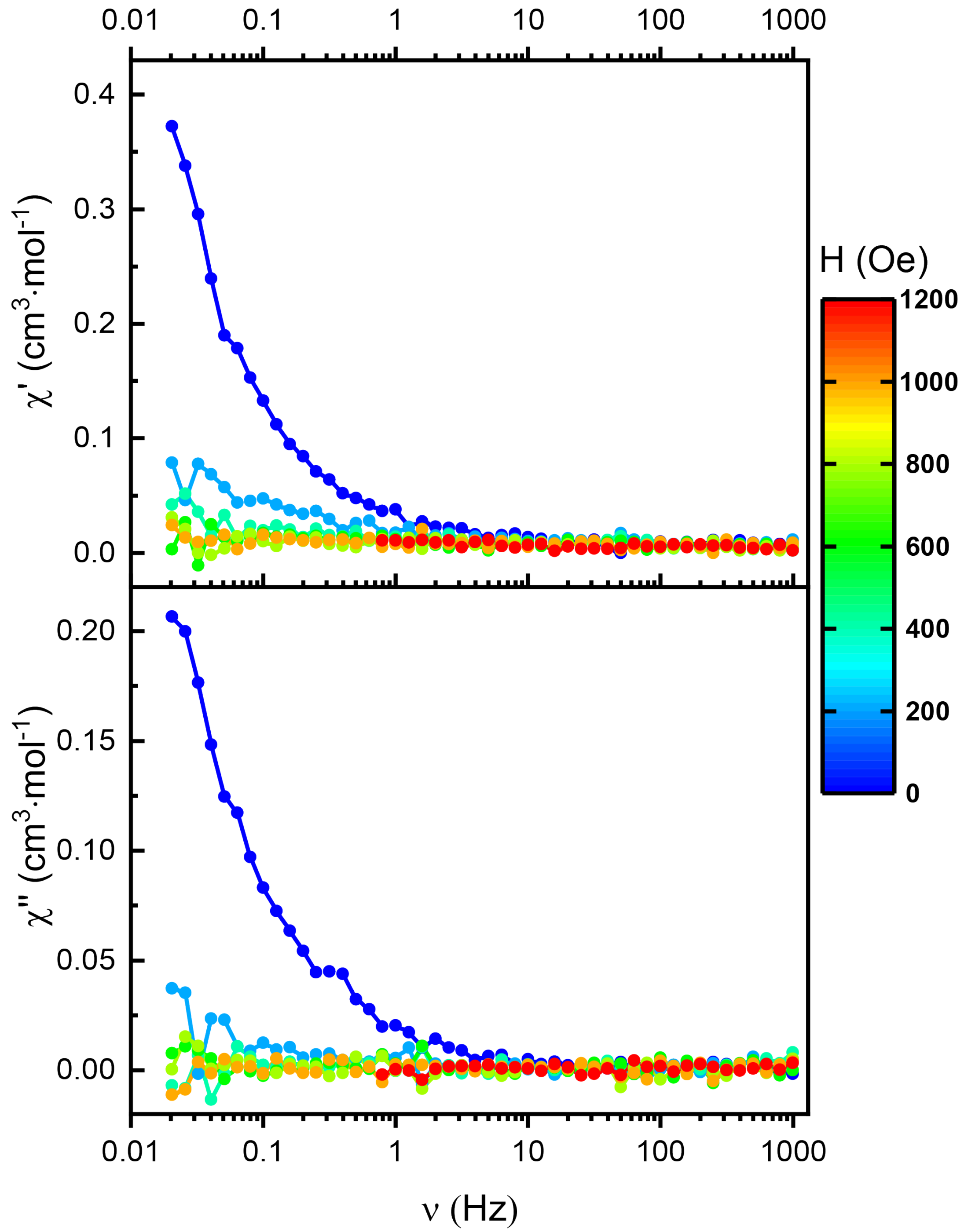

Figure S38: Frequency dependence of both in-phase (top) and out-of-phase (down) components of the AC susceptibility measured at 2 K with DC field varying between 0 and 1200 Oe for **Er/Y**$^{\mathbf{ttt}}$. Circles represent experimental data points; the lines do not have any physical meaning.

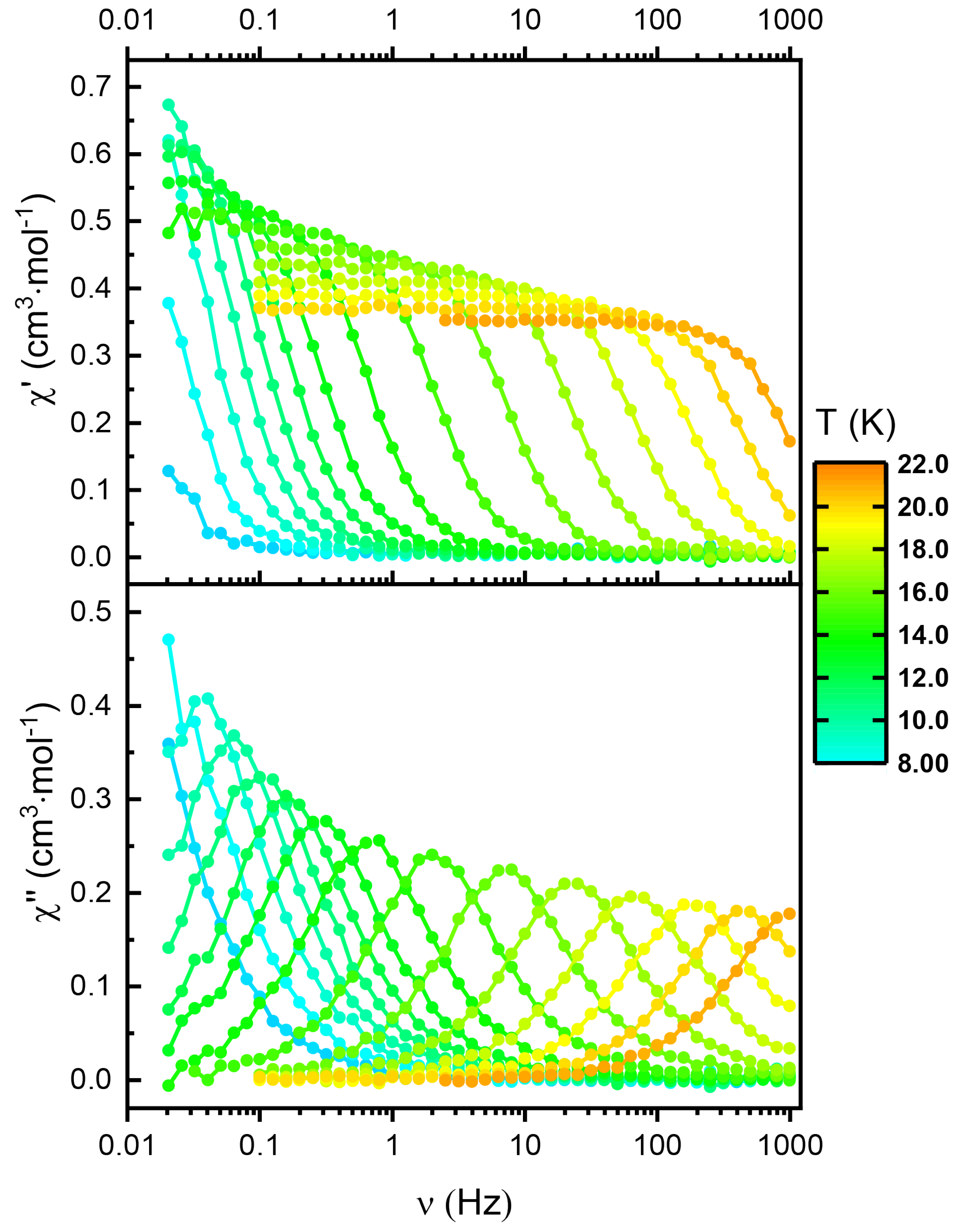

Figure S39: Frequency dependence of both in-phase (top) and out-of-phase (down) components of the AC susceptibility measured in 1000 Oe external DC field between 8 and 22 K for **Er/Y$^{\mathbf{ttt}}$**. Circles represent experimental data points; the lines do not have any physical meaning.

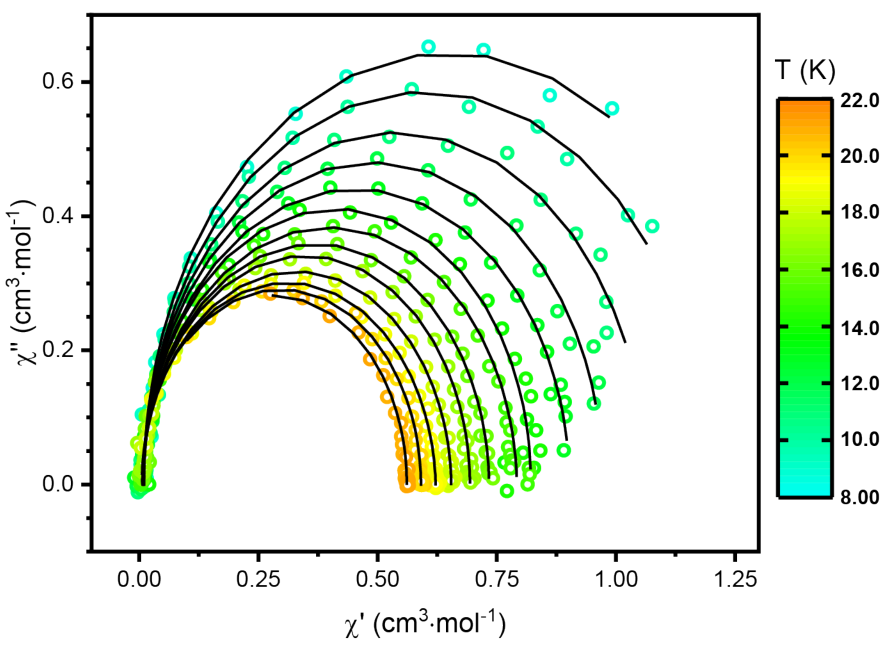

Figure S40: Variations of χ" as function of χ' (circles) for **Er/Y^{ttt}** in 1000 Oe external DC field. Circles represent experimental data points and lines represent the best fitted curves with extended Debye model.

Table S8: Best fitted parameters with the extended Debye model for **Er/Y^{ttt}** at 1000 Oe external DC field.

T (K)	χ_T ($cm^3 \cdot mol^{-1}$)	χ_S ($cm^3 \cdot mol^{-1}$)	α	τ(s)	R^2
9	1.29385	0.00983	1.44E-16	4.37154	0.99923
10	1.18945	0.00988	0.00476	2.63699	0.99916
11	1.06607	0.00829	0.00507	1.62217	0.99916
12	0.97374	0.00834	0.00337	0.98274	0.99947
13	0.90331	0.00718	0.01089	0.57012	0.99949
14	0.82171	0.00921	3.36E-14	0.23854	0.99846
15	0.79253	0.00658	0.01602	0.07779	0.99944
16	0.73403	0.00653	0.00887	0.02238	0.99966
17	0.69409	0.00559	0.00632	6.96E-3	0.99977
18	0.65483	0.0052	0.01464	2.37E-3	0.99978
19	0.62196	0	0.02082	8.86E-4	0.9997
20	0.59122	0.00363	0.00615	3.62E-4	0.99983
21	0.56142	0	4.04E-4	1.58E-4	0.99975

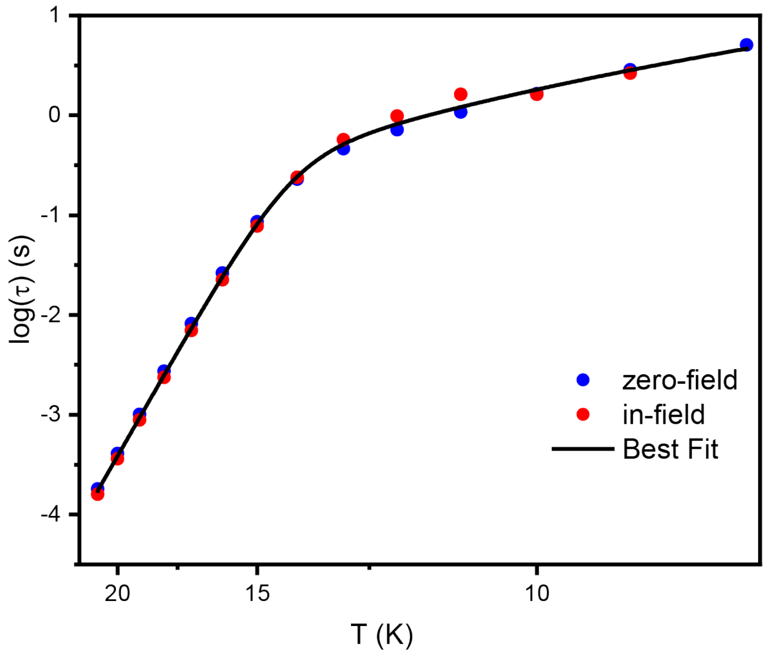

Figure S41: Temperature variation of the relaxation time (log scale) with the best fitted curve (black line) for **Er/Y$^{\mathbf{ttt}}$**. The fitting has been performed simultaneously for data collected at zero as well as at 1000 Oe external DC field.

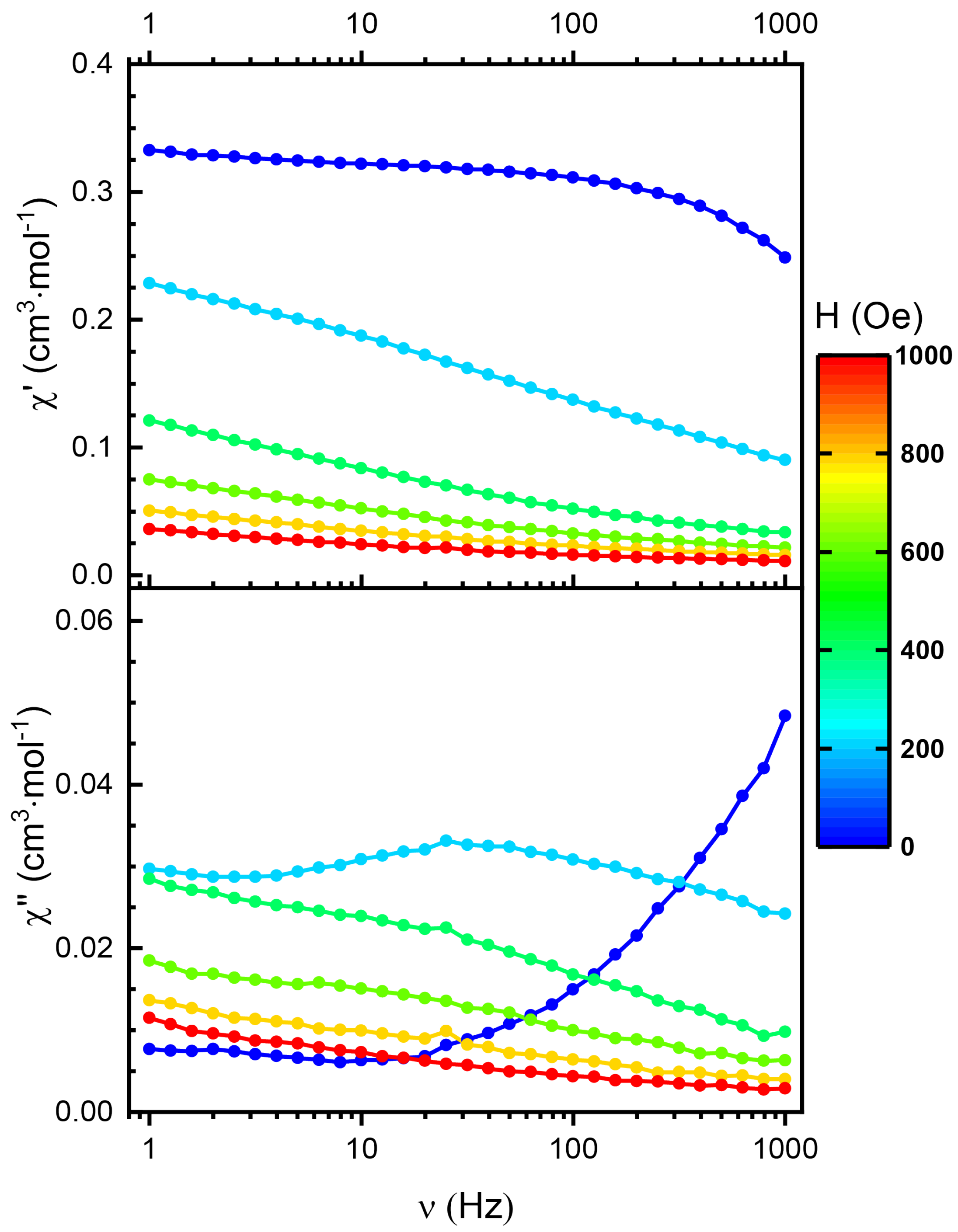

Figure S42: Frequency dependence of both in-phase (top) and out-of-phase (down) components of the AC susceptibility measured at 2 K with DC field varying between 0 and 1000 Oe for **Nd^{ttt}**. Circles represent experimental data points; the lines do not have any physical meaning.

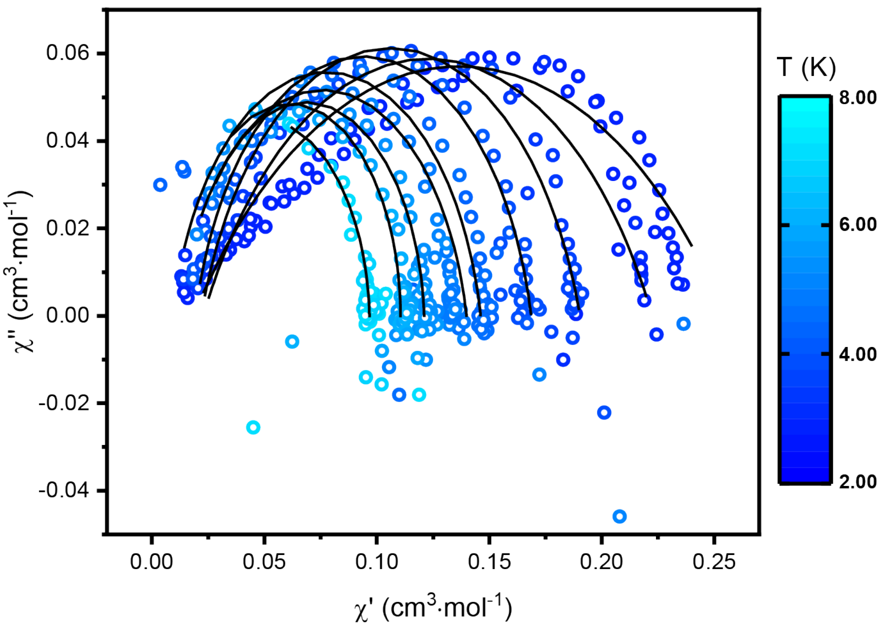

Figure S43: Variations of χ" as function of χ' (circles) for **Nd/Y^{ttt}** in zero external DC field. Circles represent experimental data points and lines represent the best fitted curves with extended Debye model.

Table S9: Best fitted parameters with the extended Debye model for **Nd/Y^{ttt}** at zero external DC field. Due to the broadening of the signal, the data between 2 to 2.6 K could not be fitted accuratly with the extended Debye model.

**T (K)**	**χ_T (cm^3·mol^{-1})**	**χ_S (cm^3·mol^{-1})**	**α**	**τ(s)**	**R^2**
2.8	0.25465	0.01858	0.4265	0.06931	0.99326
3	0.22239	0.02292	0.32184	0.03803	0.98821
3.5	0.19033	0.02195	0.19905	0.01265	0.99657
4	0.16861	0.01932	0.14481	0.0049	0.9935
4.5	0.14616	0.00925	0.12958	0.00173	0.98579
5	0.1399	0.01223	0.1338	9.72E-04	0.94206
5.5	0.12107	0.01577	0.04687	4.96E-04	0.99424
6	0.11056	0.01194	0.00961	2.90E-04	0.98511
7	0.09675	0.0027	0.03252	1.19E-04	0.97303

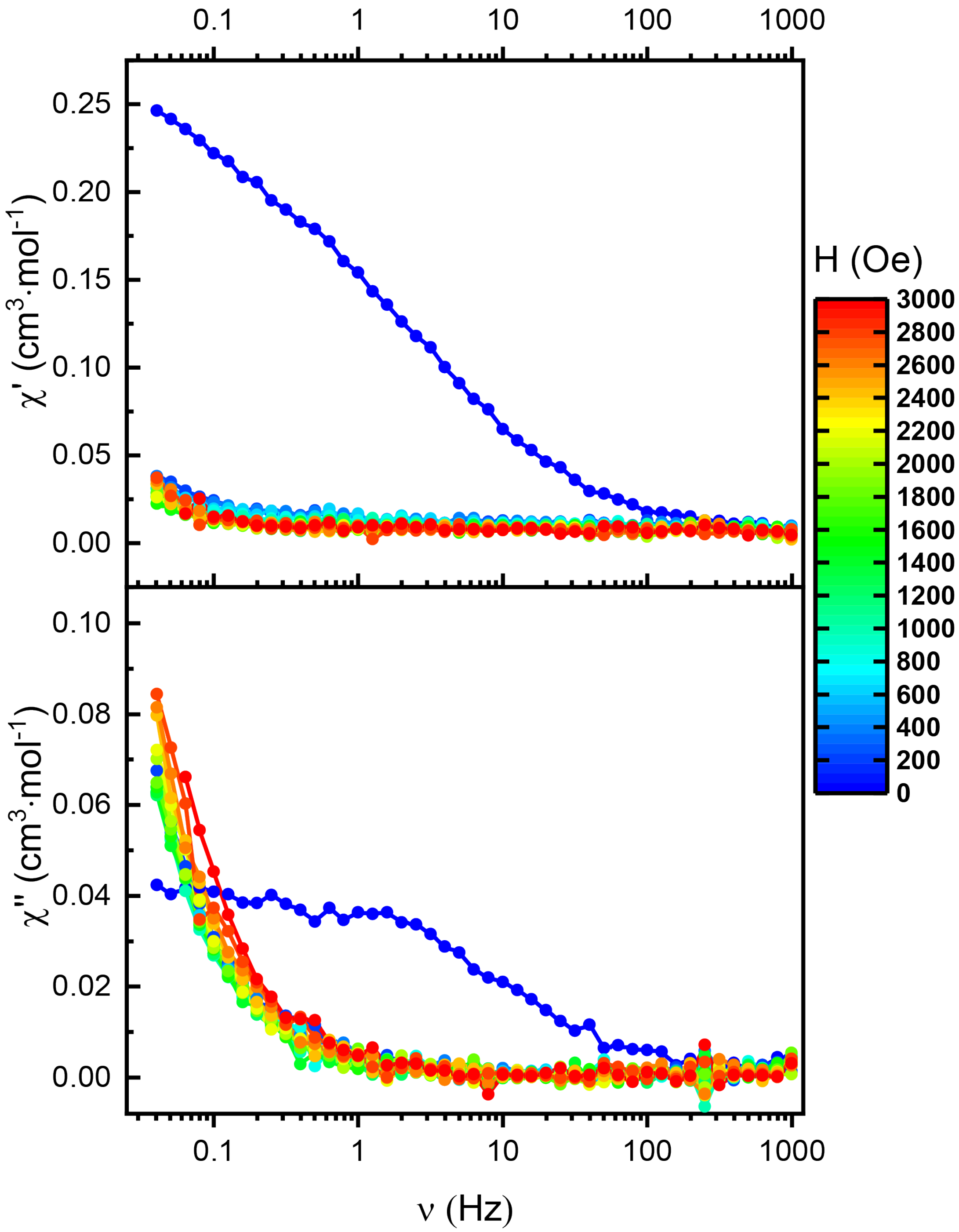

Figure S44: Frequency dependence of both in-phase (top) and out-of-phase (down) components of the AC susceptibility measured at 2 K with DC field varying between 0 and 3000 Oe for **Nd/Y^{ttt}**. Circles represent experimental data points; the lines do not have any physical meaning.

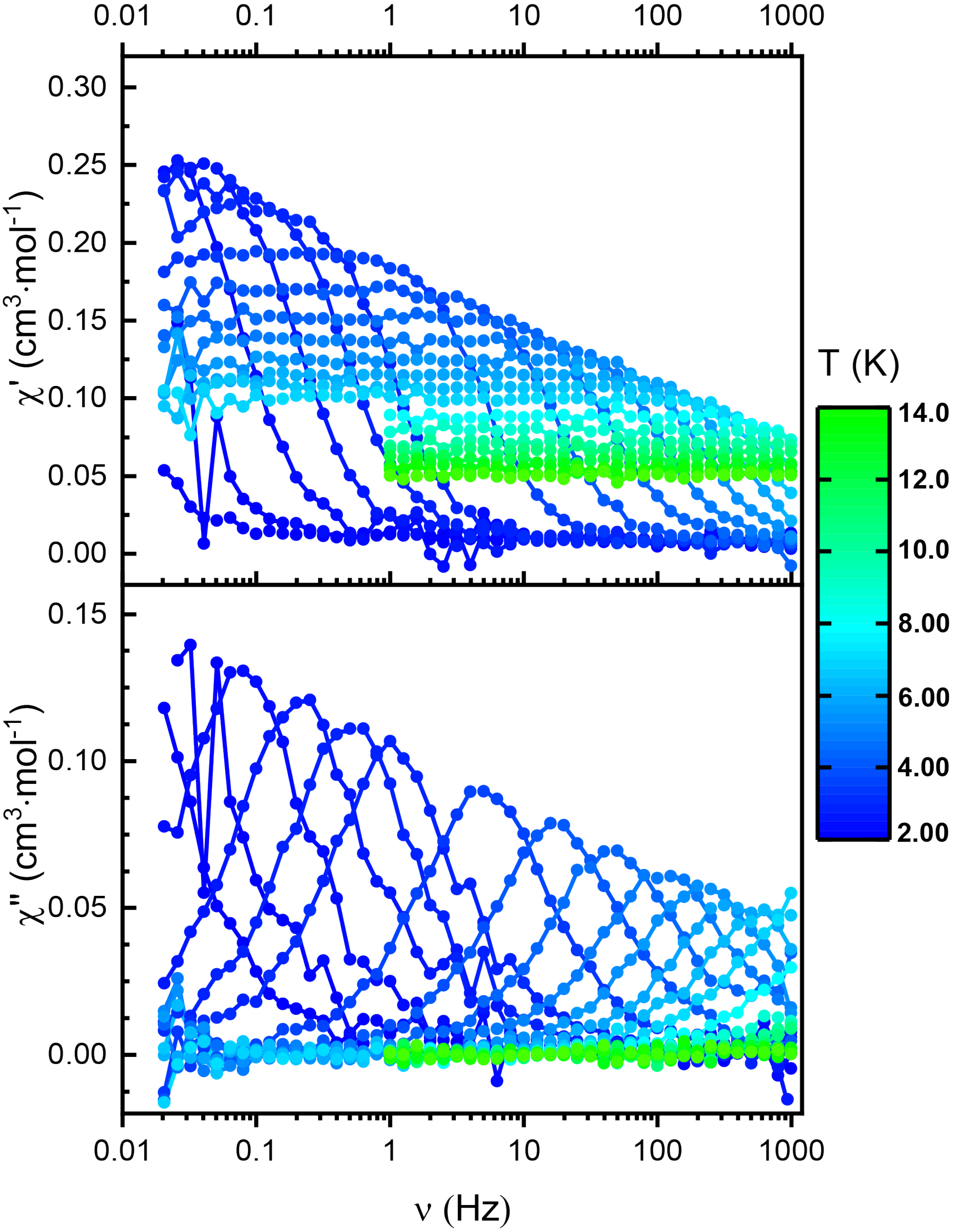

Figure S45: Frequency dependence of both in-phase (top) and out-of-phase (down) components of the AC susceptibility measured in 1000 Oe external DC field between 2 and 14 K for **Nd/Y^{ttt}**. Circles represent experimental data points; the lines do not have any physical meaning.

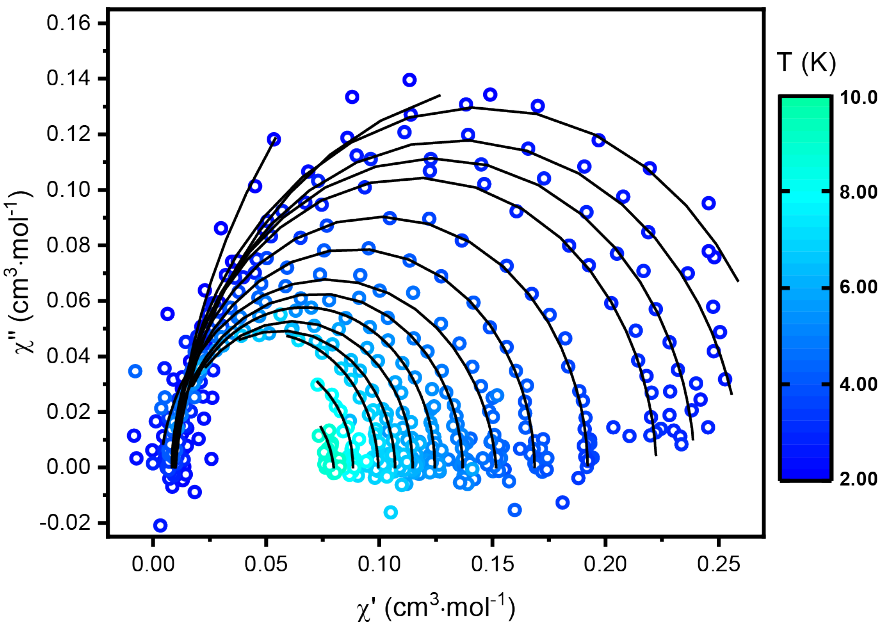

Figure S46: Variations of χ" as function of χ' (circles) for **Nd/Y^{ttt}** in 1000 Oe external DC field. Circles represent experimental data points and lines represent the best fitted curves with extended Debye model.

Table S10: Best fitted parameters with the extended Debye model for **Nd/Y^{ttt}** at 1000 Oe external DC field.

**T (K)**	**χ_T (cm^3·mol^{-1})**	**χ_S (cm^3·mol^{-1})**	**α**	**τ(s)**	**R^2**
2	0.48136	0.0097	0.05608	29.29599	0.98941
2.2	0.3184	0.0099	0.0683	8.31003	0.86855
2.4	0.2796	0.0095	0.02568	2.04724	0.99241
2.6	0.26029	0.00842	0.0418	0.7594	0.99683
2.8	0.23956	0.00895	0.02244	0.32118	0.99795
3	0.22245	0.00844	0.01613	0.15288	0.99748
3.5	0.19215	0.00826	0.01187	0.03126	0.99874
4	0.16864	0.00836	0.01139	0.00915	0.99805
4.5	0.15165	0.00312	0.05906	0.00325	0.99404
5	0.13689	0.00828	0.01706	0.00144	0.99408
5.5	0.12452	0.00911	0.00105	7.27E-04	0.99857
6	0.11475	0.00872	0.00481	4.04E-04	0.99438
6.5	0.10698	0.00545	0.01923	0.000235	0.99398
7	0.09953	0	0.02032	0.00013	0.99323
8	0.08834	0.01106	0.00584	7.94E-05	0.99858
9	0.07985	0.00987	0.09404	3.23E-05	0.99851

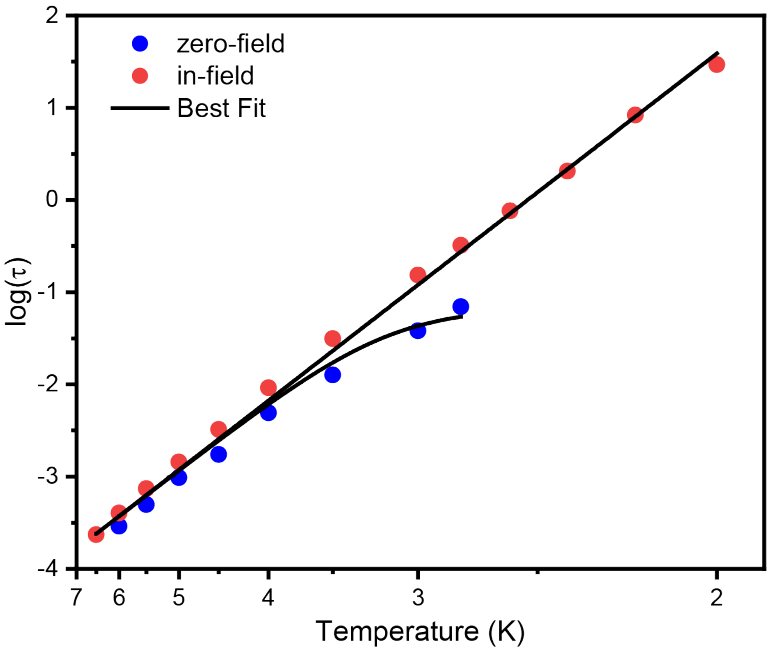

Figure S47: Temperature variation of the relaxation time (log scale) with the best fitted curve (black line) for $\mathbf{Nd/Y^{ttt}}$. The fitting has been performed simultaneously for data collected at zero as well as at 1000 Oe external DC field.

Table S11: Parameters for the relaxation processes in $\mathbf{Er^{ttt}}$, $\mathbf{Er/Y^{ttt}}$ and $\mathbf{Nd/Y^{ttt}}$ obtained from fitting of AC measurement data using $\tau^{-1} = \tau_{QTM}^{-1} + \tau_0^{-1} e^{-\frac{U_{eff}}{kT}} + CT^n$ equation, with the three terms describing quantum tunneling of magnetization, Orbach, and Raman relaxation processes, respectively.

	Applied Field [Oe]	τ_{QTM} [s]	τ_0 [s]	U_{eff} [cm^{-1}]	C [$s^{-1} \cdot K^{-n}$]	n
$\mathbf{Er^{ttt}}$	0	$(4.31 \pm 0.14) \cdot 10^{-1}$	$(2.72 \pm 1.02) \cdot 10^{-11}$	228.35±4.87	$(5.18 \pm 5.06) \cdot 10^{-7}$	5.91±0.41
	1000	-				
$\mathbf{Er/Y^{ttt}}$	0	-	$(1.74 \pm 0.54) \cdot 10^{-11}$	235.20±3.88	$(3.31 \pm 1.72) \cdot 10^{-5}$	4.22±0.22
	1000	-				
$\mathbf{Nd/Y^{ttt}}$	0	$(6.73 \pm 1.50) \cdot 10^{-2}$	$(1.16 \pm 0.18) \cdot 10^{-6}$	24.09±0.36	-	-
	1000	-				

Table S12: Computed energy levels (the ground state is set at zero), composition of the g-tensor and contributions to the wavefunction for each M_J state of the ground-state multiplet for **Dy^{ttt}**. KD stands for Kramers doublet.

KD	E (cm^{-1})	g_x	g_y	g_z	Wavefunction composition*
1	0.000	0.02341	0.05906	15.99534	45.7% |±15/2> + 24.1% |±13/2>
2	32.032	0.06794	0.10366	12.50436	20.7% |±5/2> + 18.9% |±11/2> + 17.6% |±3/2> + 17.4% |±9/2>
3	54.071	0.05010	0.09968	12.75811	42.0% |±1/2> + 20.6% |±15/2> + 18.3% |±3/2>
4	106.937	0.78482	1.08087	6.75043	30.8% |±3/2> + 17.2% |±11/2> + 16.3% |±1/2> + 12.4% |±13/2> + 12.1% |±5/2>
5	138.150	0.00627	0.03146	17.23323	23.6% |±13/2> + 22.7% |±15/2> + 13.9% |±5/2> + 12.5% |±7/2> + 11.1% |±3/2>
6	175.344	3.29418	3.77183	4.86548	33.2% |±5/2> + 14.9% |±3/2> + 14.0 % |±9/2> + 13.4% |±11/2> + 13.2% |±11/2>
7	223.896	1.11316	6.28228	14.48991	32.8% |±7/2> + 28.7% |±9/2>
8	290.091	0.00039	0.00044	19.88579	26.2% |±9/2> + 25.6% |±11/2> + 17.6% |±7/2> + 14.8% |±13/2>

* Contributions < 10% are omitted.

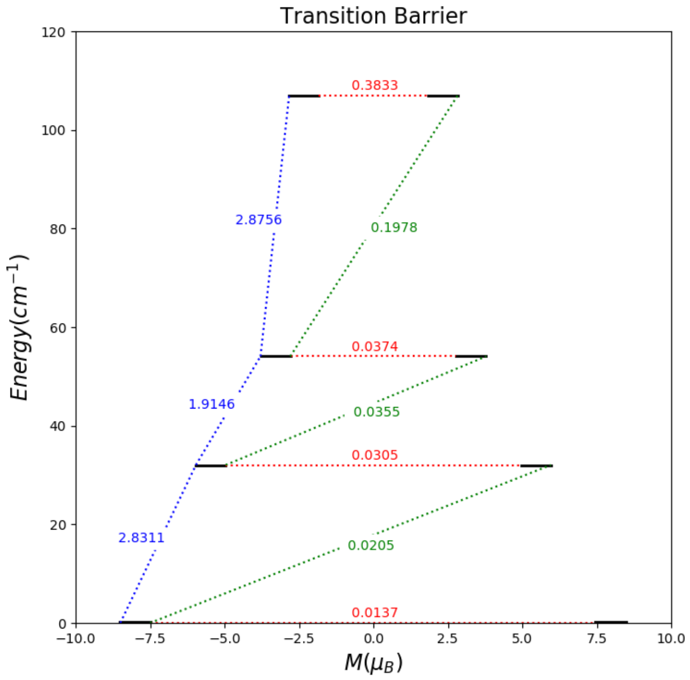

Figure S48: Computed magnetization blocking barriers for **Dy^{ttt}**. The four lowest Kramers doublets (thick black lines) are represented according to their magnetic moment along the main magnetic axis. The blue lines represent vertical excitations, the green dashed lines correspond to possible Orbach relaxation processes while the red lines correspond to QTM/TA-QTM processes. The values correspond to the mean value of the corresponding transversal matrix element of the transition magnetic moment.

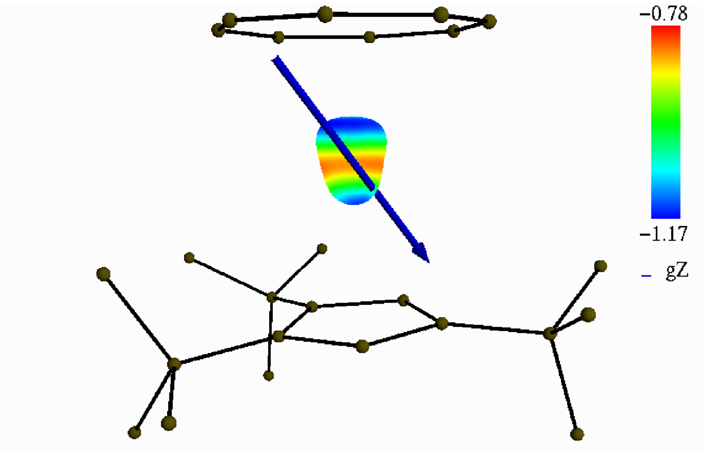

Figure S49: Representation of the total electrostatic potential (expressed in $e^{-}\cdot bohr^{-1}$) at 2.5 Å around the Dy(III) ion with g_z direction in blue line for **Dy^{ttt}**. The lowest and highest values are in blue and red, respectively.

Table S13: Computed energy levels (the ground state is set at zero), composition of the g-tensor and contributions to the wavefunction for each M_J state of the ground-state multiplet for $\mathbf{Er^{ttt}}$. KD stands for Kramers doublet.

KD	E (cm^{-1})	g_x	g_y	g_z	Wavefunction composition*
1	0.000	0.00038	0.00061	17.92160	99.7% |±15/2>
2	116.385	0.02707	0.95897	14.44822	50.0% |±5/2> + 36.6% |±3/2>
3	131.682	2.85256	4.87165	9.02133	48.8% |±7/2> + 17.1% |±1/2> + 14.0% |±5/2> + 13.4% |±3/2>
4	146.416	0.06555	3.27707	7.71716	35.1% |±1/2> +30.2% |±7/2> + 16.4% |±5/2>
5	154.626	0.29224	3.13523	12.95059	37.1% |±3/2> + 27.0% |±1/2> + 17.7% |±5/2> + 10.0% |±9/2>
6	170.469	0.79339	1.88597	9.20432	48.2% |±9/2> + 14.9% |±11/2> + 12.2% |±1/2> + 10.6% |±7/2>
7	203.719	0.47265	0.54927	13.92806	61.1% |±13/2> + 20.8% |±9/2> + 15.1% |±11/2>
8	249.882	0.05861	0.08792	16.17964	62.8% |±11/2> + 29.4% |±13/2>

* Contributions $<$ 10% are omitted.

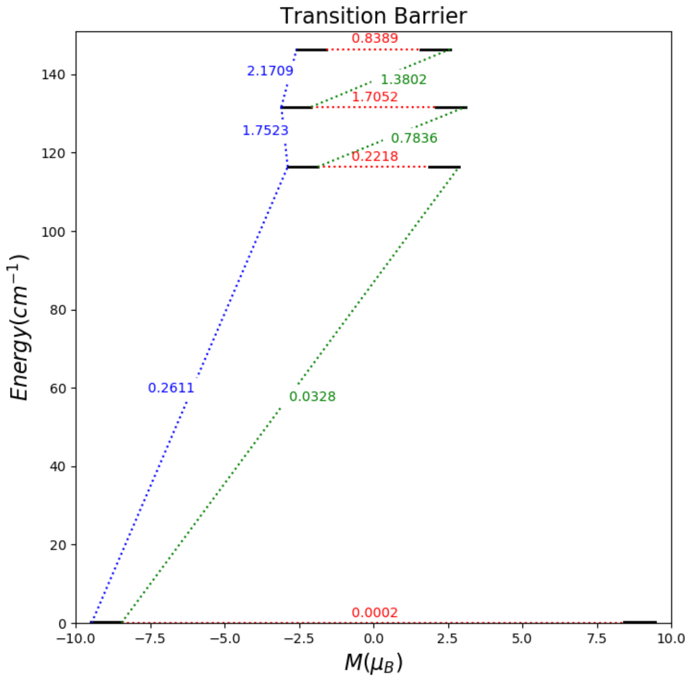

Figure S50: Computed magnetization blocking barriers for **Er^{ttt}**. The four lowest Kramers doublets (thick black lines) are represented according to their magnetic moment along the main magnetic axis. The blue lines represent vertical excitations, the green dashed lines correspond to possible Orbach relaxation processes while the red lines correspond to QTM/TA-QTM processes. The values correspond to the mean value of the corresponding transversal matrix element of the transition magnetic moment.

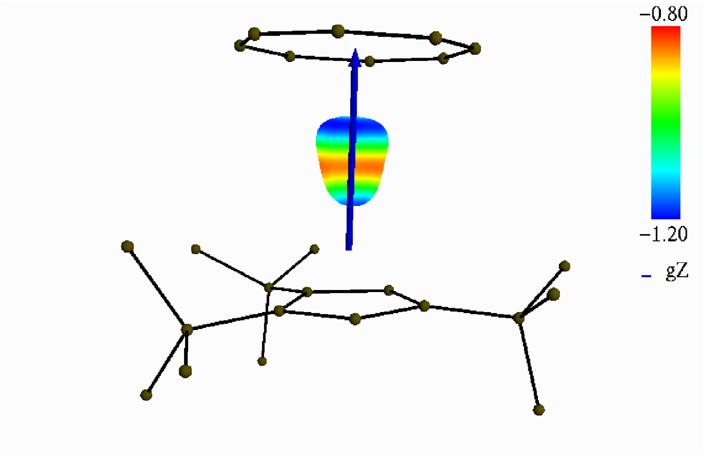

Figure S51: Representation of the total electrostatic potential (expressed in e^{-}·bohr^{-1}) at 2.5 Å around the Er(III) ion with g_z direction in blue line for **Er^{ttt}**. The lowest and highest values are in blue and red, respectively.

Table S14: Computed energy levels (the ground state is set at zero), composition of the g-tensor and contributions to the wavefunction for each M_J state of the ground-state multiplet for $\mathbf{Nd^{ttt}}$. KD stands for Kramers doublet.

KD	E (cm^{-1})	g_x	g_y	g_z	Wavefunction composition*
CASSCF					
1	0.000	0.02627	0.03048	5.07946	66.7% |±7/2> + 18.3% |±9/2> + 14.3% |±5/2>
2	244.010	3.12894	2.37193	1.05699	69.0% |±1/2> + 16.2% |±9/2>
3	263.209	0.03642	0.43703	4.83868	52.1% |±9/2> + 17.1% |±7/2> + 15.1% |±3/2> + 14.5% |±1/2>
4	293.055	0.44327	0.77708	4.24601	45.6% |±3/2> + 32.0% |±5/2> + 12.0% |±9/2>
5	356.195	0.23192	0.64409	4.00103	51.7% |±5/2> + 30.7 % |±3/2>
CASPT2					
1	0.000	0.16336	0.74274	4.57316	39.3% |±7/2> + 26.9% |±9/2> + 25.0% |±5/2>
2	78.216	0.58869	1.45157	4.19800	41.4% |±3/2> + 36.9% |±5/2> + 11.0% |±7/2>
3	211.365	0.68740	1.35621	3.13192	43.4% |±3/2> + 25.8% |±7/2> + 24.6% |±5/2>
4	393.745	0.11565	0.92790	5.35153	48.4% |±9/2> + 19.3% |±7/2> + 17.6% |±1/2>
5	545.431	0.12793	1.40835	4.08258	65.1% |±1/2> + 20.2% |±9/2>

* Contributions < 10% are omitted.

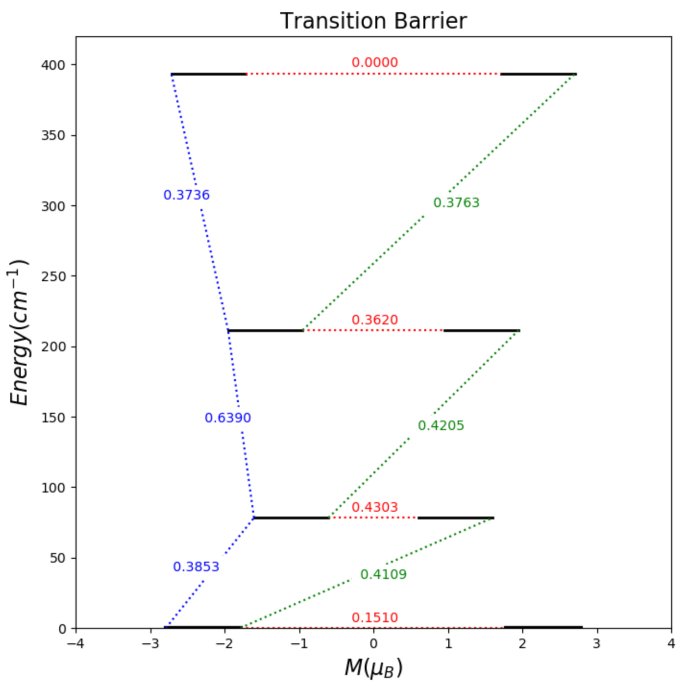

Figure S52: Computed (CASPT2) magnetization blocking barriers for $\mathbf{Nd^{ttt}}$. The four lowest Kramers doublets (thick black lines) are represented according to their magnetic moment along the main magnetic axis. The blue lines represent vertical excitations, the green dashed lines correspond to possible Orbach relaxation processes while the red lines correspond to QTM/TA-QTM processes. The values correspond to the mean value of the corresponding transversal matrix element of the transition magnetic moment.

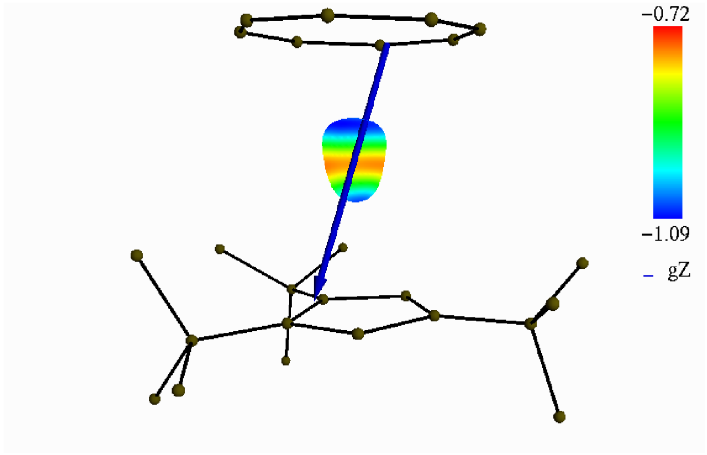

Figure S53: Representation of the total electrostatic potential (expressed in $e^{-}\cdot bohr^{-1}$, CASPT2) at 2.5 Å around the Nd(III) ion with g_z direction in blue line for $\mathbf{Nd^{ttt}}$. The lowest and highest values are in blue and red, respectively.

Cartesian coordinates of computational models

(COT)Er(Cp^{ttt})

Er	3.862867	3.424654	6.239707
C	2.895589	2.637389	3.969895
C	2.8978	4.057951	3.884673
C	1.964936	2.198061	4.926653
C	1.923468	4.523241	4.838135
C	3.716899	4.73825	2.784469
C	2.847259	4.855452	1.525725
C	4.929842	3.875916	2.413573
C	4.276	6.111051	3.148674
C	1.390491	3.363847	5.467004
C	1.519789	0.774217	5.173255
C	1.35858	5.910898	5.159017
C	0.354002	0.481295	4.220748
C	0.811976	6.581162	3.895958
C	2.64749	-0.213554	4.889097
C	2.367966	6.828214	5.855887
C	1.039147	0.586305	6.609786
C	0.172456	5.787579	6.120455
C	3.999107	2.516258	8.573752
C	3.98191	3.918418	8.704811
H	3.265134	2.031043	9.206227
H	3.241655	4.259592	9.418945
C	4.690549	1.604287	7.751701
C	4.645683	4.984226	8.065381
H	4.365712	0.582047	7.905848
H	4.297509	5.950184	8.410236
C	5.63246	1.715561	6.710754
C	5.576431	5.097232	7.015382
H	5.855523	0.758726	6.253848
H	5.768604	6.128676	6.744289
C	6.276336	2.783605	6.054442
C	6.249267	4.185975	6.176476
H	6.871682	2.450877	5.212511
H	6.832844	4.681388	5.409449

H	4.893444	6.479215	2.326064
H	4.908218	6.052576	4.038017
H	3.501873	6.852106	3.326229
H	5.532684	4.403229	1.671362
H	4.641348	2.921235	1.9741
H	5.559466	3.677951	3.283429
H	3.424349	5.293471	0.706602
H	1.968181	5.475728	1.691721
H	2.502661	3.868407	1.212062
H	3.295264	6.940421	5.302376
H	2.615964	6.430346	6.841926
H	1.935915	7.82243	5.998999
H	1.591265	6.841562	3.184061
H	0.290618	7.504019	4.162527
H	0.101953	5.923276	3.390915
H	-0.63393	5.187797	5.695049
H	-0.223071	6.783979	6.327714
H	0.465906	5.347324	7.075489
H	2.986753	-0.146689	3.853863
H	2.302677	-1.236538	5.056334
H	3.5054	-0.032684	5.539519
H	0.199908	1.2452	6.839753
H	1.838749	0.794945	7.323454
H	0.703231	-0.441691	6.764401
H	0.662877	0.609363	3.181567
H	-0.479291	1.161126	4.40835
H	-0.00198	-0.544273	4.350726
H	3.509144	1.986539	3.368362
H	0.622764	3.374487	6.223482

(COT)Er(Cp*)

Er	3.74495	3.310437	6.173009
C	2.895982	2.602349	3.848323
C	2.89618	4.021279	3.850288
C	1.917728	2.163234	4.776709
C	1.915217	4.459439	4.776698
C	3.732905	1.728021	2.97249

C	3.734995	4.897065	2.977877
H	3.237494	1.531924	2.016053
H	3.242455	5.093303	2.020037
H	4.697791	2.185441	2.74657
H	4.701085	4.440783	2.754789
H	3.931126	0.75997	3.435648
H	3.930669	5.864933	3.442492
C	1.312077	3.311183	5.351289
C	1.542168	0.743434	5.050448
C	1.539294	5.878881	5.051913
H	0.742831	0.407453	4.382064
H	0.737574	6.214509	4.386164
H	2.386153	0.06645	4.907586
H	2.382304	6.556533	4.906468
H	1.184141	0.607449	6.072457
H	1.18444	6.014565	6.075104
C	0.186764	3.310509	6.333545
H	0.212369	4.188275	6.981792
H	0.209403	2.429268	6.977108
C	3.69189	2.608536	8.574596
C	3.691758	4.019035	8.573079
H	2.907697	2.196198	9.198394
H	2.907653	4.432953	9.196035
C	4.411083	1.609592	7.885797
C	4.409083	5.015397	7.878809
H	4.047002	0.613078	8.10644
H	4.045183	6.012835	8.095596
C	5.424786	1.607849	6.905601
C	5.426503	5.014073	6.902161
H	5.65739	0.610275	6.551979
H	5.659499	6.010523	6.545765
C	6.143298	2.604552	6.212557
C	6.144713	4.014858	6.212778
H	6.796249	2.190038	5.453673
H	6.796227	4.427175	5.451408
H	-0.783496	3.313544	5.826309

References

[1] E. V. Dehmlow and C. Bollmann, *Z. Naturforsch.*, 1993, **48b**, 457–460.

[2] W. J. Evans, S. A. Kozimor, J. W. Ziller, A. A. Fagin and M. N. Bochkarev, *Inorg. Chem.*, 2005, **44**, 3993–4000.

[3] S. Kobayashi and I. Hachiya, *J. Org. Chem.*, 1994, **59**, 3590–3596.

[4] U. Kilimann and F. T. Edelmann, *J. Organomet. Chem.*, 1994, **469**, C5–C9.

[5] K. Momma and F. Izumi, *J. Appl. Crystallogr.*, 2011, **44**, 1272–1276.

[6] G. R. Fulmer, A. J. M. Miller, N. H. Sherden, H. E. Gottlieb, A. Nudelman, B. M. Stoltz, J. E. Bercaw and K. I. Goldberg, *Organometallics*, 2010, **29**, 2176–2179.

[7] G. M. Sheldrick, *Acta Cryst.*, 2008, **A63**, 112–122.

[8] G. M. Sheldrick, *Acta Cryst.*, 2015, **A71**, 3–8.

[9] G. M. Sheldrick, *Acta Cryst.*, 2015, **C71**, 3–8.

[10] O. V. Dolomanov, L. J. Bourhis, R. J. Gildea, J. A. K. Howard and H. Puschmann, *J. Appl. Crystallogr.*, 2009, **42**, 339–341.

[11] G. A. Bain and J. F. Berry, *J. Chem. Educ.*, 2008, **85**, 532–536.

[12] F. Neese, *WIREs Comput. Mol. Sci.*, 2012, **2**, 73–78.

[13] F. Neese, *WIREs Comput. Mol. Sci.*, 2018, **8**, e1327.

[14] F. Neese, F. Wennmohs, U. Becker and C. Riplinger, *J. Chem. Phys.*, 2020, **152**, 224108.

[15] C. Adamo and V. Barone, *J. Chem. Phys.*, 1999, **110**, 6158–6170.

[16] E. van Lenthe, E. J. Baerends and J. G. Snijders, *J. Chem. Phys.*, 1993, **99**, 4597–4610.

[17] E. van Lenthe, E. J. Baerends and J. G. Snijders, *J. Chem. Phys.*, 1994, **101**, 9783–9792.

[18] E. van Lenthe, A. Ehlers and E.-J. Baerends, *J. Chem. Phys.*, 1999, **110**, 8943–8953.

[19] F. Weigend and R. Ahlrichs, *Phys. Chem. Chem. Phys.*, 2005, **7**, 3297–3305.

[20] D. A. Pantazis, X.-Y. Chen, C. R. Landis and F. Neese, *J. Chem. Theory Comput.*, 2008, **4**, 908–919.

[21] D. A. Pantazis and F. Neese, *J. Chem. Theory Comput.*, 2009, **5**, 2229–2238.

[22] J. D. Rolfes, F. Neese and D. A. Pantazis, *J. Comput. Chem.*, 2020, **41**, 1842–1849.

[23] F. Weigend, *Phys. Chem. Chem. Phys.*, 2006, **8**, 1057–1065.

[24] F. Neese, F. Wennmohs, A. Hansen and U. Becker, *Chem. Phys.*, 2009, **356**, 98–109.

[25] C. Kollmar, *Int. J. Quantum Chem.*, 1997, **62**, 617–637.

[26] O. Takahashi, K. Saito and S. Yabushita, *Int. J. Quantum Chem.*, 1999, **74**, 515–530.

[27] S. Grimme, J. Antony, S. Ehrlich and H. Krieg, *J. Chem. Phys.*, 2010, **132**, 154104.

[28] S. Grimme, S. Ehrlich and L. Goerigk, *J. Comput. Chem.*, 2011, **32**, 1456–1465.

[29] I. Fdez. Galván, M. Vacher, A. Alavi, C. Angeli, F. Aquilante, J. Autschbach, J. J. Bao, S. I. Bokarev, N. A. Bogdanov, R. K. Carlson, L. F. Chibotaru, J. Creutzberg, N. Dattani, M. G. Delcey, S. S. Dong, A. Dreuw, L. Freitag, L. M. Frutos, L. Gagliardi, F. Gendron, A. Giussani, L. González, G. Grell, M. Guo, C. E. Hoyer, M. Johansson, S. Keller, S. Knecht, G. Kovaăević, E. Källman, G. Li Manni, M. Lundberg, Y. Ma, S. Mai, J. a. P. Malhado, P. A. k. Malmqvist, P. Marquetand, S. A. Mewes, J. Norell, M. Olivucci, M. Oppel, Q. M. Phung, K. Pierloot, F. Plasser, M. Reiher, A. M. Sand, I. Schapiro, P. Sharma, C. J. Stein, L. K. Sørensen, D. G. Truhlar, M. Ugandi, L. Ungur, A. Valentini, S. Vancoillie, V. Veryazov, O. Weser, T. A. Wesołowski, P.-O. Widmark, S. Wouters, A. Zech, J. P. Zobel and R. Lindh, *J. Chem. Theory Comput.*, 2019, **15**, 5925–5964.

[30] B. O. Roos, P. R. Taylor and P. E. Sigbahn, *Chem. Phys.*, 1980, **48**, 157–173.

[31] P.-Å. Malmqvist and B. O. Roos, *Chem. Phys. Lett.*, 1989, **155**, 189–194.

[32] P. Å. Malmqvist, B. O. Roos and B. Schimmelpfennig, *Chem. Phys. Lett.*, 2002, **357**, 230–240.

[33] B. O. Roos, R. Lindh, P.-Å. Malmqvist, V. Veryazov and P.-O. Widmark, *J. Phys. Chem. A*, 2004, **108**, 2851–2858.

[34] B. O. Roos, R. Lindh, P.-Å. Malmqvist, V. Veryazov, P.-O. Widmark and A. C. Borin, *J. Phys. Chem. A*, 2008, **112**, 11431–11435.

[35] F. Aquilante, P.-Å. Malmqvist, T. B. Pedersen, A. Ghosh and B. O. Roos, *J. Chem. Theory and Comput.*, 2008, **4**, 694–702.

[36] L. F. Chibotaru and L. Ungur, *J. Chem. Phys.*, 2012, **137**, 064112.

[37] K. Andersson, P.-Å. Malmqvist, B. O. Roos, A. J. Sadlej and K. Wolinski, *J. Phys. Chem.*, 1990, **94**, 5483–5488.

[38] K. Andersson, P. k. Malmqvist and B. O. Roos, *J. Chem. Phys.*, 1992, **96**, 1218–1226.

[39] G. Ghigo, B. O. Roos and P.-Å. Malmqvist, *Chem. Phys. Lett.*, 2004, **396**, 142–149.

[40] N. Forsberg and P.-Å. Malmqvist, *Chem. Phys. Lett.*, 1997, **274**, 196–204.

[41] L. Gagliardi, R. Lindh and G. Karlström, *J. Chem. Phys.*, 2004, **121**, 4494–4500.

[42] K. Zhang, V. Montigaud, O. Cador, G.-P. Li, B. Le Guennic, J.-K. Tang and Y.-Y. Wang, *Inorg. Chem.*, 2018, **57**, 8550–8557.

[43] G. Huang, G. Fernandez-Garcia, I. Badiane, M. Camarra, S. Freslon, O. Guillou, C. Daiguebonne, F. Totti, O. Cador, T. Guizouarn, B. Le Guennic and K. Bernot, *Chem. Eur. J.*, 2018, **24**, 6983–6991.

[44] L. Falivene, Z. Cao, A. Petta, L. Serra, A. Poater, R. Oliva, V. Scarano and L. Cavallo, *Nat. Chem.*, 2019, **11**, 872–879.

[45] H. Clavier and S. P. Nolan, *Chem. Commun.*, 2010, **46**, 841–861.
